# Controlling the Site Selectivity in Acylations of Amphiphilic Diols: Directing the Reaction toward the Apolar Domain in a Model Diol and the Midecamycin A_1_ Macrolide Antibiotic

**DOI:** 10.1021/acs.joc.2c00745

**Published:** 2022-07-08

**Authors:** Reut Fallek, Natali Ashush, Amit Fallek, Or Fleischer, Moshe Portnoy

**Affiliations:** School of Chemistry, Raymond and Beverly Sackler Faculty of Exact Sciences, Tel Aviv University, Tel Aviv 6997801, Israel

## Abstract

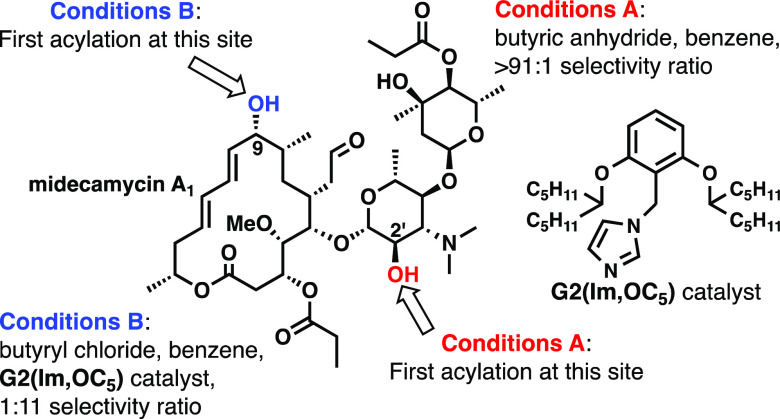

Seeking to improve the site selectivity of acylation of amphiphilic diols, which is induced by imidazole-based nucleophilic catalysts and directs the reaction toward apolar sites, as we recently reported, we examined a new improved catalytic design and an alteration of the acylating agent. The new catalysts performed slightly better selectivity-wise in the model reaction, compared to the previous set, but notably could be prepared in a much more synthetically economic way. The change of the acylating agent from anhydride to acyl chloride, particularly in combination with the new catalysts, accelerated the reaction and increased the selectivity in favor of the apolar site. The new selectivity-inducing techniques were applied to midecamycin, a natural amphiphilic antibiotic possessing a secondary alcohol moiety in each of its two domains, polar as well as apolar. In the case of the anhydride, a basic dimethylamino group, decorating this substrate, overrides the catalyst’s selectivity preference and forces selective acylation of the alcohol in the polar domain with a more than 91:1 ratio of the monoacylated products. To counteract the internal base influence, an acid additive was used or the acylating agent was changed to acyl chloride. The latter adjustment leads, in combination with our best catalyst, to the reversal of the ratio between the products to 1:11.

## Introduction

Site-selective chemical modifications of multifunctional compounds, particularly of natural products, have emerged in the past decade as one of the most important developments in organic synthetic chemistry and promise significant benefits in the field of medicinal chemistry as well.^1^ “Pinpointed” modification of such a compound can alter its therapeutic and toxicity profiles, potentially leading to a new semi-synthetic drug, or contribute significantly to the study of its structure–activity relationship. Of particular importance are site-selective synthetic methods applicable to functional groups that are abundantly present in natural products with biological activity.^1b,1f^ The hydroxyl moiety is undoubtedly one of the most frequently modified in a site-selective manner, although other functional groups among those frequently encountered in natural products were also addressed in such studies.^2,3^ Site-selective nonenzymatic functionalization of a variety of synthetic and natural di- and polyol compounds were described by a number of research groups.^4−31^ The repertoire of such modifications encompassed simpler diol substrates^9,12,14,21−23,29−31^ along with more elaborate glycoside derivatives^8,10,13−17,20−29^ and culminated with complex polyol natural products, among them erythromycin A, vancomycin, teicoplanin A_2_-2, avermectin B_2a_ and ouabain.^4−7,11,18,25^ While the modifications of di- and polyol substrates included O-substitutions, among them alkylations,^14,15,18,23,25^ arylations,^16,26^ phosphorylations,^5,30^ sulfonylations,^13,14,22,29^ and thiocarbonylations,^6,8,27^ as well as oxidations^17,28^ and formal C-alkylations^31^ (converting primary into secondary alcohols), O-acylations appeared as a flagship of almost every alcohol-modifying, site-selective technique being developed.^4,9−11,13−15,19−22,24,29^

**Scheme 1:**
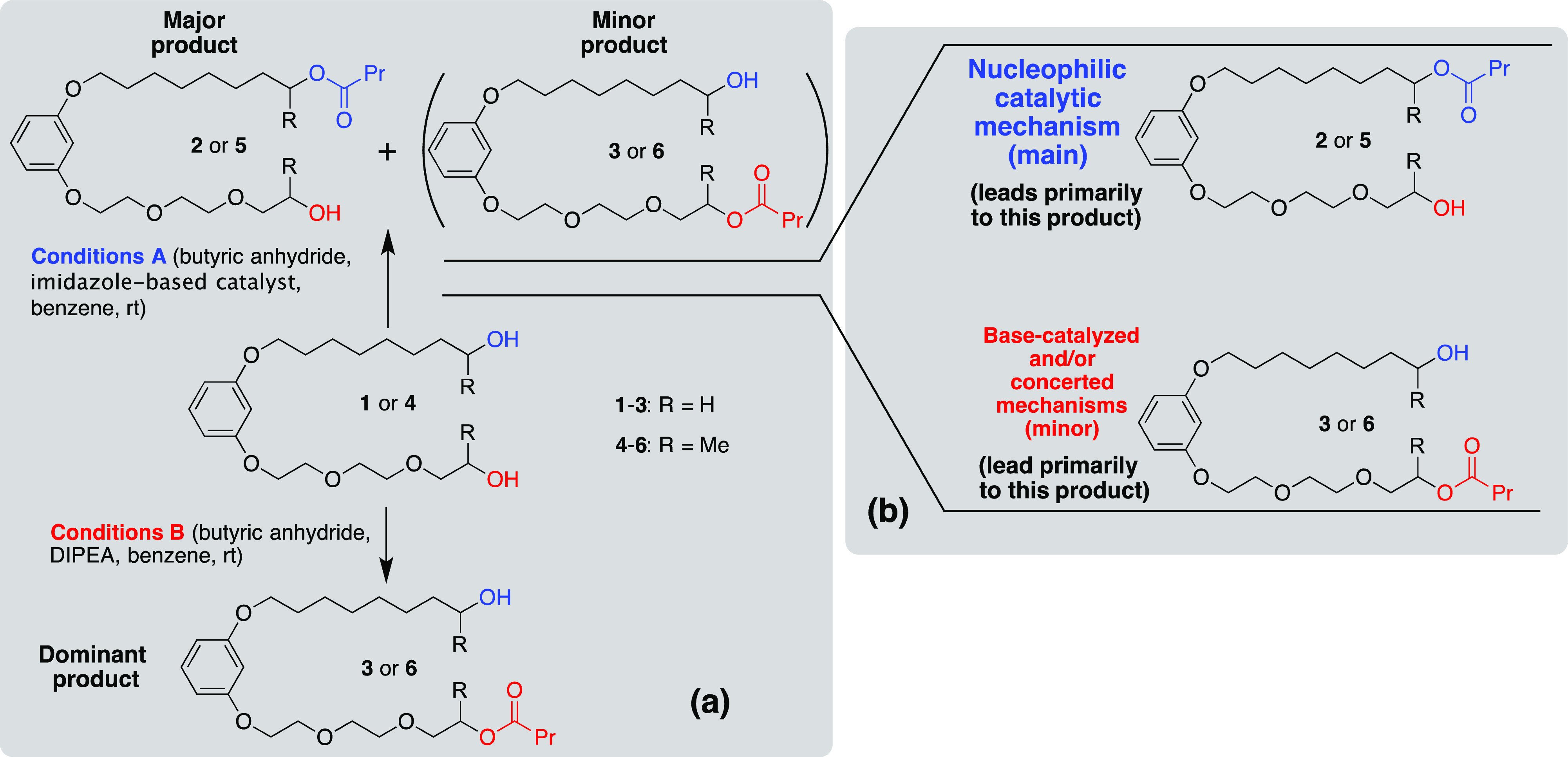
(a) Opposite Selectivity Modes Observed in Acylations of Model Amphiphilic Diols and (b) Proposed Reason for the Erosion of the Apolar-Site-Favoring Selectivity Due to Alternative Reaction Mechanisms

With our particular interest in the area of site-selective reactions being focused on amphiphilic substrates, we reported recently the opposite selectivity modes being induced by a tertiary amine versus imidazole-based dendron-like nucleophilic organocatalysts in acylation of model amphiphilic diols (**1** or **4**, Scheme 1a).^32^ The divergent selectivities were attributed to different mechanisms of the reaction predominantly operating in each case. The use of the N,N-diisopropylethylamine (DIPEA) tertiary amine base, preferentially directing the acylation to the polar-arm site (products **3** or **6**), presumably by inducing the general- or specific-base catalytic mechanism,^33^ led to outstanding 9:1 to 16:1 ratios between the two monoacylated products in the case of the primary model diol and secondary model diol, respectively.^34^ On the other hand, the best ratios achieved in the reactions promoted by the lead nucleophilic catalyst **G2(C**_**5**_**)**, which preferentially directs the acylation toward the apolar arm site (products **2** or **5**), were 1:2.30 and 1:2.21 only for the two model diols, respectively. We assume that although under the conditions of this catalyst-including reaction, it predominantly proceeds via a nucleophilic mechanism involving a cationic intermediate,^35^ some of the acylation still occurs via the concerted route or even by the base-induced pathway, thus eroding the apolar-site-favoring selectivity (Scheme 1b).

We hypothesized that one way to achieve better selectivity in the catalyzed reaction (in favor of the apolar hydroxyl site) is to increase the nucleophilicity of the catalysts, while another is to increase the reactivity of the acylating agent. Both approaches should channel the reaction to proceed through the catalyzed path with the cationic intermediate even more than before, thus increasing the preference for the acylation at the apolar site. Furthermore, the principles leading to the excellent opposing selectivities, already exposed in our previous publication,^32^ enable, in our opinion, applying the new methodology to a suitable amphiphilic natural diol. Herein, we report the results of our efforts directed at both goals, which proceeded in a convergent manner, culminating in a highly site-selective acylation of the midecamycin macrolide antibiotic, a natural amphiphilic molecule with two secondary alcohol sites.

## Results and Discussion

**Scheme 2:**
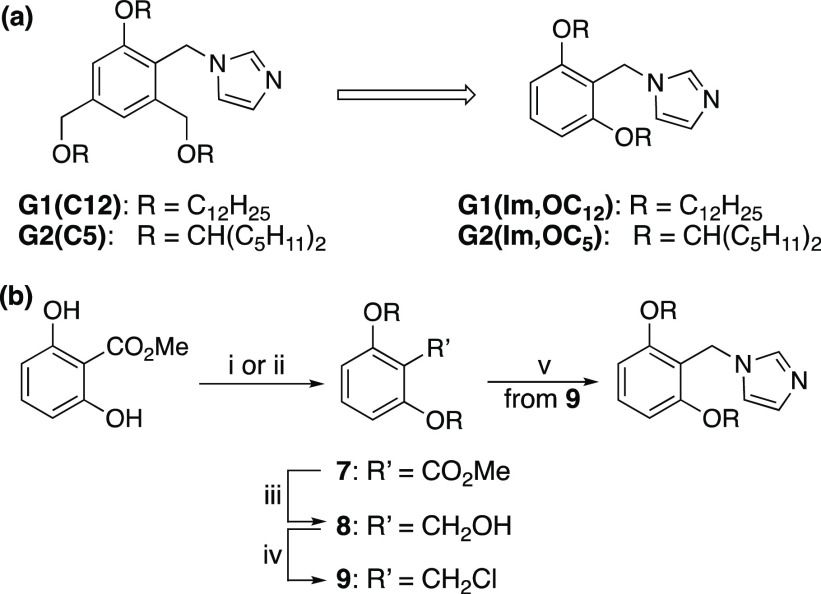
(a) Modification of the Catalyst Design and (b) Synthesis of the Modified Catalysts^37^ Reagents and conditions: (i) RI, K_2_CO_3_, DMF, rt, 90% (for R = C_12_H_25_); (ii) ROH, DIAD, PPh_3_, THF, rt, 78% (for R = CH(C_5_H_11_)_2_); (iii) LiAlH_4_, THF, rt, 87–90%; (iv) SOCl_2_, pyridine, CHCl_3_, rt, 95–97%; (v) imidazole, DMF, 90 °C, 81–82%.

**Scheme 3:**
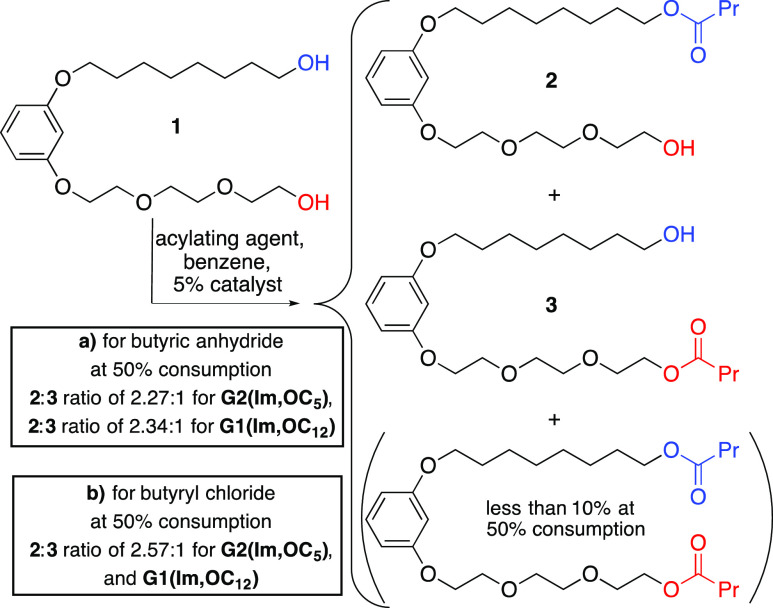
Site-Selective Acylation of the Model Diol with Butyric Anhydride (a) or Butyryl Chloride (b) Promoted by the New Catalysts

As mentioned above, we focused our studies on improving the selectivity favoring the acylation of alcohols at the apolar sites. We hypothesized that slightly changing the design of the catalysts to that recently described for a related reaction,^36^ as depicted in Scheme 2a, will both improve their selectivity due to the increased nucleophilicity (expected because of the replacement of para- and ortho-alkoxyalky substituents on the aromatic ring of benzyl by a properly positioned alkoxy moiety) and simplify their preparation. Two catalysts of the new design, **G1(Im,OC**_**12**_**)** and **G2(Im,OC**_**5**_**)**, were prepared in four steps, as depicted in Scheme 2b,^37^ compared to seven–eight synthetic steps required to prepare the related catalysts reported in the earlier communication and designated as **G1(C12)** and **G2(C5)**.^32^ The butyrylation reaction with the model diol **1** was conducted using the new catalysts, and the results were compared to those obtained using the former catalysts (Scheme 3a). The comparison demonstrates that the new catalysts are somewhat more selective than the former, reaching, respectively, 2.27:1 and 2.34:1 **2**-to-**3** ratios (ca. 70% of monobutyrate **2** in the mixture of the monoacylated products) at 50% consumption. Although this is only a marginal improvement of the selectivity achieved previously, these results validate new catalysts, which are both slightly more selective and more easily attainable, compared to the previously reported leads. Furthermore, the results prove the correctness of the research hypothesis and indicate a possible future research direction.

As mentioned above, we hypothesized that another way to increase the site selectivity of the acylation reaction in favor of the apolar site is increasing the preference of the acylating agent to react via the cationic-intermediate-involving pathway (i.e., the nucleofugality of the leaving group of this agent). Thus, we decided to examine the model reaction with butyryl chloride instead of butyric anhydride since the propensity of acyl halides to react with *N*-alkylimidazoles forming acylimidazolium cations is substantially higher than that of the related carboxylic anhydrides.^38^ In the acylation inflicted by anhydride, carboxylic acid is formed as a byproduct, but in the acylation with acyl halide, a much stronger acid, HCl, is generated instead. While in the case of the anhydride/carboxylic acid, we have already demonstrated that the exclusion of an acid-sequestering base from the catalytic reaction mixture does not substantially affect the acylation rate, we wondered whether the acylations with acid chlorides could be conducted also without such a base. On the other hand, the presence of an acid-sequestering base, for example, DIPEA, will affect the site selectivity in the unwanted direction and can also transform the acylating agent into ketene, thus significantly altering the intended reaction conditions. After a number of preliminary tests, we established that the acylation with butyryl chloride conducted without an acid-sequestering base produces consistent and reproducible results, provided that the quenching of the aliquots taken for monitoring the reaction was performed with a methanolic solution of a base.^39^ The model diol **1** was acylated with butyryl chloride (Scheme 3b) in a background uncatalyzed reaction, as well as in reactions in the presence of a catalytic amount of *N*-benzylimidazole (BnIm), **G1(Im,OC**_**12**_**)**, or **G2(Im,OC**_**5**_**)** (Table 1). The replacement of the anhydride as the acylating agent by the acyl chloride caused a dramatic difference in the reaction rate and in the influence of the catalysts on the rate. While in the case of anhydride, achieving the 50% consumption required 12 h for the uncatalyzed reaction and 1.5–3 h for the catalyzed reactions,^40^ half-life times of only 8–11 min were observed in all experiments with butyryl chloride. Moreover, in the latter case, only minor differences (ca. 20%) were observed in the reaction times required for the 50% consumption as a result of the presence of the potential catalysts in the reaction mixture. A somewhat stronger influence of the catalysts was observed, in these experiments, on the site selectivity. First, in the background reaction, the replacement of anhydride by acyl chloride, as the acylating agent, caused a dramatic difference in selectivity (from the **2**-to-**3** ratio of 1:2.17 to 1.99:1, respectively, at 50% consumption). The use of BnIm shifts the **2**-to-**3** ratio further to 2.18:1, while the use of the new catalysts, **G1(Im,OC**_**12**_**)** or **G2(Im,OC**_**5**_**)**, alters the selectivity even more in favor of the acylation at the apolar alcohol site, reaching in both cases a 2.57:1 ratio at 50% consumption. Somewhat contrary to our expectations, this ratio was only slightly higher than that achieved with butyric anhydride using these catalysts (2.27:1 and 2.34:1, respectively).

**Table 1:** Acylation of the Model Diol with Butyryl Chloride as Depicted in Scheme 3b^a^

entry	catalyst	half-life time (min)	3:2 ratio
**1**		11.1	1:1.99
**2**	BnIm	9.2	1:2.18
**3**	**G1(Im,OC**_**12**_**)**	8.9	1:2.57
**4**	**G2(Im,OC**_**5**_**)**	9.2	1:2.57

a Reaction conditions: 0.1 mmol of the substrate, 0.4 mmol butyryl chloride, and 0.005 mmol (5 mol %) of the catalyst (when used) in 1 mL of benzene at room temperature. The reactions were followed by HPLC, and the **3**:**2** ratio was interpolated for 50% consumption.

In spite of a very fast background reaction, the influence of the catalyst on the rate and selectivity is notable and presumably hints at parallel catalytic pathways (direct and stepwise, via the acylimidazolium catalytic intermediate). Thus, we assume that the protonation of the imidazole core of the catalyst by hydrogen chloride, gradually generated as the reaction proceeds, does not entirely shut the catalysis. The protonation equilibrium of the imidazole catalyst is coupled, under reaction conditions, to its acylation equilibrium. Taking into account the balance between imidazole basicity and nucleophilicity, as well as the excess of the acylating agent in the system, these coupled equilibriums are likely to provide a sufficient concentration of the acylimidazolium species, thus enabling the catalytic cycle even in the presence of HCl.

**Figure 1:**
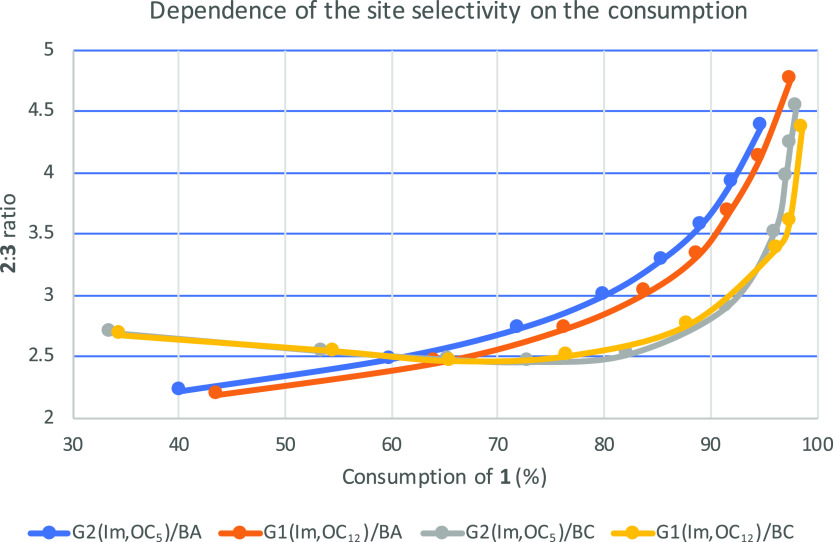
Dependence of the selectivity on the consumption for acylation of **1** with butyric anhydride (BA) or butyryl chloride (BC) catalyzed by the new catalysts.

It is noteworthy that in the case of acyl chloride, an anomalous behavior (comparing to that with the anhydride) was observed when the **2**-to-**3** ratio was plotted against the substrate consumption in the reaction (Figure 1). While in the case of the anhydride, the ratio gradually increases (blue and orange trend lines), in the case of the chloride, it initially decreases and only at ca. 70% consumption starts increasing, first mildly and then steeply (gray and yellow trend lines). The behavior in the first case is an expected one: since under the reaction conditions, acylation of the apolar site is preferred, **3** is formed slower but consumed faster than **2**. Hence, the **2**-to-**3** ratio increased as the reaction progresses. We do not have at the moment an explanation for the trend observed in the reaction with butyryl chloride, as depicted in Figure 1; however, it is not related to the imidazole-affecting equilibriums discussed above since this trend was also observed for the uncatalyzed reaction. In spite of the mentioned shortcomings, the reaction with butyryl chloride in the presence of one of the new catalysts exhibits the highest **2**-favoring selectivities, combined with very short reaction times, and as such is worth being included in the arsenal of site selectivity-inducing techniques for amphiphilic diol acylation.

**Figure 2:**
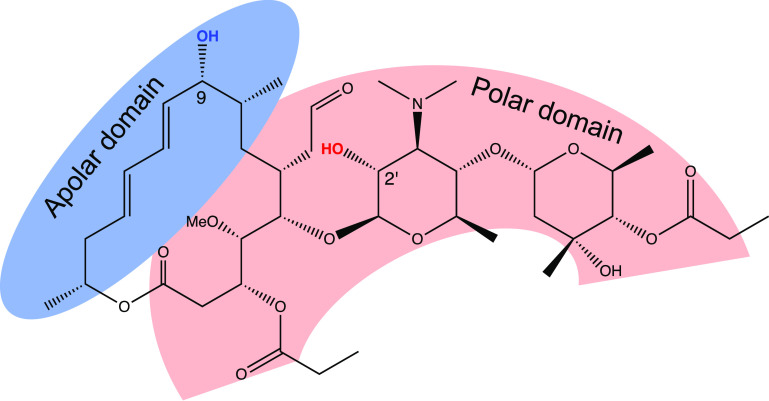
Midecamycin A_1_.

Capitalizing on the outcome of the abovementioned experiments as well as the preceding studies with model diol amphiphiles,^32,41^ we proceeded to implement the new selectivity-inducing techniques on midecamycin A_1_, an antibiotic related to the leucomycin family.^42^ This member of the macrolide class of natural products^43^ seemed to us particularly suitable for demonstrating the extension of the selectivity-inducing acylation reaction conditions, deduced from the model diol studies, to natural polyol compounds. Midecamycin A_1_ possesses an amphiphilic structure with approximately half of the macrocycle constituting its apolar region and the aminodisaccharide appendage, together with another part of the macrocycle, being of a substantially more polar character (Figure 2). Each of the domains (both apolar and polar) harbors a single secondary alcohol functionality[(at C(9) and C(2′), respectively].^44^ Furthermore, two acetal oxygens and the amino nitrogen of the mycaminose sugar, bearing the secondary alcohol in the polar domain, are β-positioned relative to its hydroxyl and, thus, are capable of generating hydrogen bonding, similar to the situation in the model diol. A computational study of a related leucomycin family antibiotic revealed that all its energetically feasible conformations, including the most probable one, incorporate a hydrogen bond between the C(2′) alcohol hydrogen and the nearby C(1′)-bound exocyclic acetal oxygen.^45^

**Figure 3:**
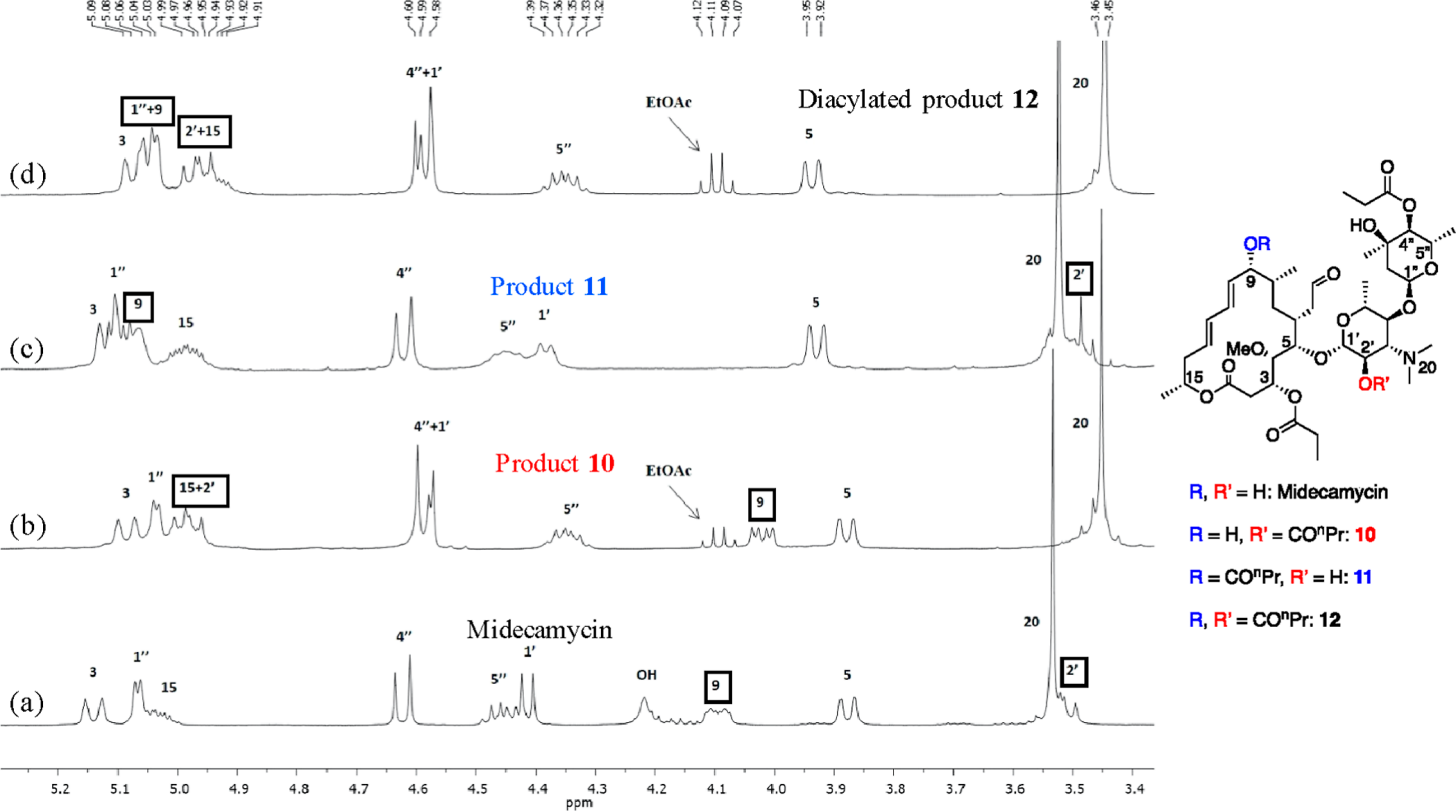
Less-congested region of the ^1^H NMR spectra of the substrate and the products in the butyrylation of midecamycin A_1_: (a) midecamycin; (b) product **10**, monoacylated in the polar region; (c) product **11**, monoacylated in the apolar region; and (d) diacylated product **12**.

**Figure 4:**
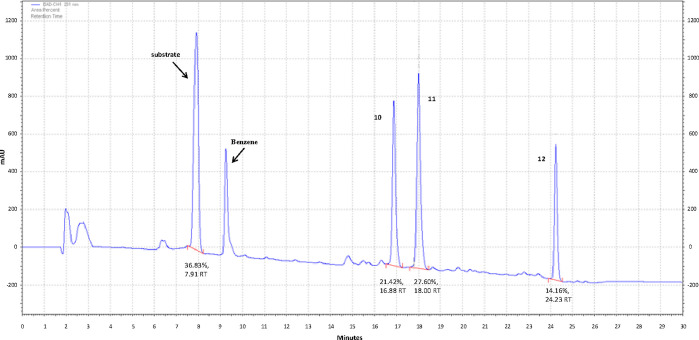
Typical HPLC plot for butyrylation of midecamycin A_1_ (substrate). Chromatography conditions: Apollo C18 5 μm column, acetonitrile–aqueous ammonium formate solution (52:48), 1.5 mL/min, 235 nm. Retention times: 7.9 min (midecamycin), 16.9 min (**10**), 18.2 min (**11**), and 24.2 min (**12**).

**Scheme 4:**
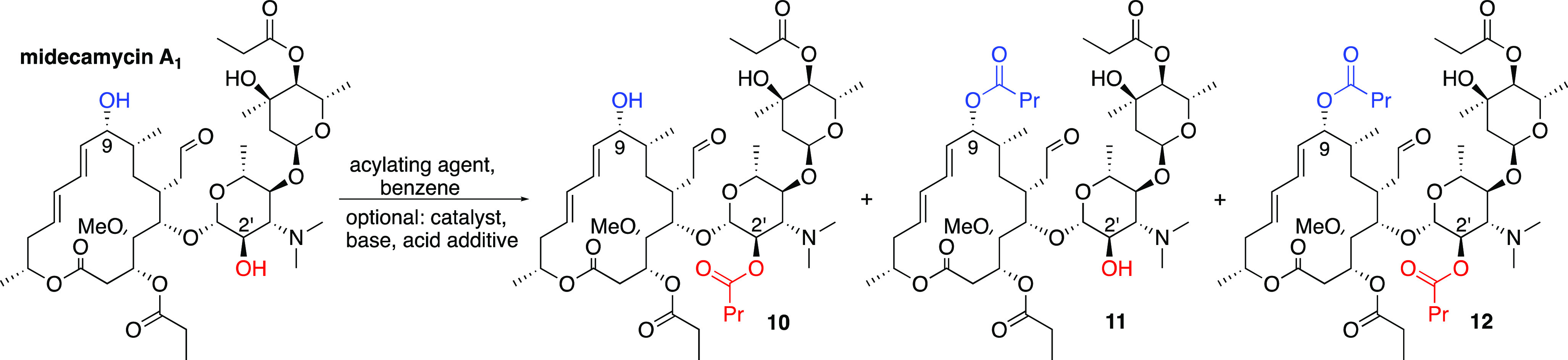
Acylation of Midecamycin A_1_

Although midecamycin displays a complex ^1^H NMR spectrum, the previously reported peak assignment,^46^ as well as the changes observed in the less overlap-impaired region in the spectra of crude reaction mixtures (3.4–5.2 ppm, see Figure 3), combined with the high-performance liquid chromatography (HPLC) analysis of these mixtures (Figure 4), helps unveil the course of the events following subjection of the macrolide substrate to various acylating conditions. The characteristic change observed by HPLC upon monoacylation of midecamycin on the hydroxyl of the polar region (the “polar monoacylated product” **10**, Scheme 4) was the increase in the retention time from 7.9 to 16.9 min under the chosen separation conditions (see the Experimental Section). In addition, the comparison of the ^1^H NMR spectrum of **10** to that of midecamycin exhibited a shift from 3.51 to 4.96 ppm for the signal of the C(2′) proton and a shift from 4.40 to 4.57 ppm for the signal of the C(1′) proton (Figure 3b vs 3a).^47,48^ In the case of monoacylation of midecamycin on the hydroxyl of the apolar region (the “apolar monoacylated product” **11**, Scheme 4), the HPLC retention time was increased to 18.2 min, while in the ^1^H NMR spectrum, a characteristic shift from 4.08 to 5.05 ppm was observed for the C(9) proton (Figure 3c vs 3a).^47,48^ Finally, the retention time of the 9,2′-dibutyrylated midecamycin **12** was 24.2 min, while in the ^1^H NMR spectrum, the signals of its C(9), C(2′), and C(1′) protons were shifted to 5.05, 4.95, and 4.58 ppm, respectively, from their abovementioned positions in the spectrum of midecamycin (Figure 3d vs 3a).^47,48^ Noteworthily, the abovementioned changes were hypothesized during the analysis of the initial crude reaction mixtures and confirmed for pure compounds **10**–**12**, isolated at the more advanced stages of the study.

The initial experiments provided the following construal. In the background reaction experiment, midecamycin produces only two of the products, **10** and **12**, without any visible amount of **11** in the chromatograms of the reaction mixture, upon being reacted with butyric anhydride without any base or catalyst additive (Table 2, entries 1,2). While after 6 h, mostly the starting material and product **10** monoacylated at the C(2′) position were observed, after 24 h, the amount of the diacylated product increased substantially and the starting material was almost completely consumed, although **10** still constituted the major component of the mixture. This reaction monitoring means that **10** is a predominant product of the first acylation, and **11**, if formed at all, is consumed so rapidly during the second acylation that it cannot be detected by HPLC (or NMR). Repeating the experiment in the presence of a stoichiometric amount of DIPEA (Table 2, entries 3 and 4) did not change the situation, nor did it substantially enhance the reaction rate. However, adding 5 mol % of the BnIm catalyst to the reaction (without the DIPEA base) accelerated the reaction by many orders of magnitude (Table 2, entries 5–7). More than 90% of the substrate was consumed in just 15 min, while above 99% was consumed in 24 h. Nevertheless, also under these conditions, mainly two products, **10** and **12**, were observed for all reaction times. Only traces of **11** (up to 1%) were sometimes detectable. Thus, also in this case, **10** is a predominant product of the first acylation, and **11**, though probably formed to some minor extent, is consumed rapidly during the second acylation.

**Table 2:** Acylation of Midecamycin A_1_ with Butyric Anhydride According to Scheme 4^a^

entry	catalyst	additive	reaction time (h)	consumption (%)	10 (%)	11 (%)	12 (%)	10:11 ratio^d^
1			6	62	60	0	2	nd
2			24	98	54	0	44	nd
3		DIPEA^b^	1	30	30	0	0	nd
4		DIPEA^b^	3	44	44	0	0	nd
5	BnIm		0.25	92	91	<1	1	>91:1
6	BnIm		1	93	83	<1	10	>83:1
7	BnIm		24	99	29	1	69	29:1
8	BnIm	*p*-TsOH^c^	1	9	6	3	0	2.0:1
9	BnIm	*p*-TsOH^c^	7	27	16	9	2	1.8:1
10	BnIm	*p*-TsOH^c^	15	35	20	12	3	1.7:1
11	BnIm	*p*-TsOH^c^	22	40	22	14	4	1.6:1
12	**G2(Im,OC**_**5**_**)**	*p*-TsOH^c^	15	41	18	19	4	1:1.1
13	**G2(Im,OC**_**5**_**)**	*p*-TsOH^c^	22	46	19	21	6	1:1.1
14	**G2(Im,OC**_**5**_**)**	*p*-TsOH^c^	70	63	21	28	14	1:1.3

a Reaction conditions: 0.1 mmol of the substrate, 0.4 mmol butyric anhydride, the additive (when used), and 0.005 mmol (5 mol %) of the catalyst (when used) in 1 mL of benzene at room temperature. The consumption and yields were determined by HPLC.

b 0.3 mmol.

c 0.1 mmol.

d nd = not determined, since product **11** was not detected.

The similar selectivity pattern in all three experiments (the background reaction, the reaction in the presence of the base, and the reaction in the presence of the nucleophilic catalyst) leading overwhelmingly to the C(2′) alcohol acylation, as well as the lack of the reaction acceleration by the external DIPEA base, is apparently explained by the “internal base”-induced reaction taking place only at the site in the proximity of the basic C(3′)-bound dimethylamino group. This group activates intramolecularly the nearby β-positioned hydroxyl nucleophile in all three cases and masks any rate-affecting external DIPEA base influence, prone to be significantly weaker than that of the substrate-decorating amino group itself. The huge acceleration of the reaction in the presence of the BnIm is likely explained by the concurrent activation of the electrophile by the catalyst.

If the above explanation is correct, then the way to override the substrate-controlled selectivity is by neutralizing the influence of the basic dimethylamino group in the substrate, for example, by its protonation. Seeing as butyric acid, being formed in the acylation reaction as the byproduct, did not affect the selectivity in the looked-for manner in the abovementioned experiments without the external base, we hypothesized that its acidity is not strong enough to produce the desired effect. Thus, we decide to use the stronger *p*-toluenesulfonic acid as an additive during the acylation reaction. Addition of one equivalent of the acid to the reaction catalyzed by BnIm had indeed a dramatic effect on site selectivity, though it considerably slowed down the reaction rate (Table 2, entries 8–11). For the first time in this study, a significant amount of the C(9)-monobutyrate product **11** was observed by HPLC and NMR. While the ratio between the two monoacylated products was still in favor of **10** (extrapolated to be 1.4:1 at 50% midecamycin consumption), this was a major alteration of the complete substrate-dictated dominance of **10** in the parallel experiment without the acid additive (entries 6–9). Applying the new **G2(Im,OC**_**5**_**)** catalyst, which has a stronger predisposition (compared to BnIm) to induce the acylation at the apolar sites (as described above for the model substrate study), further improved the selectivity in favor of **11**, while concomitantly slightly increasing the reaction rate over that observed in the reaction with BnIm (Table 2, entries 12–14). For the first time in this series of experiments, **11** became the dominant monoacylation product with the interpolated 1.2:1 **11**-to-**10** ratio at 50% consumption (at ca. 27 h).

Seeking to further improve selectivity in favor of the reaction at the apolar alcohol site, as well as the reaction times, we changed the acylating agent to butyryl chloride, another tool that, as described above, produces such an outcome in the model substrate acylation. Besides, the byproduct of alcohol acylation with acyl chlorides is hydrogen chloride, a very strong acid that can protonate the substrate dimethylamino group, thus cancelling its C(2′) hydroxyl-directing influence in this transformation.

Already, the background reaction with butyryl chloride (without a base or a catalyst) displayed a C(9) hydroxyl-favoring acylation with a 3.0:1 ratio between monoacylated products **11** and **10** at 50% consumption after ca. 1.5 h (extrapolated from Table 3, entries 1–2). Adding a catalyst to the reaction mixture further facilitated the reaction [Table 3, entries 3–8, half-life times of ca. 0.9 h for both BnIm and **G2(Im,OC**_**5**_**)**] and dramatically augmented the site selectivity, reaching the outstanding 11.0:1 ratio between the two monobutyrylated products [interpolated from Table 3, entries 6–7 for the **G2(Im,OC**_**5**_**)** catalyst]. Thus, the combination of butyryl chloride, as the acylating agent, with the catalyst, which displayed the best performance selectivity-wise in the model studies, led to the most remarkable overturn of the reaction site selectivity, with the total preference for **10** being replaced by the almost total preference for **11** as the product of the first acylation. Noteworthily, the influence of the catalyst presence and its exact structure on the consumption rate is experienced not only in the beginning but also during the advanced stage of the reaction, as evident from the data in Table 3.

**Table 3:** Acylation of Midecamycin A_1_ with Butyryl Chloride According to Scheme 4^a^

entry	catalyst	reaction time (h)	consumption (%)	10 (%)	11 (%)	12 (%)	10:11 ratio
1		1	34	11	20	3	1:1.8
2		1.5	48	11	32	6	1:2.9
3	BnIm	0.5	28	5	21	1	1:4.2
4	BnIm	1	57	6	47	4	1:7.8
5	BnIm	1.5	68	3	48	17	1:16.0
6	**G2(Im,OC**_**5**_**)**	0.5	33	4	28	2	1:7.0
7	**G2(Im,OC**_**5**_**)**	1	55	4	46	5	1:11.5
8	**G2(Im,OC**_**5**_**)**	1.5	79	3	54	22	1:18.0

a Reaction conditions: 0.1 mmol of the substrate, 0.4 mmol butyryl chloride, and 0.005 mmol (5 mol %) of the catalyst (when used) in 1 mL of benzene at room temperature. The consumption and yields were determined by HPLC.

It is worth mentioning that analogues of **11**, that is, midecamycin A_1_ or its 3″-OMe derivative monoacylated at the C(9) alcohol site, were obtained in the past as the main products by direct acylation with acyl halides.^47c,49^ This procedure, however, required a large excess of pyridine being used in the reaction, and the reports did not include the site selectivity data. Alternatively, **11** can be obtained from midecamycin in a two-stage sequence via diacylation product **12**, which undergoes methanolysis of the C(2″) ester upon incubation in methanol at ambient or slightly elevated temperature, as was demonstrated for closely related analogues.^47c,50^ Nevertheless, our work demonstrates, for the first time, catalyst-effected site selectivity enhancement in the acylation of midecamycin, with the catalyst control approach and catalyst design being responsible for increasing the site selectivity from the 3:1 ratio of the monoacylated products in the uncatalyzed reaction to a remarkable 11:1 (**11**-to-**10** ratio).

The influence of the acylating agent change, from anhydride to acyl chloride, on the site selectivity is much stronger in the reaction of the midecamycin substrate than in that of the model diol. On the other hand, this change strongly affects the reaction rate in both cases, but with the opposite outcome (enhancing it in the case of the model substrate and reducing it in the case of midecamycin). These phenomena are most likely explained by the presence of an internal basic group in the proximity of one of the alcohols in midecamycin and its protonation by HCl in the case of acyl chloride (as we suggested above). The dramatic acylation agent-dependent influence of the base nature, as well as its mere presence, on the rate and selectivity of acylation was documented in the study of DMAP-catalyzed acylation of alcohols by Albert and Kattnig^51^ However, the control of the reaction by the counter-anion of the cationic intermediate and the carboxylate-dictated cyclic transition states, implicated by the authors as the rationale for the observed effects, are less likely to play a significant role in our case since both substrates lack the 1,2-, 1,3-, or 1,4-diol pattern and the experiments were carried out without an auxiliary base. Indeed, the influence of the acylating agent change on the reaction rate in our study did not match that in the abovementioned report.

In conclusion, we demonstrated that, in the case of both a simple amphiphilic diol model substrate and a natural product amphiphile, the change from a carboxylic anhydride to an acyl chloride acylating agent causes a major shift of the site selectivity toward the reaction of the alcohol in the apolar domain. We have shown that the application of the nucleophilic catalysts reinforces this selectivity preference for both acylating agents. Furthermore, we confirmed that the catalyst design plays an important role in achieving even higher selectivity. We observed for the natural product substrate, on the other hand, that a basic group in the substrate counteracts this influence of the acylating agent and the catalyst, directing the acylation toward the alcohol in the polar domain, similar to the recently revealed influence of an external base. This study, combined with our previous findings,^32,41^ reveals that by choosing the proper combination of an acylating agent, a base, and a catalyst, the acylation of alcohols in amphiphiles could be directed to either of the substrate domains with good to excellent site selectivity ratios.

## Experimental Section

### General Information

All reactions, requiring anhydrous conditions, were conducted under an atmosphere of nitrogen in oven-dried glassware in dry solvents. Dry benzene and dimethylformamide (DMF) were purchased at the highest available purity and used as received. Tetrahydrofuran (THF) was dried and distilled over sodium metal with benzophenone as the indicator. Chloroform, ethyl acetate, hexanes, and methanol, as well as HPLC grade water, methanol, and acetonitrile, were purchased and used as received. All reagents were purchased at the highest available purity and used as received.

Thin-layer chromatography (TLC) was performed on silica gel plates Merck 60 F_254_, and the compounds were visualized by irradiation with UV light or by KMnO_4_. Flash column chromatography was carried out using flash-grade silica gel (particle size 0.040–0.063 mm); the eluent is given in parentheses.

^1^H NMR (400 MHz) and ^13^C NMR (100 MHz) spectra were recorded on Bruker AVANCE-400 spectrometers in CDCl_3_ with residual CHCl_3_ (^1^H, 7.26 ppm) or CDCl_3_ (^13^C, 77.16 ppm) as an internal standard.

Mass spectroscopy (MS) analyses were conducted on a Waters SYNAPT instrument (ESI, APCI, or APPI ionization methods and the TOF detection method).

HPLC experiments were carried out using an Apollo C18 5u column on a Hitachi Elite LaChrome instrument, equipped with a diode array UV/vis detector, with acetonitrile and water or aqueous ammonium formate as the eluting solvents.

The synthesis of **G1(Im,OC**_**12**_**)** followed the recently disclosed route.^36^

### Synthesis of **G2(Im,OC**_**5**_**)**

#### Methyl 2,6-Bis(undecan-6-yloxy)benzoate (**7**)

To a stirred solution of methyl 2,6-dihydroxybenzoate (1.82 g, 10.8 mmol, 1.0 equiv) in dry THF (60 mL), 6-undecanol (7.44 g, 43.2 mmol, 4.0 equiv) and triphenylphosphine (8.50 g, 32.4 mmol, 3.0 equiv) were added. Then, the mixture was cooled with an ice bath, and a solution of DIAD (6.38 mL, 3.0 mmol, 3.0 equiv) in dry THF (20 mL) was added dropwise. The reaction was allowed to warm to room temperature and was stirred overnight. Then, the solvent was evaporated, and purification of the crude by flash column chromatography (from pure hexanes to 1:1 EtOAc/hexanes) afforded the pure product as a colorless oil. Yield 4.20 g (78%).

^1^H NMR (400 MHz, CDCl_3_): δ 7.17 (d, *J* = 8.5 Hz, 1H); 6.45 (t, *J* = 8.5 Hz, 2H); 4.19 (quin, *J* = 5.8 Hz, 2H); 3.84 (s, 3H); 1.62 (m, 8H); 1.50–1.20 (m, 24H); 0.88 (t, *J* = 7.0 Hz, 12H). ^13^C{^1^H} NMR (100.8 MHz, CDCl_3_): δ 167.4, 156.7, 130.5, 115.6, 105.6, 79.0, 51.9, 33.9, 32.1, 25.0, 22.7, 14.2. MS (AP-TOF) *m*/*z*: 445.4 ([M − OMe]^+^). HRMS (AP-TOF) *m*/*z*: calcd for C_30_H_52_O_4_Na ([M + Na]^+^), 499.3763; found, 499.3773.

#### (2,6-Bis(undecan-6-yloxy)phenyl)methanol (**8**)

To a cooled (ice bath) solution of lithium aluminum hydride (20 mL, 2.4 equiv, 1 M solution in THF) in 20 mL of dry THF was slowly added methyl 2,6-bis(undecan-6-yloxy)benzoate (4.00 g, 8.4 mmol, 1.0 equiv) dissolved in 20 mL of dry THF. The reaction was allowed to warm to room temperature and stirred for 6 h. The mixture was then cooled in an ice bath, and the reaction was quenched by the dropwise addition of water, followed by 15% sodium hydroxide and water (*n* g of lithium aluminum hydride requires *n* mL of water, *n* mL of 15% sodium hydroxide, and 3*n* mL of water, added in succession). After vigorous stirring for another 30 min, the mixture was filtered with suction; the granular precipitate was washed thoroughly with THF. The organic solution was evaporated and purified by flash column chromatography (from pure hexanes to 1:1 EtOAc/hexanes) to afford the pure product as a colorless oil. Yield: 3.40 g (90%).

^1^H NMR (400 MHz, CDCl_3_): δ 7.12 (t, *J* = 8.4 Hz, 1H); 6.48 (d, *J* = 8.4 Hz, 2H); 4.79 (d, *J* = 6.2 Hz, 2H); 4.27 (quin, *J* = 5.8 Hz, 2H); 2.76 (t, *J* = 6.5 Hz, 1H); 1.68 (m, 8H); 1.46–1.24 (m, 24H); 0.88 (t, *J* = 7.0 Hz, 12H). ^13^C{^1^H} NMR (100.8 MHz, CDCl_3_): δ 157.5, 128.5, 119.1, 105.4, 78.3, 55.7, 33.8, 31.9, 25.1, 22.6, 14.0. MS (ES-TOF) *m*/*z*: 471.4 ([M + Na]^+^), 919.8 ([2 M + Na]^+^). HRMS (ES-TOF) *m*/*z*: calcd for C_29_H_52_O_3_Na ([M + Na]^+^), 471.3814; found, 471.3808.

#### 2-(Chloromethyl)-1,3-bis(undecan-6-yloxy)benzene (**9**)

SOCl_2_ (0.65 mL, 8.9 mmol, 2.0 equiv) was added dropwise to a solution of (2,6-bis(undecan-6-yloxy)phenyl)methanol (2.00 g, 4.5 mmol, 1.0 equiv) and pyridine (0.72 mL, 8.9 mmol, 2.0 equiv) in CHCl_3_ (20 mL). Following the completion of the addition, the reaction mixture was stirred in a water bath at room temperature for 10 min. The mixture was then poured into a separation funnel, which contained 2 N HCl (3.54 mL), 7.00 g of ice, and hexanes (50 mL), and the layers were separated. The organic phase was washed with saturated aq. NaHCO_3_ solution (1.67 mL), dried over MgSO_4_, and concentrated under reduced pressure to give the product as a colorless solid. The product was used in the next step without further purification. Yield: 2.00 g (95%).

^1^H NMR (400 MHz, CDCl_3_): δ 7.16 (t, *J* = 8.4 Hz, 1H); 6.45 (d, *J* = 8.4 Hz, 2H); 4.78 (s, 2H); 4.29 (quin, *J* = 5.8 Hz, 2H); 1.70–1.63 (m, 8H); 1.47–1.28 (m, 24H); 0.88 (t, *J* = 7.0 Hz, 12H). ^13^C{^1^H} NMR (100.8 MHz, CDCl_3_): δ 157.8, 129.7, 115.6, 104.5, 77.8, 36.1, 33.8, 31.9, 24.9, 22.6, 14.0. MS (AP-TOF) *m*/*z*: 467.0 ([M + H]^+^). HRMS (AP-TOF) *m*/*z*: calcd for C_29_H_52_O_2_Cl ([M + H]^+^), 467.3656; found, 467.3655.

#### 1-(2,6-Bis(undecan-6-yloxy)benzyl)-1*H*-imidazole [G2(Im,OC_5_)]

To a stirred solution of 2-(chloromethyl)-1,3-bis(undecan-6-yloxy)benzene (2.00 g, 4.3 mmol, 1.0 equiv) in DMF (50 mL), imidazole (1.46 g, 21.4 mmol, 5.0 equiv) was added. The mixture was stirred at 85 °C for 17 h, and the progress of the reaction was monitored by TLC. After the reaction completion, the mixture was diluted with H_2_O (20 mL) and extracted with ethyl acetate (3× 30 mL). The combined extracts were washed with H_2_O (6× 30 mL), NaOH 1 N (30 mL), and brine (30 mL), dried over MgSO_4_, and concentrated under reduced pressure. Purification by flash column chromatography (from pure hexanes to 1:1 EtOAc/hexanes) afforded the pure product as a colorless oil. Yield: 1.75 g (82%).

^1^H NMR (400 MHz, CDCl_3_): δ 7.55 (s, 1H); 7.14 (t, *J* = 8.4 Hz, 1H); 6.97 (s, 1H); 6.92 (s, 1H); 6.44 (d, *J* = 8.4 Hz, 2H); 5.11 (s, 2H); 4.29 (quin, *J* = 5.8 Hz, 2H); 1.62 (m, 8H); 1.42–1.27 (m, 24H); 0.86 (t, *J* = 7.0 Hz, 12H). ^13^C{^1^H} NMR (100 MHz, CDCl_3_): δ 157.5, 137.6, 129.5, 128.1, 119.4, 113.9, 104.3, 77.6, 39.2, 33.7, 31.8, 25.0, 22.5, 14.0. MS (ES-TOF) *m*/*z*: 499.4 ([M + H]^+^), 997.8 ([2 M + H]^+^). HRMS (ES-TOF) *m*/*z*: calcd for C_32_H_54_O_2_N_2_ ([M]^+^) 498.4185; found, 498.4188.

### General Procedure for the Diol **1** Acylation Catalysis

To the solution of the substrate (**1**, 0.1 mmol, 1 equiv, 2 hydroxyl equiv) in dry benzene (1 mL), the catalyst (5% mol, 0.005 mmol, 0.05 equiv, when the catalyst was used), DIPEA (0.3 mmol, 3 equiv, when the base was used), and the acylating agent (butyric anhydride or butyryl chloride, 0.4 mmol, 4 equiv) were added. The solution was stirred at room temperature. During the reaction, aliquots were taken at constant intervals (30 μL of each sample) and quenched with 0.5 mL of methanol (for anhydride) or methanolic solution of DIPEA (0.03 M, for acyl chloride). Each sample was analyzed using HPLC to determine the ratio of the products and the degree of the conversion.

The typical chromatography conditions for analysis of the model diol butyrylation were reported previously.^32^

### General Procedure for the Midecamycin Acylation Catalysis

To the solution of the substrate (midecamycin, 0.1 mmol, 1 equiv, 2 hydroxyl equiv) in dry benzene (1 mL), the catalyst (5% mol, 0.005 mmol, 0.05 equiv, when the catalyst was used), DIPEA (0.3 mmol, 3 equiv, when the base was used) or *p*-TsOH (0.1 mmol, 1 equiv, when the acid was used), and the acylating agent (butyric anhydride or butyryl chloride, 0.4 mmol, 4 equiv) were added. The solution was stirred at room temperature. At the end of the reaction, it was quenched with methanol (for anhydride) or a methanolic solution of DIPEA (0.03 M, for acyl chloride). Each quenched sample was analyzed using HPLC to determine the ratio of the products and the degree of the conversion. On a few occasions, a sample of the reaction mixture was taken, quenched, and analyzed, while the remaining mixture continued to react.

The typical chromatography conditions for the analysis of midecamycin butyrylation: an Apollo C18 5 μm column, acetonitrile–aqueous ammonium formate solution (52:48), 1.5 mL/min, 235 nm; and retention times: 7.9 min (midecamycin), 16.9 min (**10**), 18.2 min (**11**), and 24.2 min (**12**).

#### **11** (Product of Monobutyrylation at the Apolar Site of Midecamycin)

^1^H NMR (400 MHz, CDCl_3_): δ 9.66 (s, 1H); 6.72 (dd, *J* = 10.5 Hz, *J* = 4.7 Hz, 1H); 6.08 (dd, *J* = 10.8 Hz, *J* = 4.5 Hz, 1H); 5.86 (td, *J* = 7.6 Hz, *J* = 3.8 Hz, 1H); 5.58 (dd, *J* = 10.0 Hz, *J* = 5.2 Hz, 1H); 5.07–5.13 (m, 3H); 4.96–5.03 (m, 1H); 4.62 (d, *J* = 11.2 Hz, 1H); 4.41–4.62 (m, 1H); 4.38 (d, *J* = 7.3 Hz, 1H); 3.92 (d, *J* = 9.5 Hz, 1H); 3.52 (s, 3H); 3.49–3.51 (m, 1H); 3.22–3.28 (m, 3H); 2.60–2.79 (m, 4H); 2.40–2.55 (m, 11H); 2.23–2.33 (m, 3H); 2.12–2.21 (m, 2H); 1.99–2.03 (m, 2H); 1.84 (dd, *J* = 10.7 Hz, *J* = 3.6 Hz, 1H); 1.57–1.68 (m, 2H); 1.43–1.52 (m, 1H); 1.12–1.26 (m, 18H); 0.91–0.97 (m, 7H). ^13^C{^1^H} NMR (100.8 MHz, CDCl_3_): δ 201.3, 174.4, 173.8, 173.0 C, 169.8, 138.0, 134.0, 131.6, 122.3, 104.0, 97.1, 84.8, 77.8, 77.0, 76.1(×2), 73.0, 71.7, 69.4, 69.2, 68.7(×2), 63.5, 62.4, 42.1, 42.0(×2), 41.7, 40.9, 37.4, 36.6, 31.3, 30.3, 28.8, 27.6(×2), 25.3, 20.4, 18.8, 18.5, 17.7, 15.0, 13.6, 9.2, 8.9. MS (ES-TOF) *m*/*z*: 884.5 ([M + H]^+^), 906.5 ([M + Na]^+^), 922.5 ([M + K]^+^). HRMS (ES-TOF) *m*/*z*: calcd for C_45_H_74_NO_16_ ([M + H]^+^), 884.5008; found, 884.5009.

#### **10** (Product of Monobutyrylation at the Polar Site of Midecamycin)

^1^H NMR (400 MHz, CDCl_3_): δ 9.60 (s, 1H); 6.62 (dd, *J* = 10.5 Hz, *J* = 4.7 Hz, 1H); 6.03 (dd, *J* = 10.4 Hz, *J* = 4.5 Hz, 1H); 5.75 (td, *J* = 7.5 Hz, *J* = 3.7 Hz, 1H); 5.57 (dd, *J* = 9.5 Hz, *J* = 5.7 Hz, 1H); 4.96–5.10 (m, 4H); 4.57–4.60 (m, 2H); 4.31–4.38 (m, 1H); 4.02 (dd, *J* = 9.5 Hz, *J* = 4.2 Hz, 1H); 3.87 (d, *J* = 8.0 Hz, 1H); 3.45 (s, 3H); 3.25–3.27 (m, 2H); 3.11 (dd, *J* = 9.3 Hz, *J* = 1.3 Hz, 1H); 2.57–2.81 (m, 5H); 2.39–2.54 (m, 11H); 2.06–2.35 (m, 4H); 1.97 (d, *J* = 14.3 Hz, 1H); 1.79–1.84 (m, 2H); 1.60–1.67 (m, 2H); 1.36–1.44 (m, 1H); 1.08–1.25 (m, 18H); 1.60–1.67 (m, 6H); 0.75–0.87 (m, 1H). ^13^C{^1^H} NMR (100.8 MHz, CDCl_3_): δ 201.3, 174.4, 173.8, 171.5, 169.9, 135.8, 132.7, 132.0, 127.5, 100.5, 96.9, 85.4, 77.0, 75.9, 75.0, 73.3, 72.7, 70.8, 69.4, 69.0, 68.6, 67.7, 63.4, 62.0, 42.4, 41.6(×3), 40.9, 37.0, 36.5, 33.3, 29.9, 28.5, 27.6, 27.5, 25.5, 20.5, 18.7, 18.0, 17.8, 14.5, 13.8, 9.3, 8.9. MS (ES-TOF) *m*/*z*: 884.5 ([M + H]^+^), 906.5 ([M + Na]^+^), 922.5 ([M + K]^+^). HRMS (ES-TOF) *m*/*z*: calcd for C_45_H_74_NO_16_ ([M + H]^+^) 884.5008; found, 884.5015.

#### **12** (Product of Dibutyrylation of Midecamycin)

^1^H NMR (400 MHz, CDCl_3_): δ 9.61 (s, 1H); 6.69 (dd, *J* = 10.4 Hz, *J* = 4.8 Hz, 1H); 6.03 (dd, *J* = 10.8 Hz, *J* = 4.4 Hz, 1H); 5.85 (td, *J* = 7.6 Hz, *J* = 3.8 Hz, 1H); 5.53 (dd, *J* = 10.0 Hz, *J* = 5.2 Hz, 1H); 5.03–5.09 (m, 3H); 4.91–4.97 (m, 2H); 4.58–4.60 (m, 2H); 4.32–4.39 (m, 1H); 3.93 (d, *J* = 9.4 Hz, 1H); 3.45 (s, 3H); 3.25–3.27 (m, 2H); 3.10 (dd, *J* = 8.0 Hz, *J* = 1.4 Hz, 1H); 2.61–2.78 (m, 5H); 2.38–2.53 (m, 12H); 2.09–2.31 (m, 6H); 1.96 (d, *J* = 14.1 Hz, 1H); 1.80 (dd, *J* = 10.4 Hz, *J* = 3.9 Hz, 1H); 1.55–1.69 (m, 4H); 1.40–1.46 (m, 1H); 1.09–1.28 (m, 18H); 0.90–1.00 (m, 9H); 0.77–0.81 (m, 1H). ^13^C{^1^H} NMR (100.8 MHz, CDCl_3_): δ 201.2, 174.4, 173.7, 172.9, 171.5, 169.8, 137.9, 133.9, 131.5, 122.4, 100.5, 96.9, 85.4, 77.03, 76.1, 75.9, 74.9, 72.6, 70.9, 69.4, 69.3, 68.7, 67.7, 63.4, 62.0, 42.0, 41.5(×3), 40.8, 37.3, 36.6, 36.5, 31.3, 29.8, 28.5, 27.5(×2), 25.2, 20.4, 18.7, 18.5, 18.0, 17.8, 14.9, 13.8, 13.6, 9.3, 8.9. MS (ES-TOF) *m*/*z*: 954.5 ([M + H]^+^), 976.5 ([M + Na]^+^). HRMS (ES-TOF) *m*/*z*: calcd for C_49_H_80_NO_17_ ([M + H]^+^), 954.5426; found, 954.5417.

## Supporting Information Available

^1^H NMR, ^13^C{^1^H} NMR, and MS spectra for all new compounds and representative HPLC traces for the catalytic reactions (PDF)
