# Supplementary material for: Controlling the Site Selectivity in Acylations of Amphiphilic Diols: Directing the Reaction toward the Apolar Domain in a Model Diol and the Midecamycin A1 Macrolide Antibiotic
Source: J Org Chem. 2022 Jul 8;87(15):9688–98. doi: 10.1021/acs.joc.2c00745 (PMC9361358; doi:10.1021/acs.joc.2c00745)
Supplement: Supplementary file 1 — jo2c00745_si_001.pdf [file jo2c00745_si_001.pdf]

Supporting Information

Controlling the Site Selectivity in Acylations of Amphiphilic Diols: Directing the Reaction Toward the Apolar Domain in a Model Diol and Midecamycin A_1 Macrolide Antibiotic

Reut Fallek, Natali Ashush, Amit Fallek, Or Fleischer and Moshe Portnoy*

School of Chemistry, Raymond and Beverly Sackler Faculty of Exact Sciences, Tel Aviv University, Tel Aviv 699678, Israel

portnoy@tauex.tau.ac.il

Table of Contents

^{1}H and ^{13}C NMR and Mass Spectra S3

HPLC Traces S14

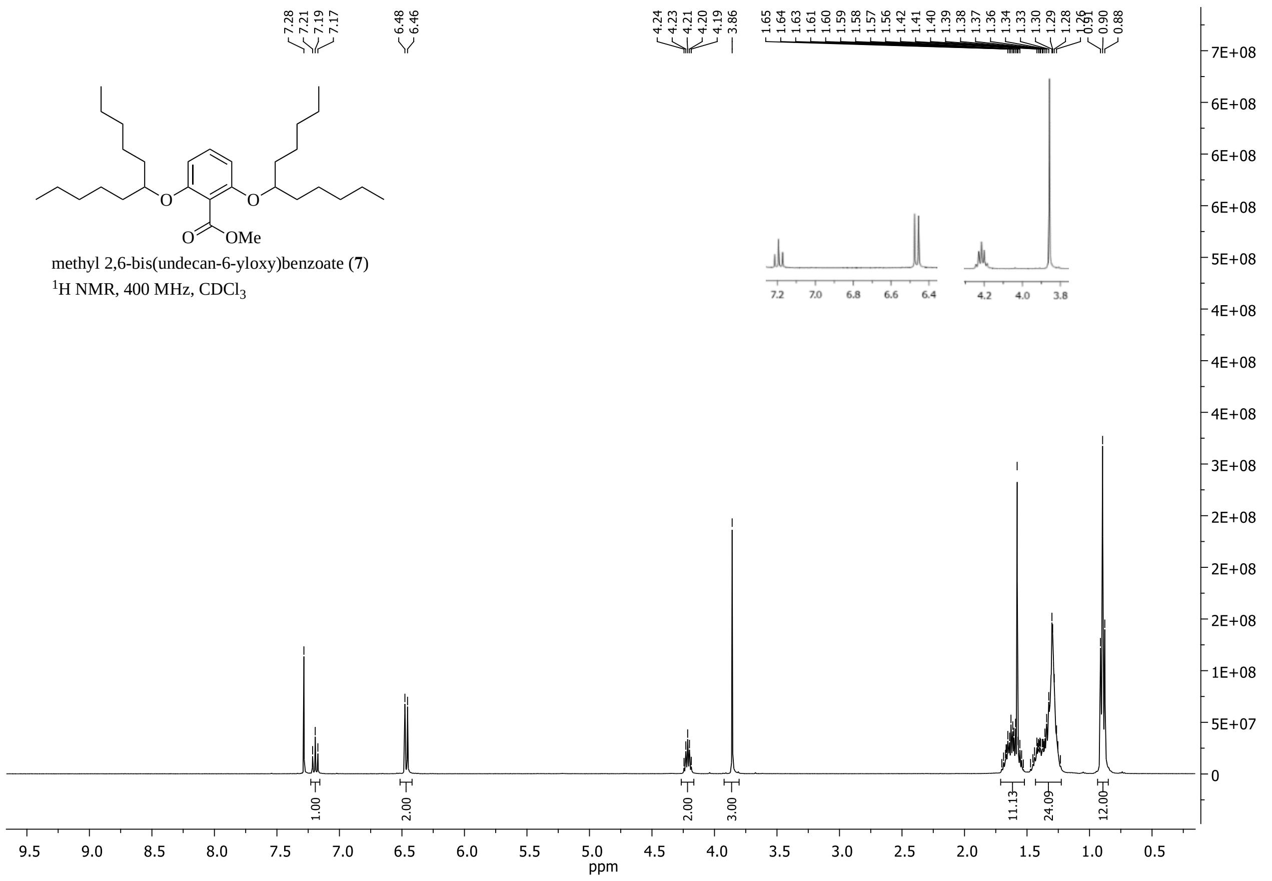
methyl 2,6-bis(undecan-6-yloxy)benzoate (7)
¹H NMR, 400 MHz, CDCl₃
7.28
7.21
7.19
7.17
6.48
6.46
4.24
4.23
4.21
4.20
4.19
3.86
1.65
1.64
1.63
1.61
1.60
1.59
1.58
1.57
1.56
1.42
1.41
1.40
1.39
1.38
1.37
1.36
1.34
1.33
1.30
1.29
1.28
0.91
0.90
0.88
1.00
2.00
2.00
3.00
11.13
24.09
12.00
ppm

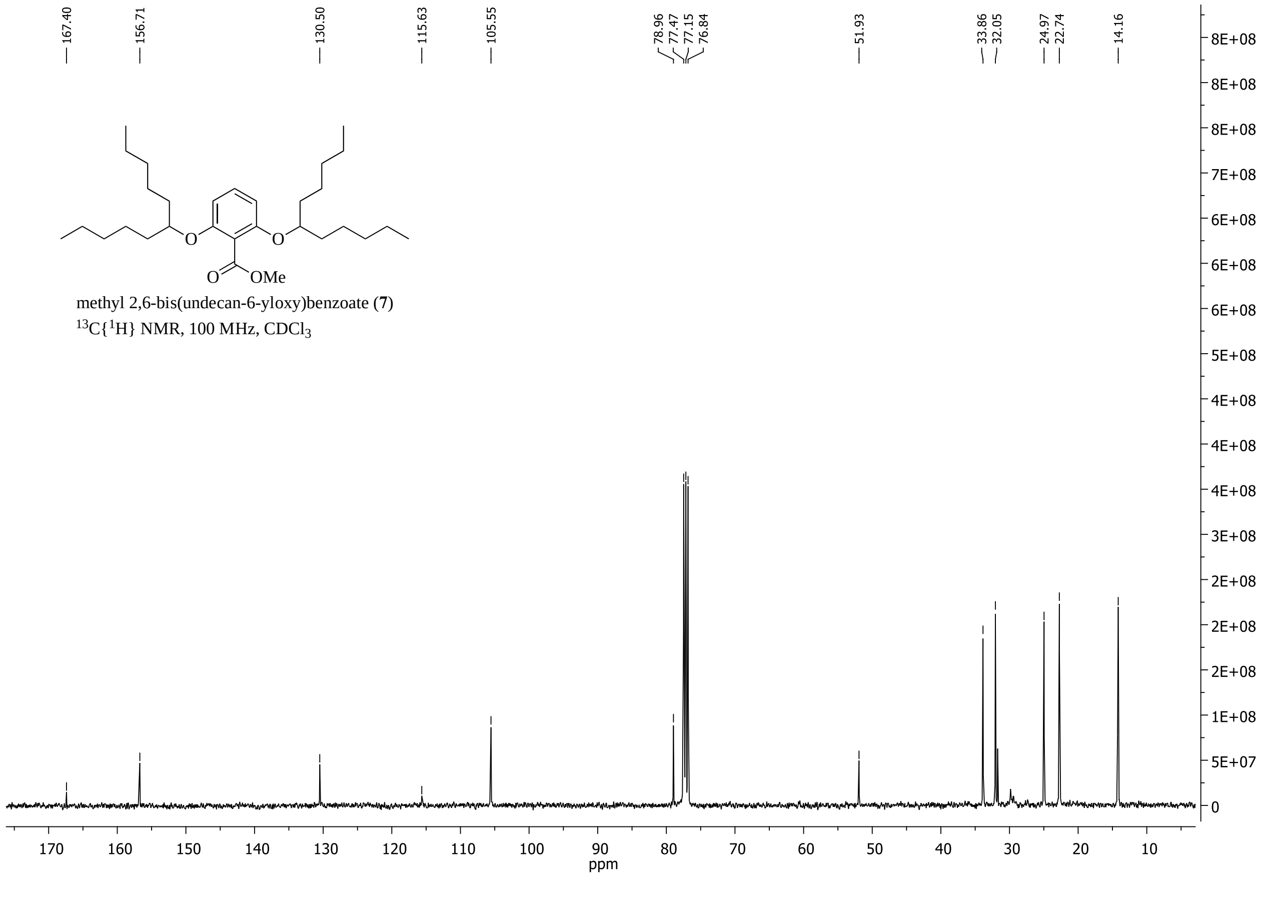
methyl 2,6-bis(undecan-6-yloxy)benzoate (7)
¹³C{¹H} NMR, 100 MHz, CDCl₃
167.40
156.71
130.50
115.63
105.55
78.96
77.47
77.15
76.84
51.93
33.86
32.05
24.97
22.74
14.16
ppm

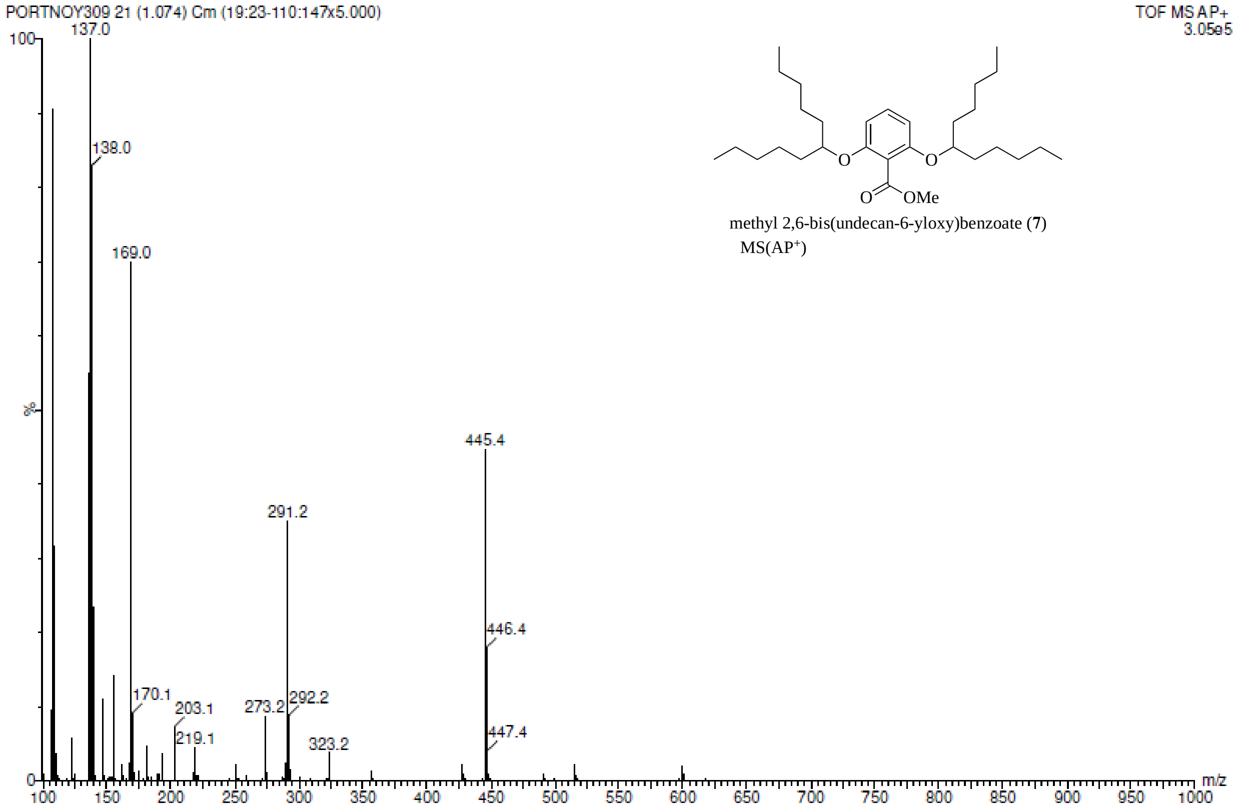
PORTNOY309 21 (1.074) Cm (19:23-110:147x5.000)
TOF MS AP+
3.05e5
137.0
138.0
169.0
170.1
203.1
219.1
273.2
291.2
292.2
323.2
445.4
446.4
447.4
m/z
%
OMe
O
methyl 2,6-bis(undecan-6-yloxy)benzoate (7)
MS(AP+)

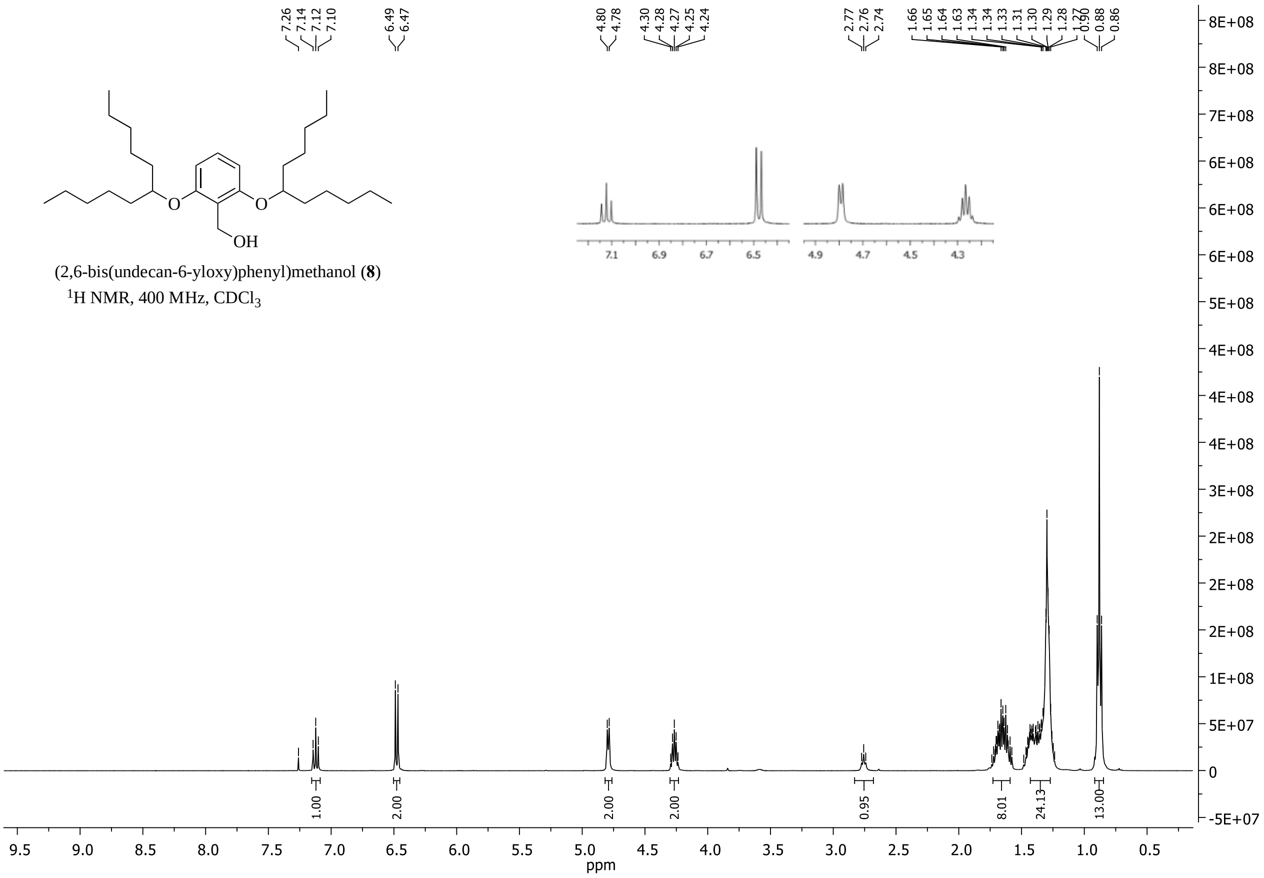
OH
O
(2,6-bis(undecan-6-yloxy)phenyl)methanol (8)
1H NMR, 400 MHz, CDCl3
ppm

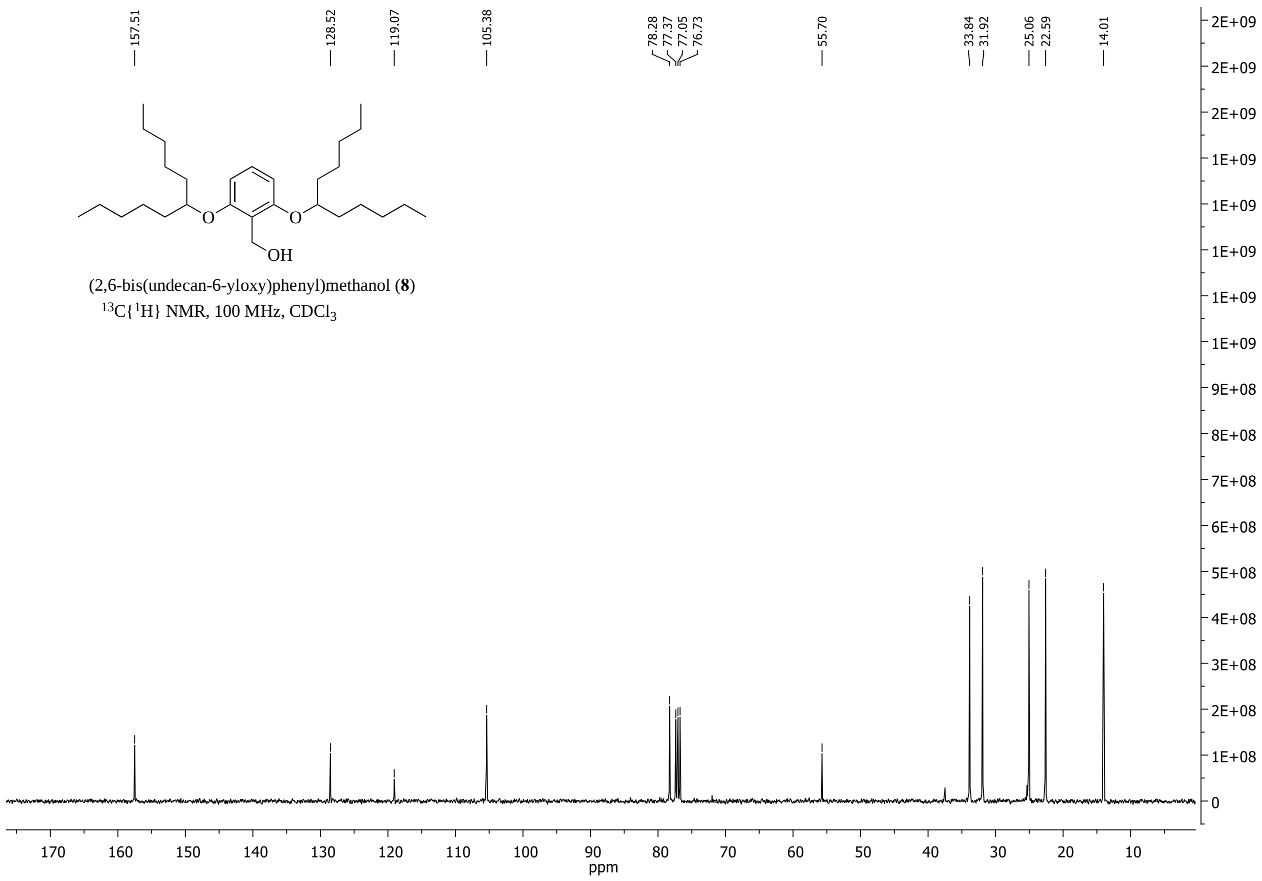

(2,6-bis(undecan-6-yloxy)phenyl)methanol (**8**)
$^{13}C\{^{1}H\}$ NMR, 100 MHz, $CDCl_3$

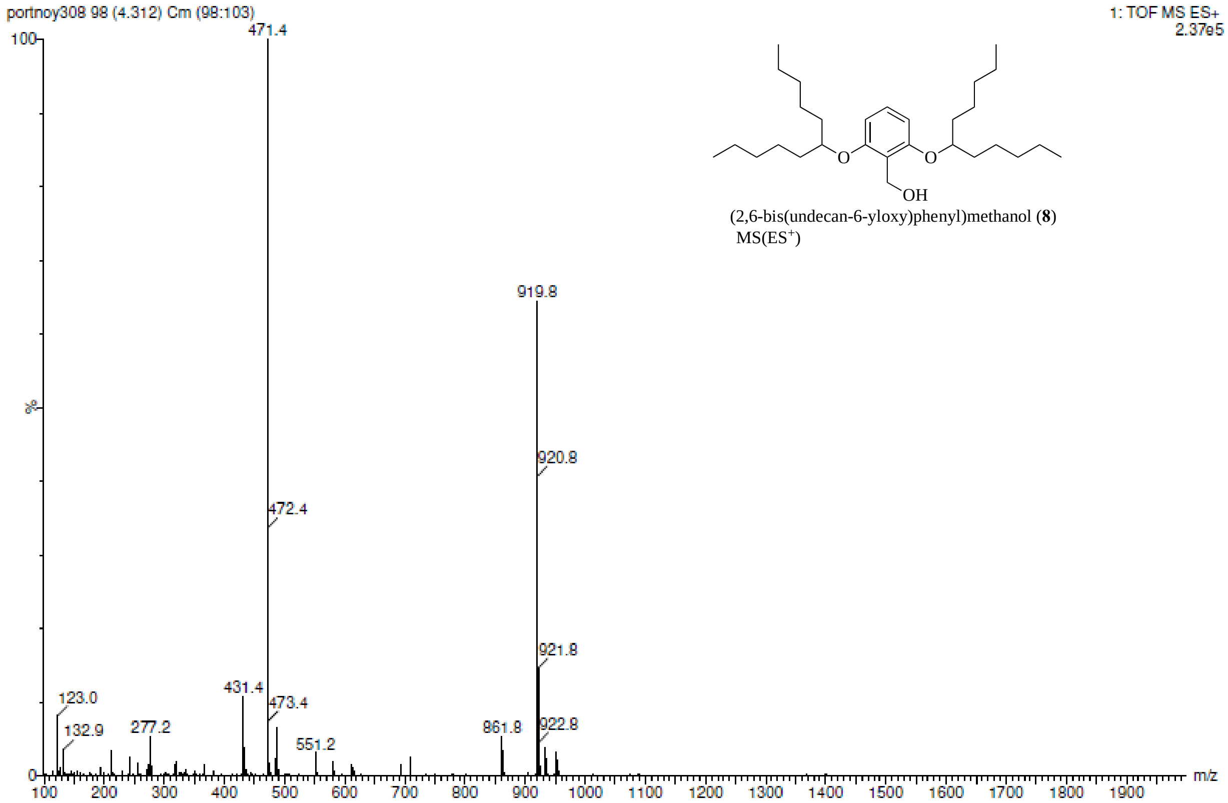

(2,6-bis(undecan-6-yloxy)phenyl)methanol (**8**)
MS(ES^{+})

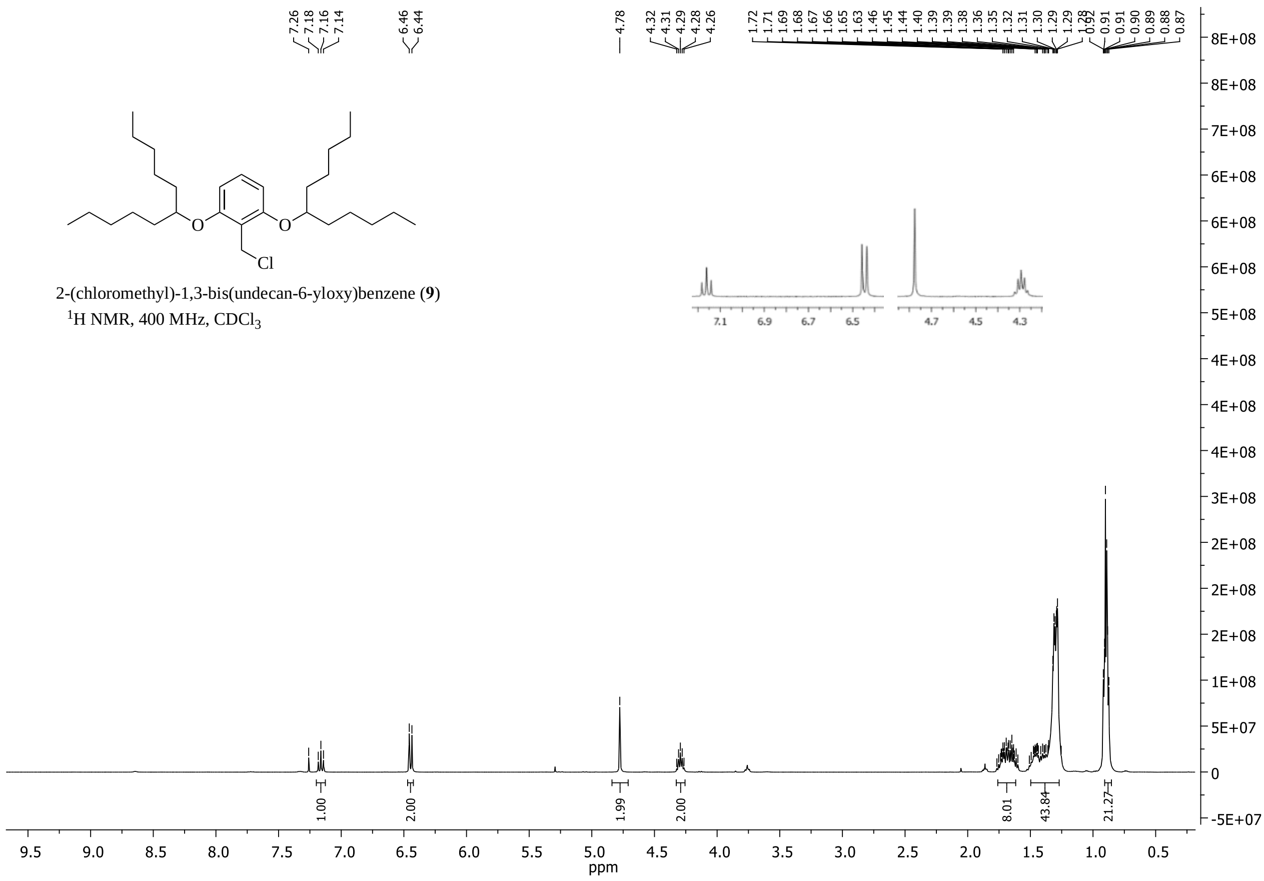

7.26
7.18
7.16
7.14
6.46
6.44
4.78
4.32
4.31
4.29
4.28
4.26
1.72
1.71
1.69
1.68
1.67
1.66
1.65
1.63
1.46
1.45
1.44
1.40
1.39
1.39
1.38
1.36
1.35
1.32
1.31
1.30
1.29
1.29
1.28
0.92
0.91
0.91
0.90
0.89
0.88
0.87
O
O
Cl
2-(chloromethyl)-1,3-bis(undecan-6-yloxy)benzene (**9**)
^{1}H NMR, 400 MHz, $CDCl_3$
1.00
2.00
1.99
2.00
8.01
43.84
21.27
ppm

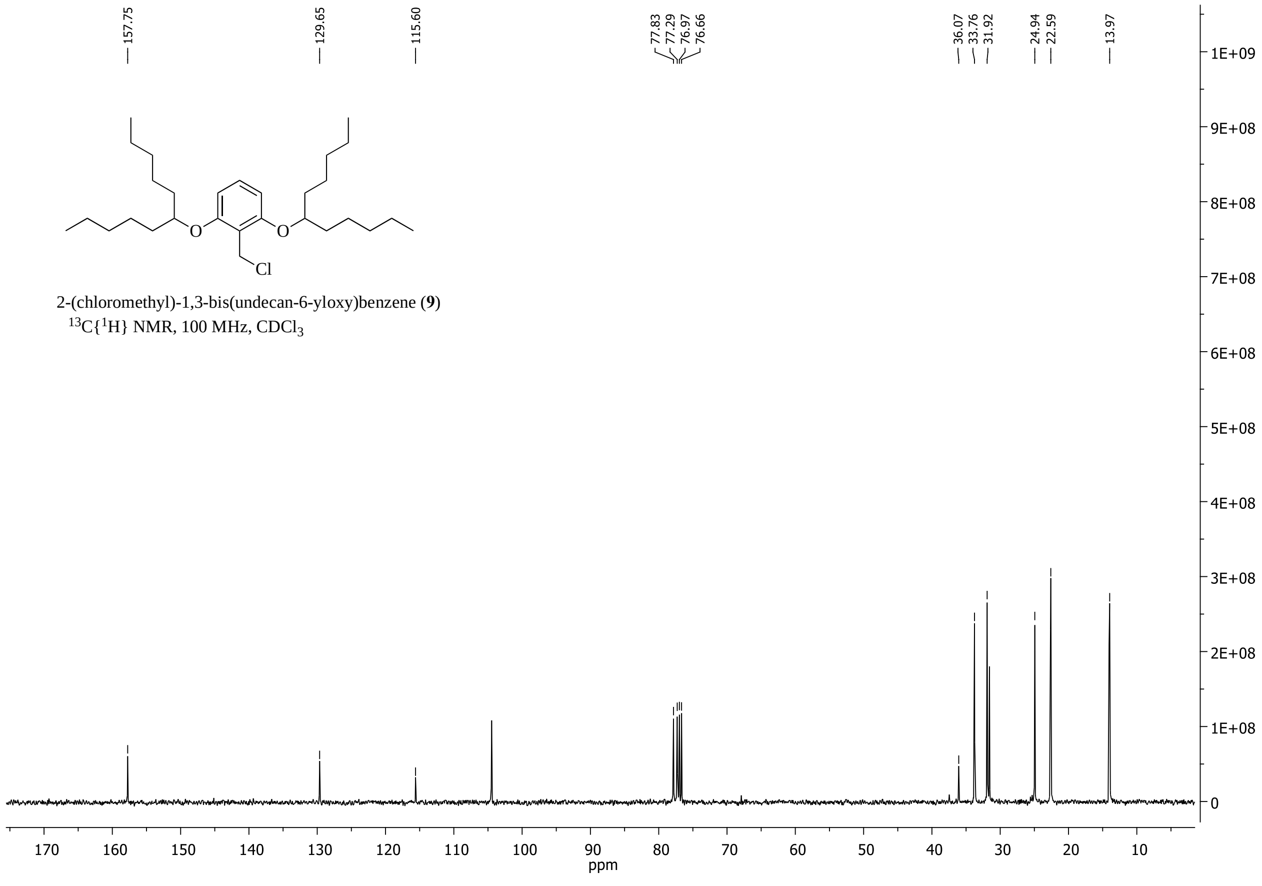

157.75
129.65
115.60
77.83
77.29
76.97
76.66
36.07
33.76
31.92
24.94
22.59
13.97
O
O
Cl
2-(chloromethyl)-1,3-bis(undecan-6-yloxy)benzene (**9**)
^{13}C{^{1}H} NMR, 100 MHz, $CDCl_3$
ppm

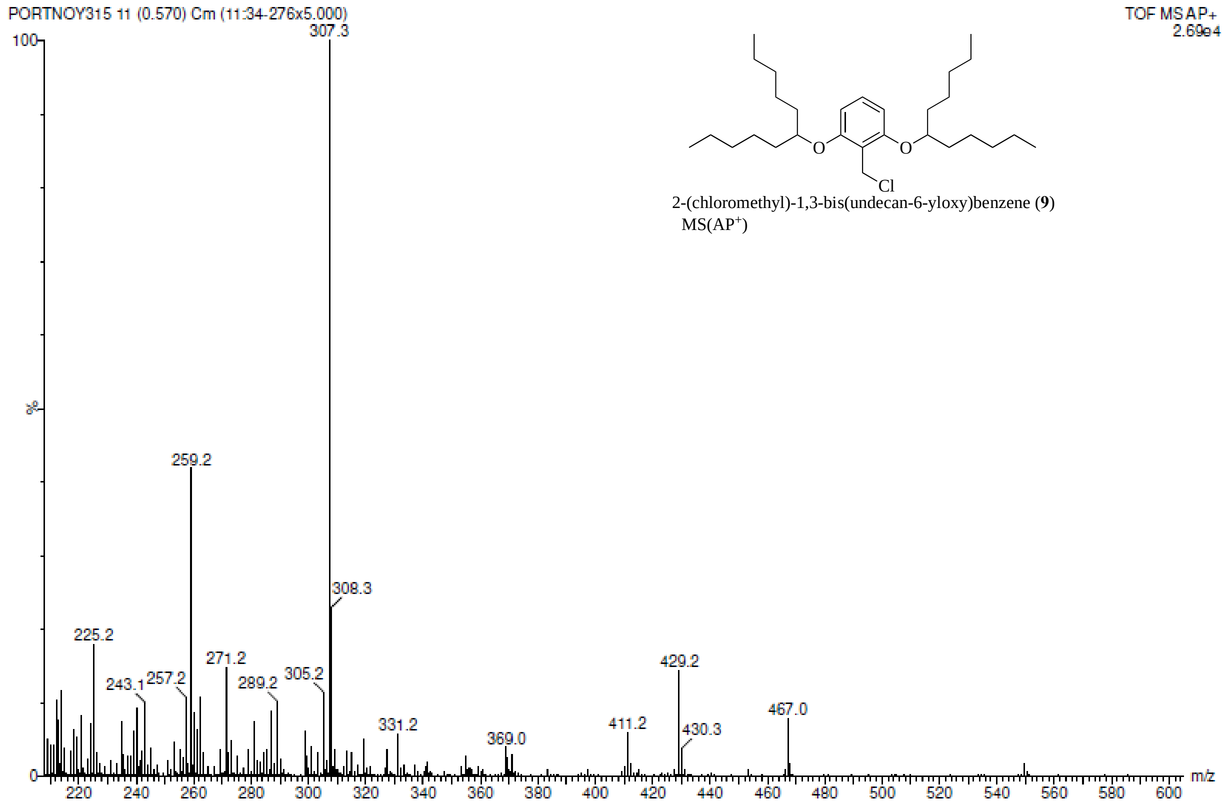

2-(chloromethyl)-1,3-bis(undecan-6-yloxy)benzene (**9**)
MS(AP$^+$)

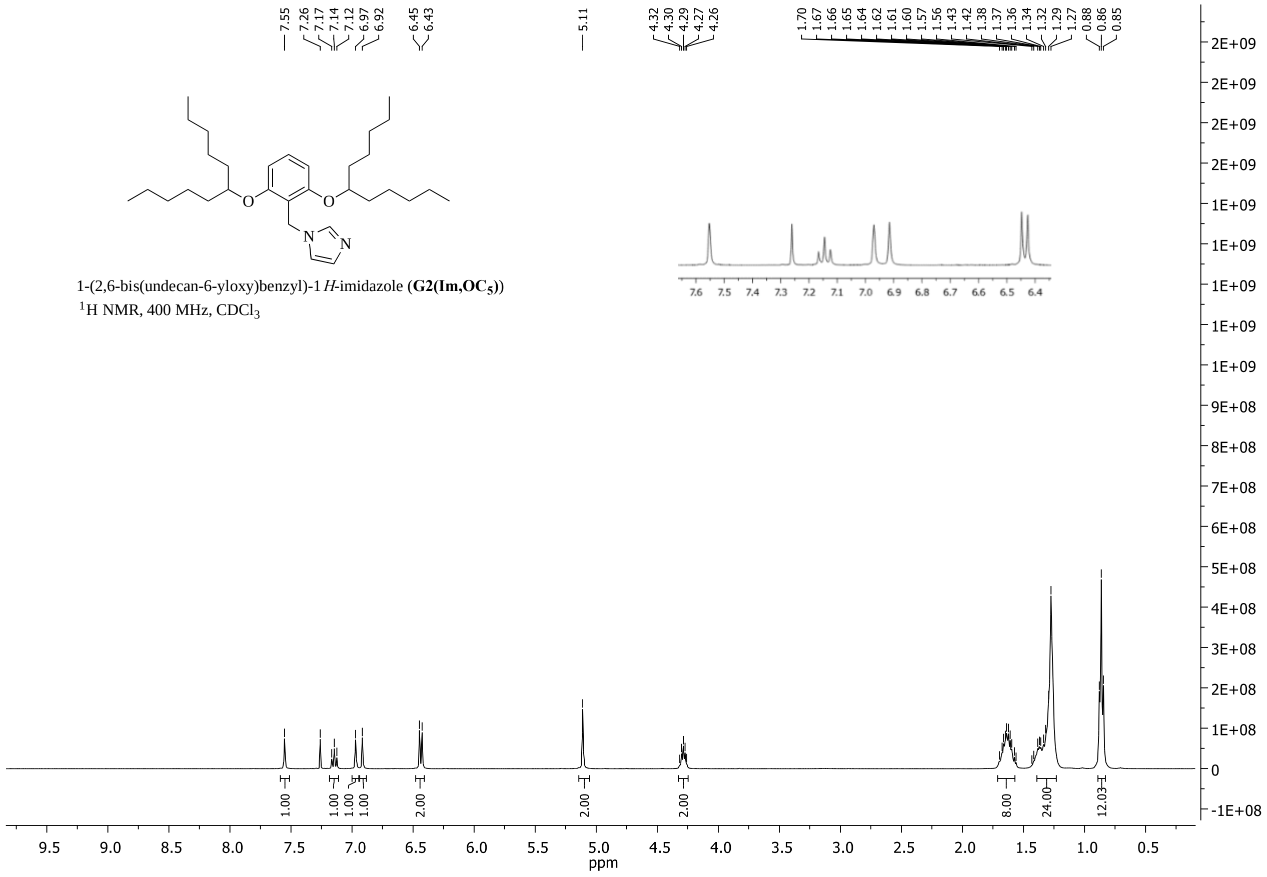

1-(2,6-bis(undecan-6-yloxy)benzyl)-1*H*-imidazole (**G2(Im,OC$_5$)**)
^{1}H NMR, 400 MHz, CDCl$_3$

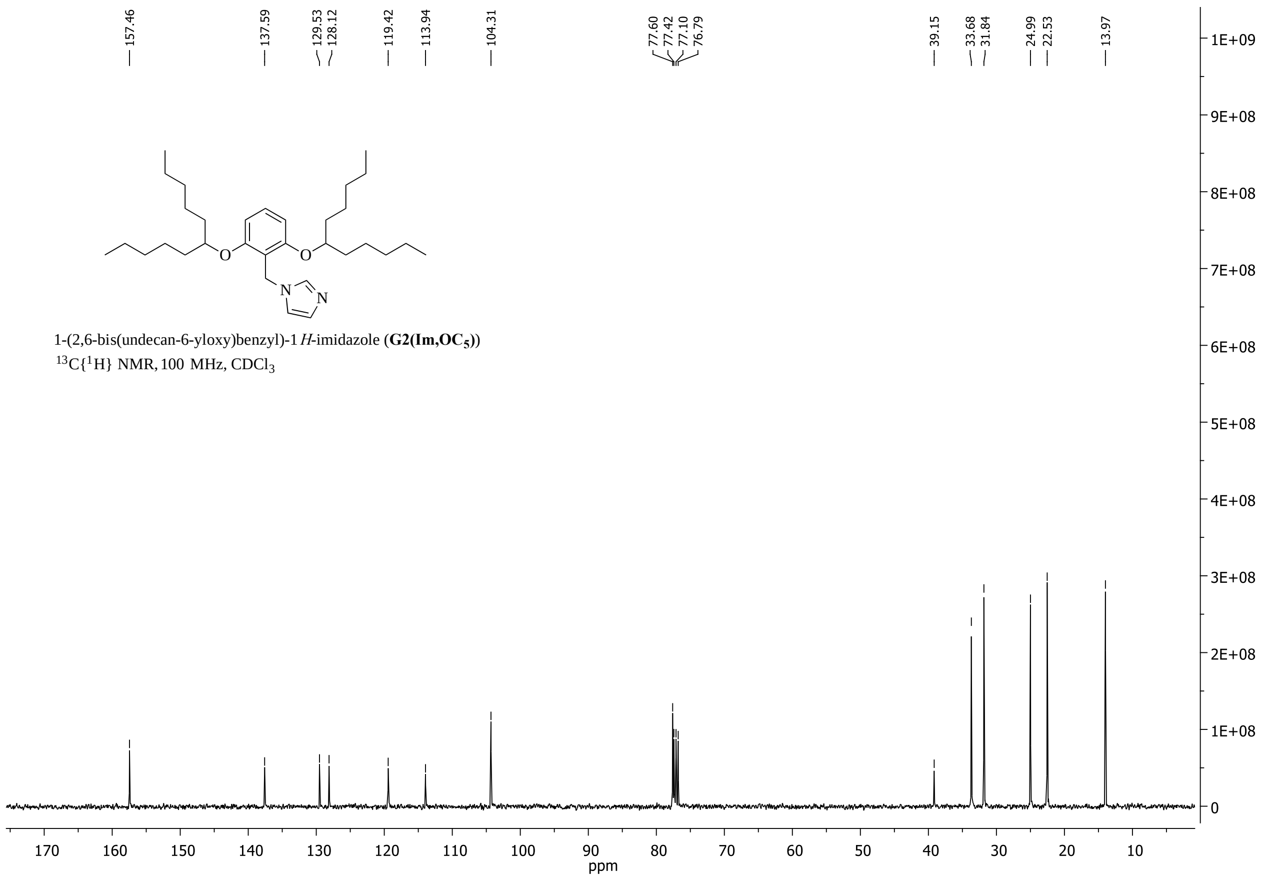
157.46
137.59
129.53
128.12
119.42
113.94
104.31
77.60
77.42
77.10
76.79
39.15
33.68
31.84
24.99
22.53
13.97
1-(2,6-bis(undecan-6-yloxy)benzyl)-1*H*-imidazole (**G2(Im,OC5)**)
13C{1H} NMR, 100 MHz, CDCl3
ppm

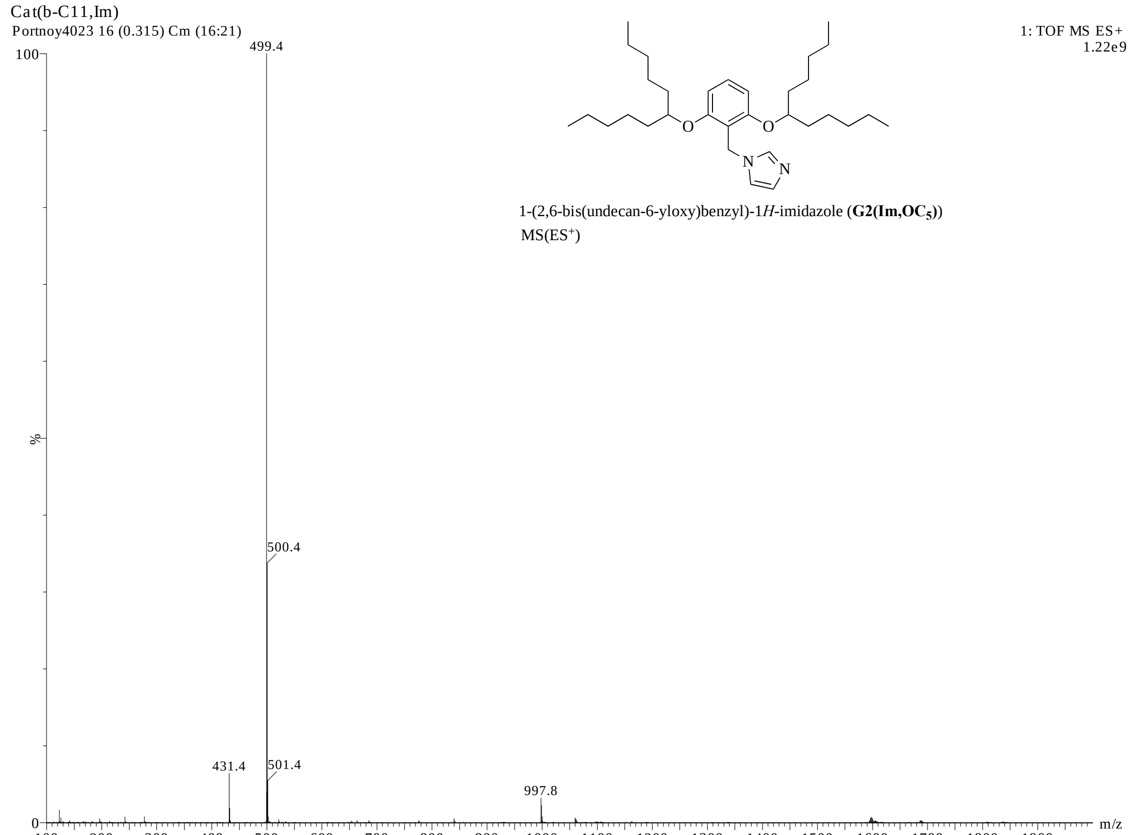
Cat(b-C11,Im)
Portnoy4023 16 (0.315) Cm (16:21)
1: TOF MS ES+
1.22e9
499.4
500.4
501.4
431.4
997.8
%
m/z
1-(2,6-bis(undecan-6-yloxy)benzyl)-1*H*-imidazole (**G2(Im,OC5)**)
MS(ES+)

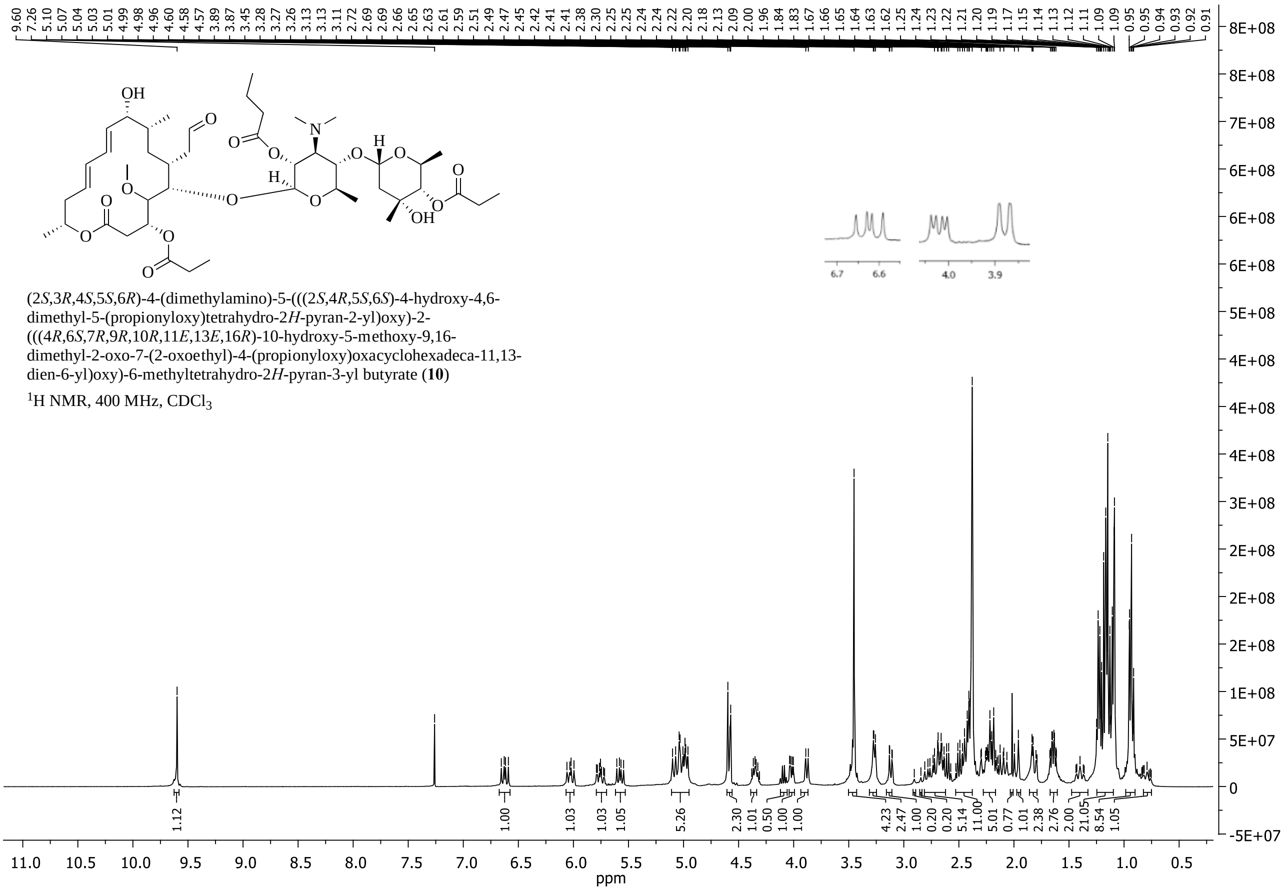

(2*S*,3*R*,4*S*,5*S*,6*R*)-4-(dimethylamino)-5-(((2*S*,4*R*,5*S*,6*S*)-4-hydroxy-4,6-dimethyl-5-(propionyloxy)tetrahydro-2*H*-pyran-2-yl)oxy)-2-(((4*R*,6*S*,7*R*,9*R*,10*R*,11*E*,13*E*,16*R*)-10-hydroxy-5-methoxy-9,16-dimethyl-2-oxo-7-(2-oxoethyl)-4-(propionyloxy)oxacyclohexadeca-11,13-dien-6-yl)oxy)-6-methyltetrahydro-2*H*-pyran-3-yl butyrate (**10**)

^{1}H NMR, 400 MHz, $CDCl_3$

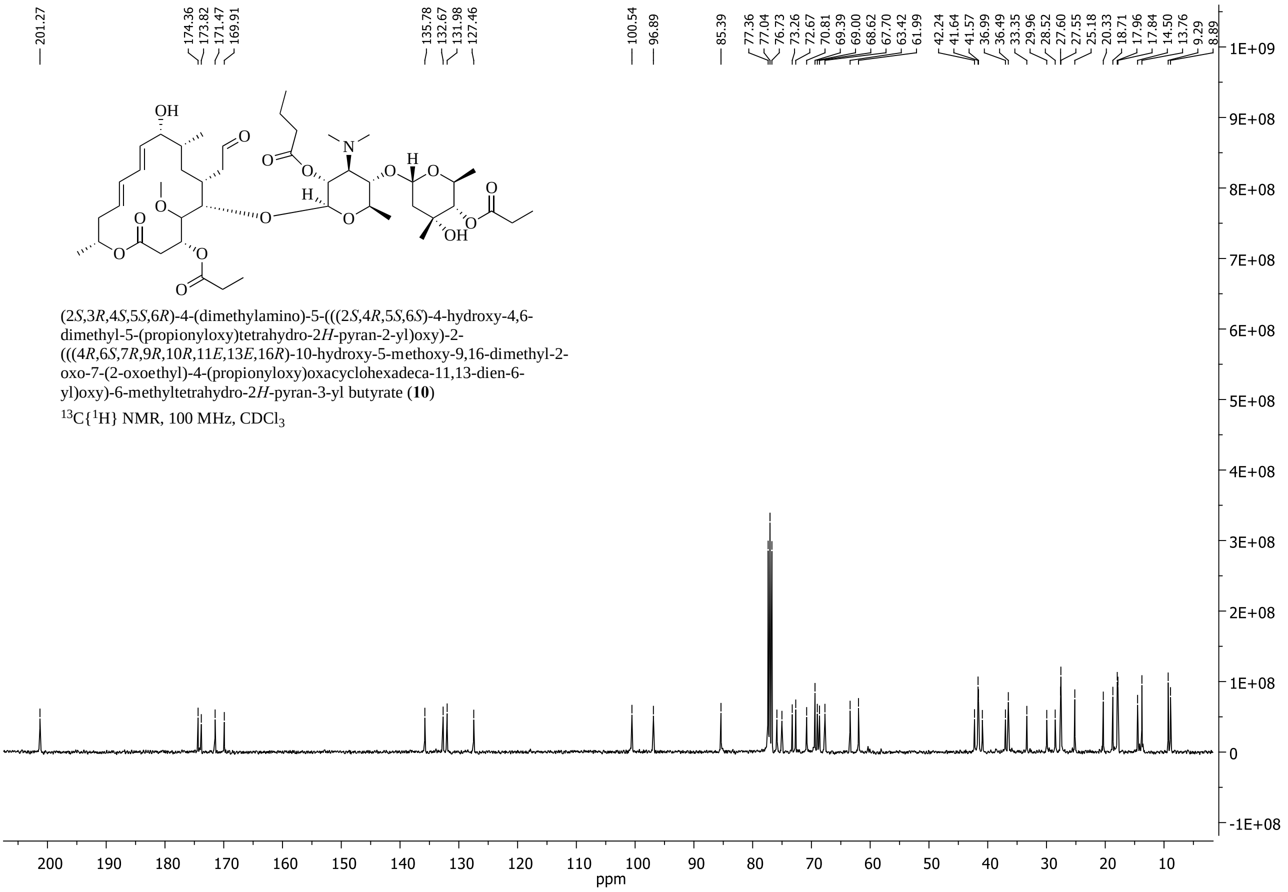

(2*S*,3*R*,4*S*,5*S*,6*R*)-4-(dimethylamino)-5-(((2*S*,4*R*,5*S*,6*S*)-4-hydroxy-4,6-dimethyl-5-(propionyloxy)tetrahydro-2*H*-pyran-2-yl)oxy)-2-(((4*R*,6*S*,7*R*,9*R*,10*R*,11*E*,13*E*,16*R*)-10-hydroxy-5-methoxy-9,16-dimethyl-2-oxo-7-(2-oxoethyl)-4-(propionyloxy)oxacyclohexadeca-11,13-dien-6-yl)oxy)-6-methyltetrahydro-2*H*-pyran-3-yl butyrate (**10**)

^{13}C{^{1}H} NMR, 100 MHz, $CDCl_3$

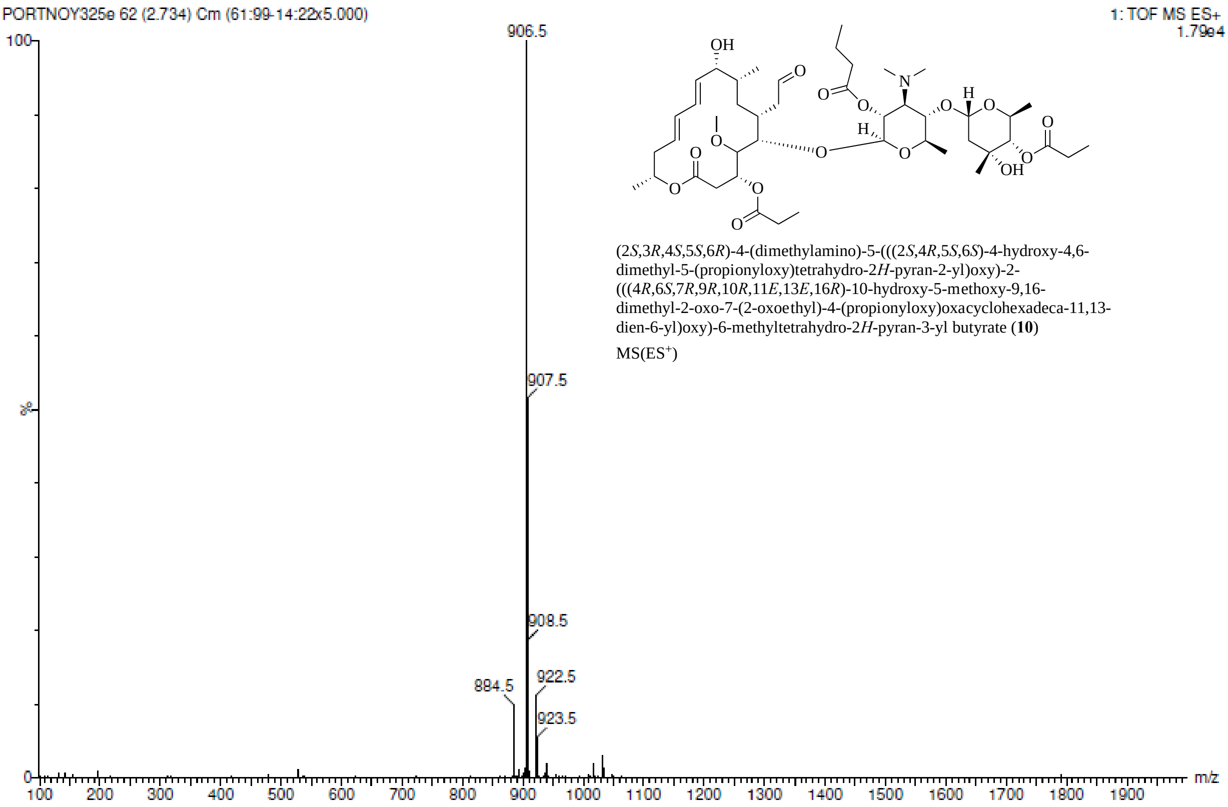

(2*S*,3*R*,4*S*,5*S*,6*R*)-4-(dimethylamino)-5-(((2*S*,4*R*,5*S*,6*S*)-4-hydroxy-4,6-dimethyl-5-(propionyloxy)tetrahydro-2*H*-pyran-2-yl)oxy)-2-(((4*R*,6*S*,7*R*,9*R*,10*R*,11*E*,13*E*,16*R*)-10-hydroxy-5-methoxy-9,16-dimethyl-2-oxo-7-(2-oxoethyl)-4-(propionyloxy)oxacyclohexadeca-11,13-dien-6-yl)oxy)-6-methyltetrahydro-2*H*-pyran-3-yl butyrate (**10**)

MS(ES$^+$)

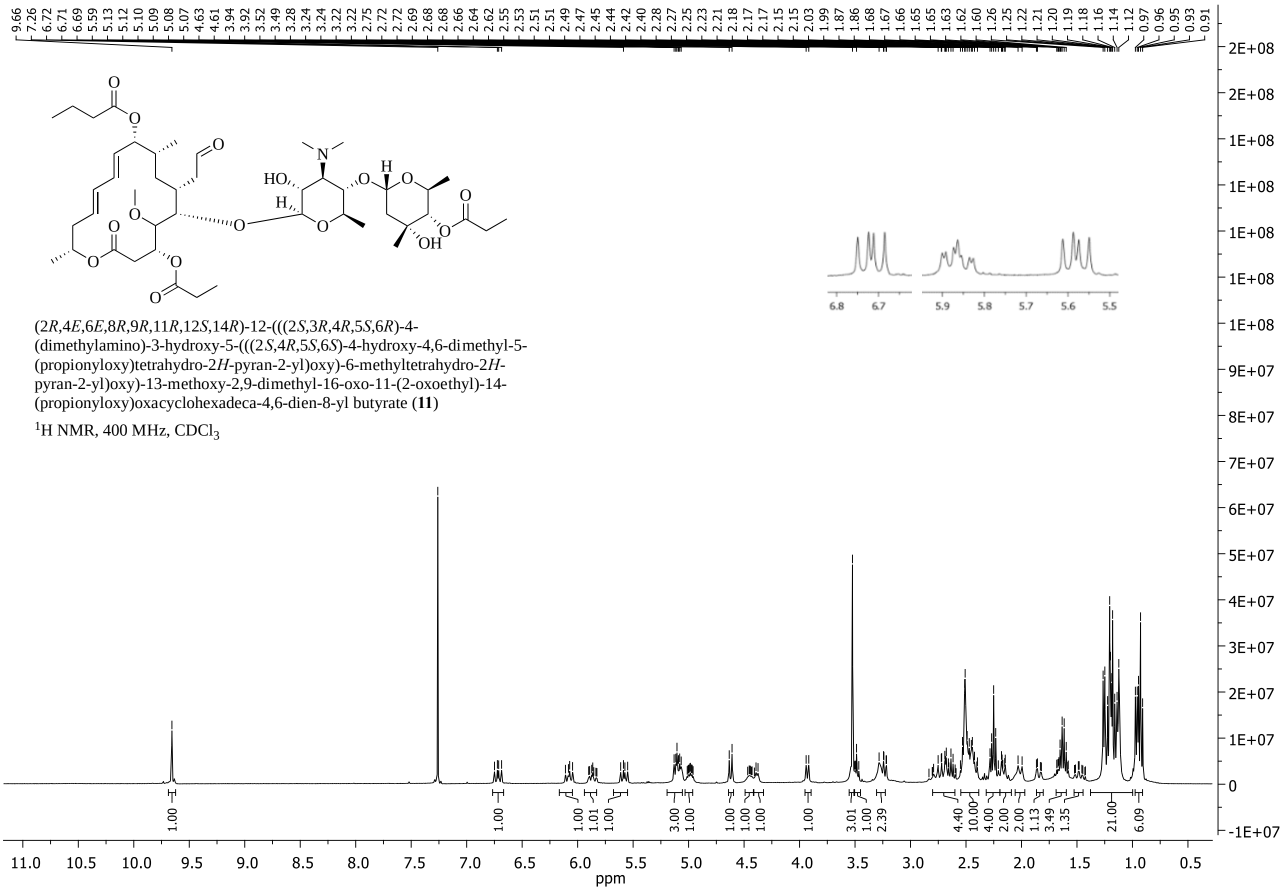

(2*R*,4*E*,6*E*,8*R*,9*R*,11*R*,12*S*,14*R*)-12-(((2*S*,3*R*,4*R*,5*S*,6*R*)-4-(dimethylamino)-3-hydroxy-5-(((2*S*,4*R*,5*S*,6*S*)-4-hydroxy-4,6-dimethyl-5-(propionyloxy)tetrahydro-2*H*-pyran-2-yl)oxy)-6-methyltetrahydro-2*H*-pyran-2-yl)oxy)-13-methoxy-2,9-dimethyl-16-oxo-11-(2-oxoethyl)-14-(propionyloxy)oxacyclohexadeca-4,6-dien-8-yl butyrate (**11**)

^{1}H NMR, 400 MHz, $CDCl_3$

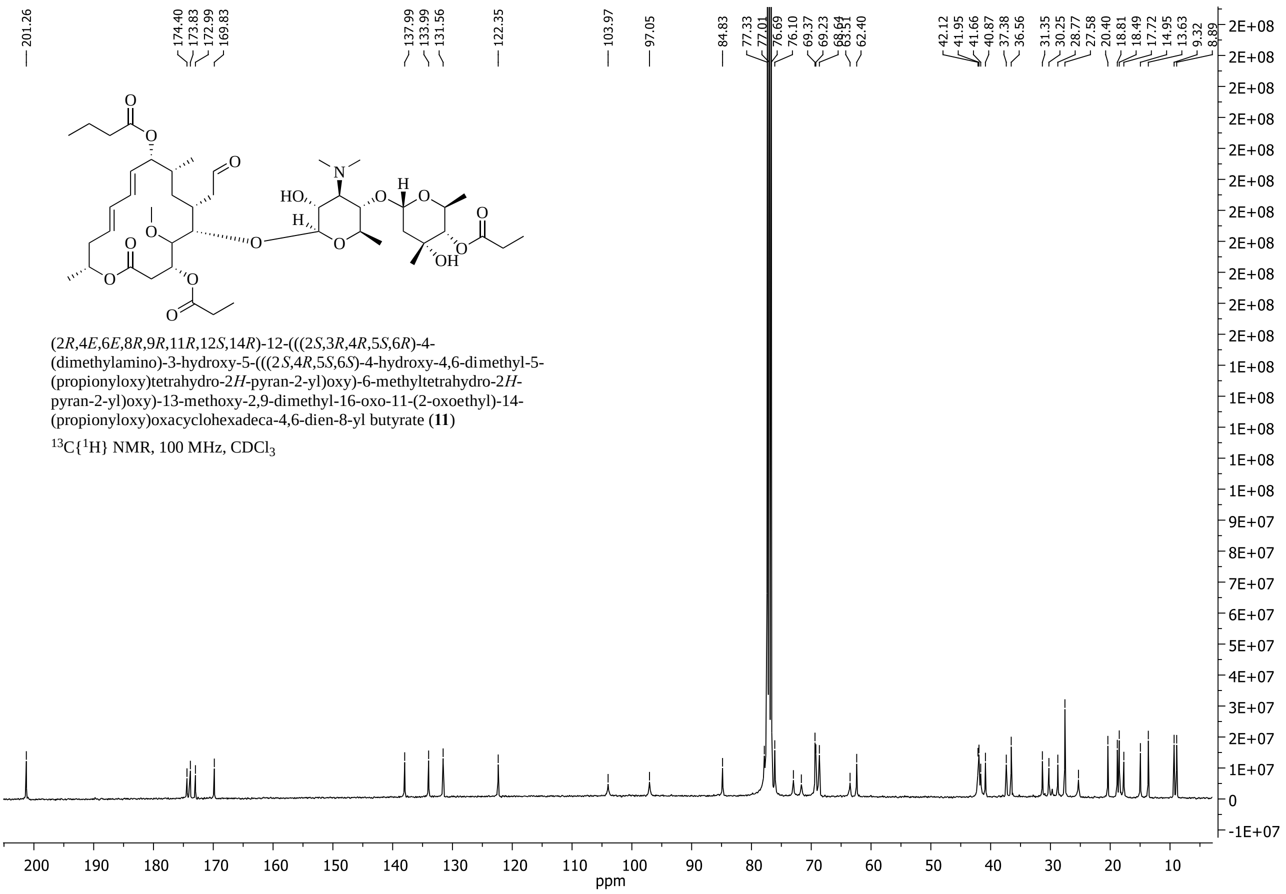

(2*R*,4*E*,6*E*,8*R*,9*R*,11*R*,12*S*,14*R*)-12-(((2*S*,3*R*,4*R*,5*S*,6*R*)-4-(dimethylamino)-3-hydroxy-5-(((2*S*,4*R*,5*S*,6*S*)-4-hydroxy-4,6-dimethyl-5-(propionyloxy)tetrahydro-2*H*-pyran-2-yl)oxy)-6-methyltetrahydro-2*H*-pyran-2-yl)oxy)-13-methoxy-2,9-dimethyl-16-oxo-11-(2-oxoethyl)-14-(propionyloxy)oxacyclohexadeca-4,6-dien-8-yl butyrate (**11**)

$^{13}C\{^{1}H\}$ NMR, 100 MHz, $CDCl_3$

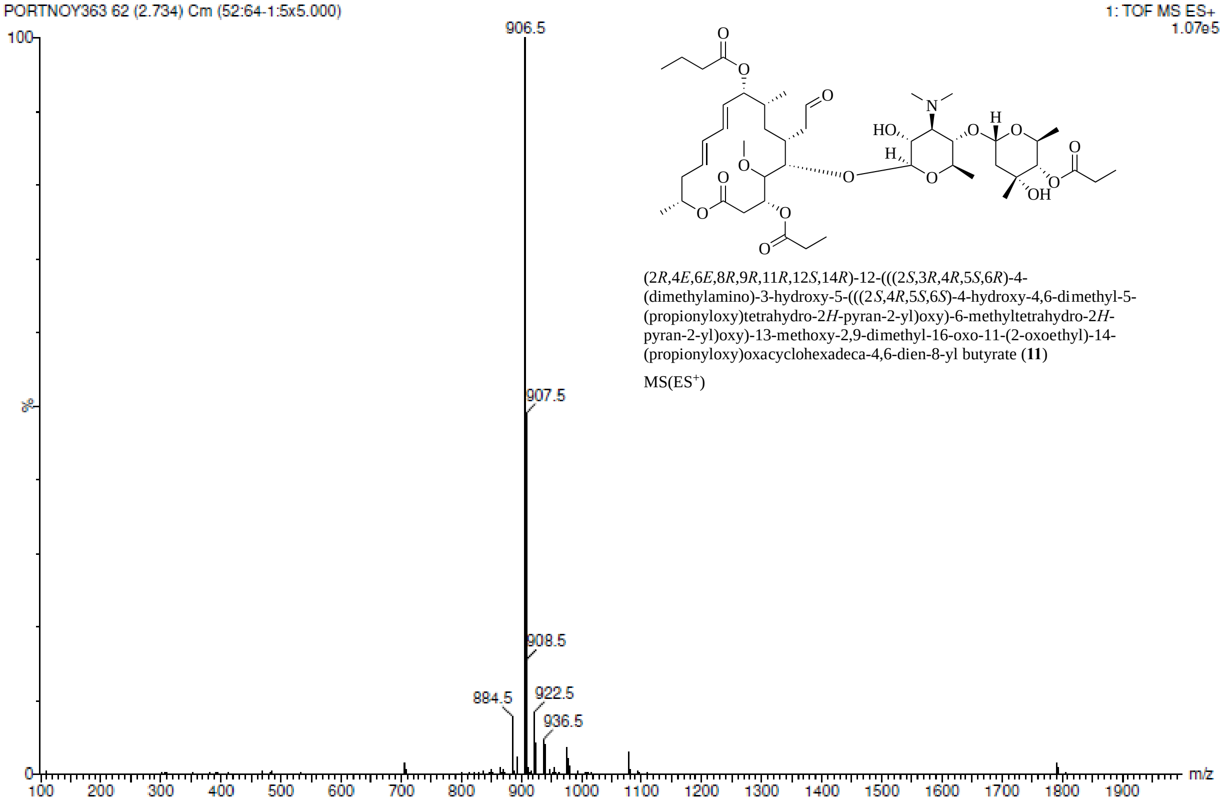

(2*R*,4*E*,6*E*,8*R*,9*R*,11*R*,12*S*,14*R*)-12-(((2*S*,3*R*,4*R*,5*S*,6*R*)-4-(dimethylamino)-3-hydroxy-5-(((2*S*,4*R*,5*S*,6*S*)-4-hydroxy-4,6-dimethyl-5-(propionyloxy)tetrahydro-2*H*-pyran-2-yl)oxy)-6-methyltetrahydro-2*H*-pyran-2-yl)oxy)-13-methoxy-2,9-dimethyl-16-oxo-11-(2-oxoethyl)-14-(propionyloxy)oxacyclohexadeca-4,6-dien-8-yl butyrate (**11**)

MS(ES^{+})

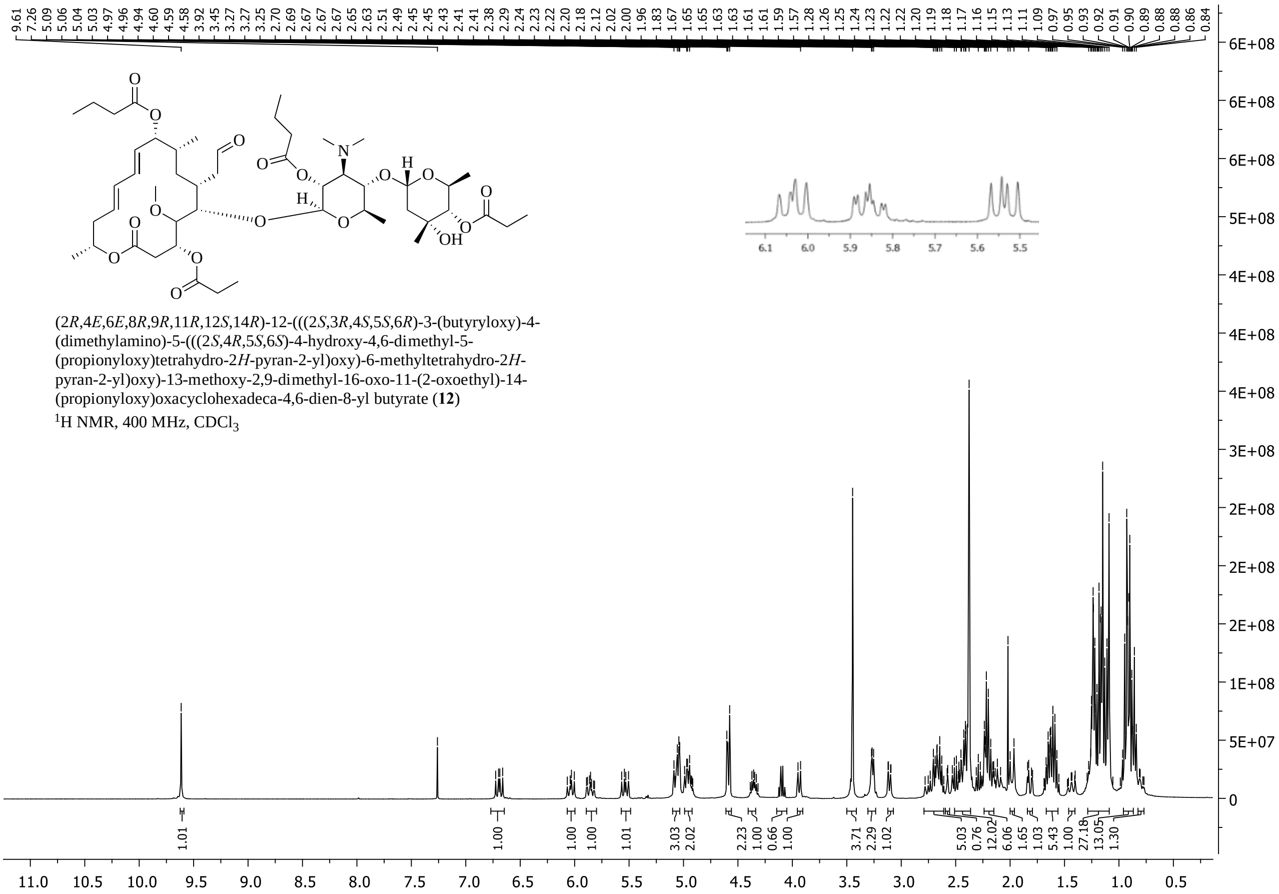
9.61 7.26 5.09 5.06 5.04 5.03 4.97 4.96 4.94 4.60 4.59 4.58 3.92 3.45 3.27 3.27 3.25 2.70 2.69 2.67 2.67 2.65 2.63 2.51 2.49 2.45 2.45 2.43 2.41 2.41 2.38 2.29 2.24 2.23 2.22 2.20 2.18 2.12 2.02 2.00 1.96 1.83 1.67 1.65 1.65 1.63 1.63 1.61 1.61 1.59 1.57 1.28 1.26 1.25 1.24 1.23 1.22 1.22 1.20 1.19 1.18 1.17 1.16 1.15 1.13 1.11 1.09 0.97 0.95 0.93 0.92 0.91 0.90 0.89 0.88 0.86 0.84
(2*R*,4*E*,6*E*,8*R*,9*R*,11*R*,12*S*,14*R*)-12-(((2*S*,3*R*,4*S*,5*S*,6*R*)-3-(butyryloxy)-4-(dimethylamino)-5-(((2*S*,4*R*,5*S*,6*S*)-4-hydroxy-4,6-dimethyl-5-(propionyloxy)tetrahydro-2*H*-pyran-2-yl)oxy)-6-methyltetrahydro-2*H*-pyran-2-yl)oxy)-13-methoxy-2,9-dimethyl-16-oxo-11-(2-oxoethyl)-14-(propionyloxy)oxacyclohexadeca-4,6-dien-8-yl butyrate (**12**)
^{1}H NMR, 400 MHz, $CDCl_3$
1.01 1.00 1.00 1.00 1.01 3.03 2.02 2.23 1.00 0.66 1.00 3.71 2.29 1.02 5.03 0.76 12.02 6.06 1.65 1.03 5.43 1.00 27.18 13.05 1.30

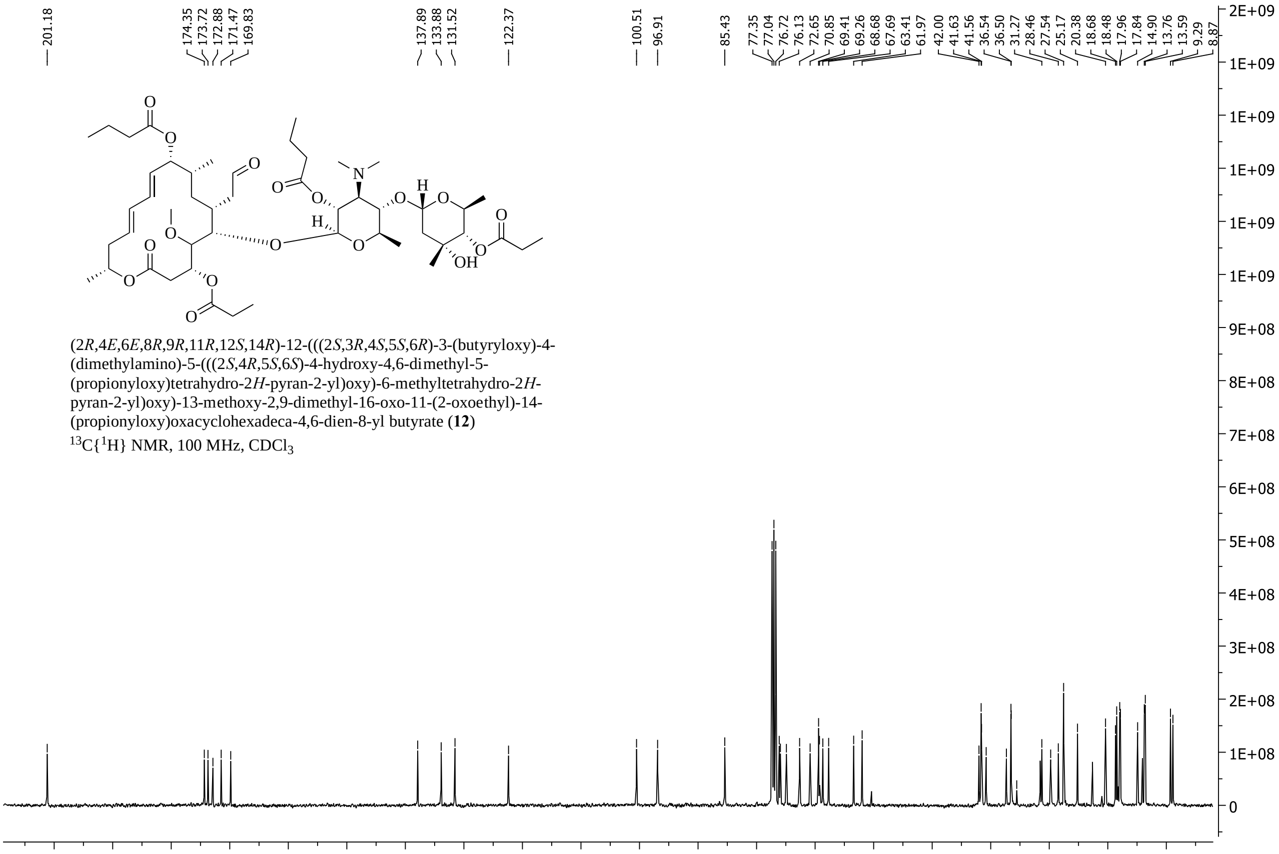
201.18 174.35 173.72 172.88 171.47 169.83 137.89 133.88 131.52 122.37 100.51 96.91 85.43 77.35 77.04 76.72 76.13 72.65 70.85 69.41 69.26 68.68 67.69 63.41 61.97 42.00 41.63 41.56 36.54 36.50 31.27 28.46 27.54 25.17 20.38 18.68 18.48 17.96 17.84 14.90 13.76 13.59 9.29 8.87
(2*R*,4*E*,6*E*,8*R*,9*R*,11*R*,12*S*,14*R*)-12-(((2*S*,3*R*,4*S*,5*S*,6*R*)-3-(butyryloxy)-4-(dimethylamino)-5-(((2*S*,4*R*,5*S*,6*S*)-4-hydroxy-4,6-dimethyl-5-(propionyloxy)tetrahydro-2*H*-pyran-2-yl)oxy)-6-methyltetrahydro-2*H*-pyran-2-yl)oxy)-13-methoxy-2,9-dimethyl-16-oxo-11-(2-oxoethyl)-14-(propionyloxy)oxacyclohexadeca-4,6-dien-8-yl butyrate (**12**)
^{13}C{^{1}H} NMR, 100 MHz, $CDCl_3$
ppm

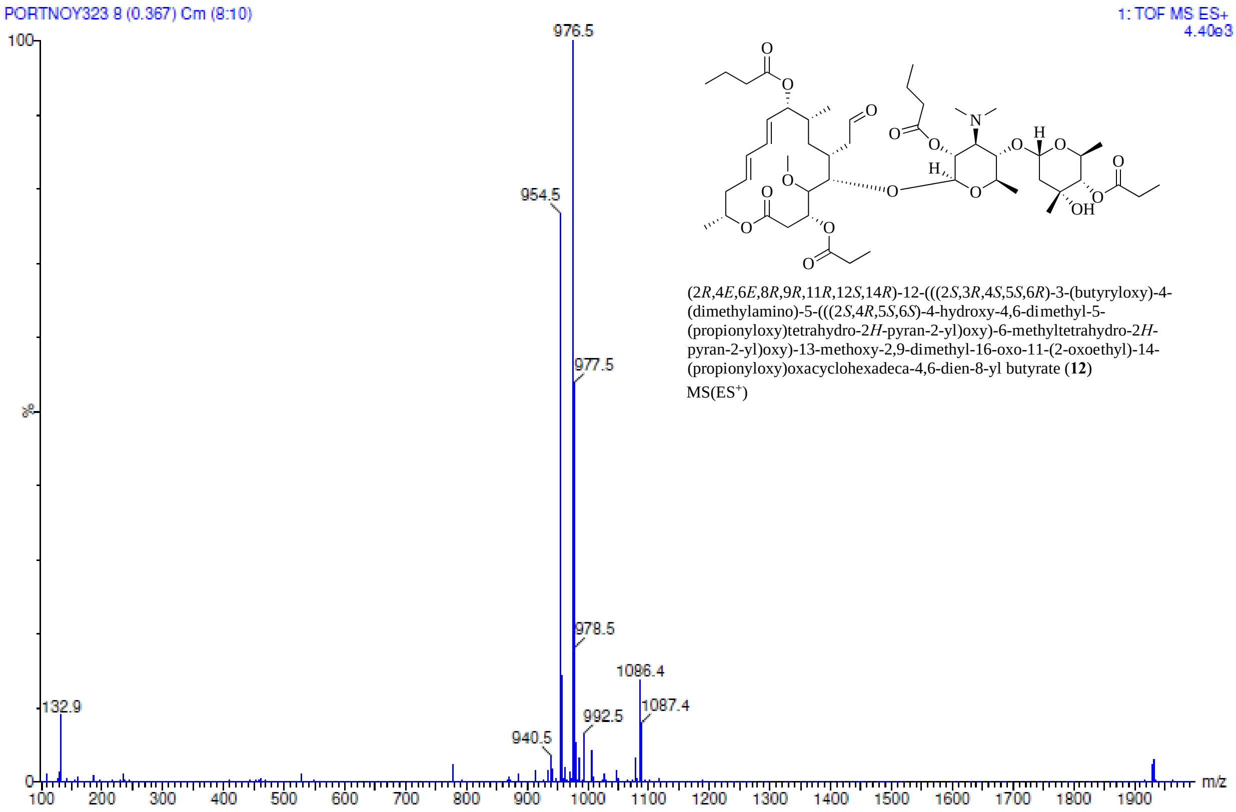

(2*R*,4*E*,6*E*,8*R*,9*R*,11*R*,12*S*,14*R*)-12-(((2*S*,3*R*,4*S*,5*S*,6*R*)-3-(butyryloxy)-4-(dimethylamino)-5-(((2*S*,4*R*,5*S*,6*S*)-4-hydroxy-4,6-dimethyl-5-(propionyloxy)tetrahydro-2*H*-pyran-2-yl)oxy)-6-methyltetrahydro-2*H*-pyran-2-yl)oxy)-13-methoxy-2,9-dimethyl-16-oxo-11-(2-oxoethyl)-14-(propionyloxy)oxacyclohexadeca-4,6-dien-8-yl butyrate (**12**)

MS(ES$^+$)

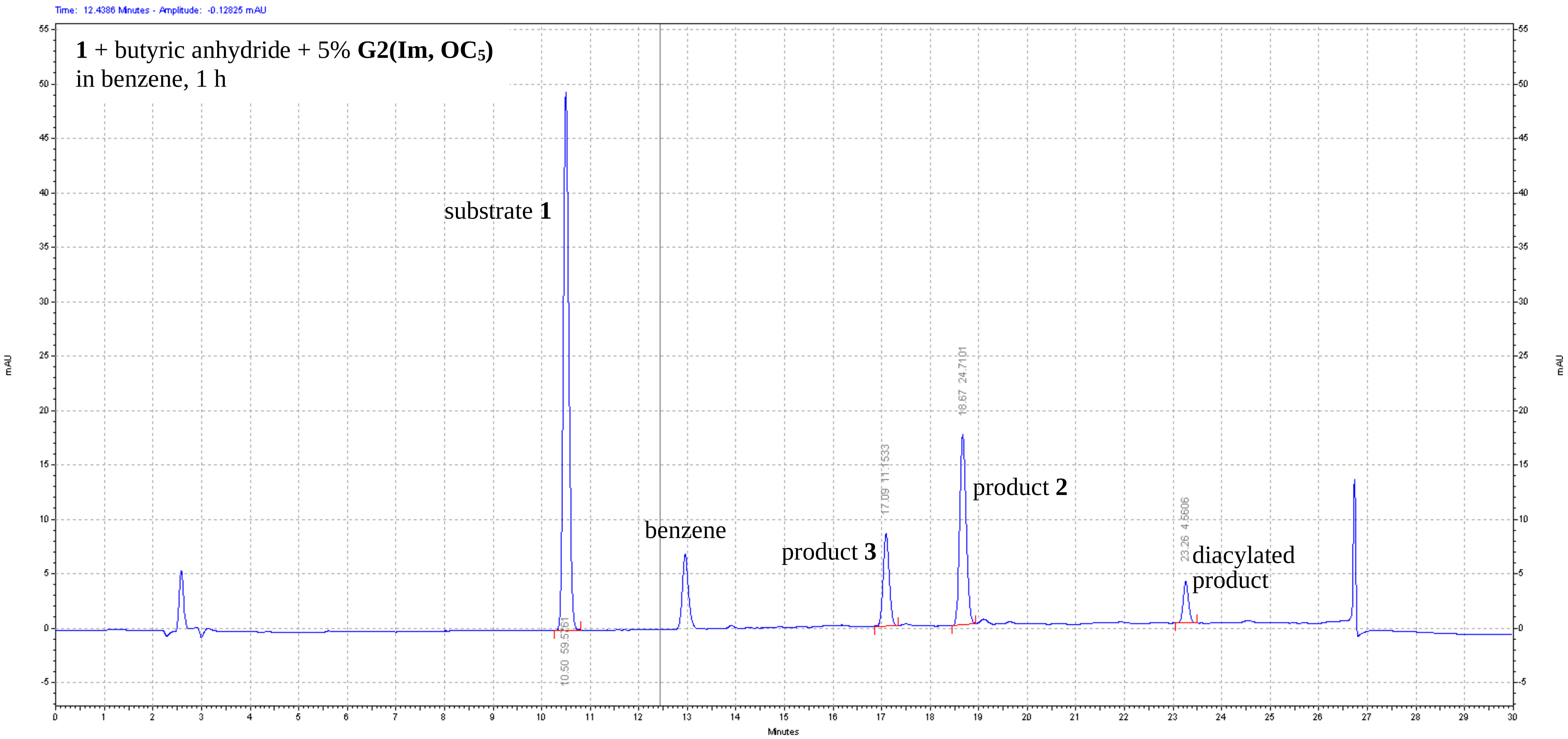
Time: 12.4386 Minutes - Amplitude: -0.12825 mAU
1 + butyric anhydride + 5% G2(Im, OC5)
in benzene, 1 h
substrate 1
benzene
product 3
product 2
diacylated
product
10.50 59.5161
17.09 11.1533
18.67 24.7101
23.26 4.5606
mAU
Minutes

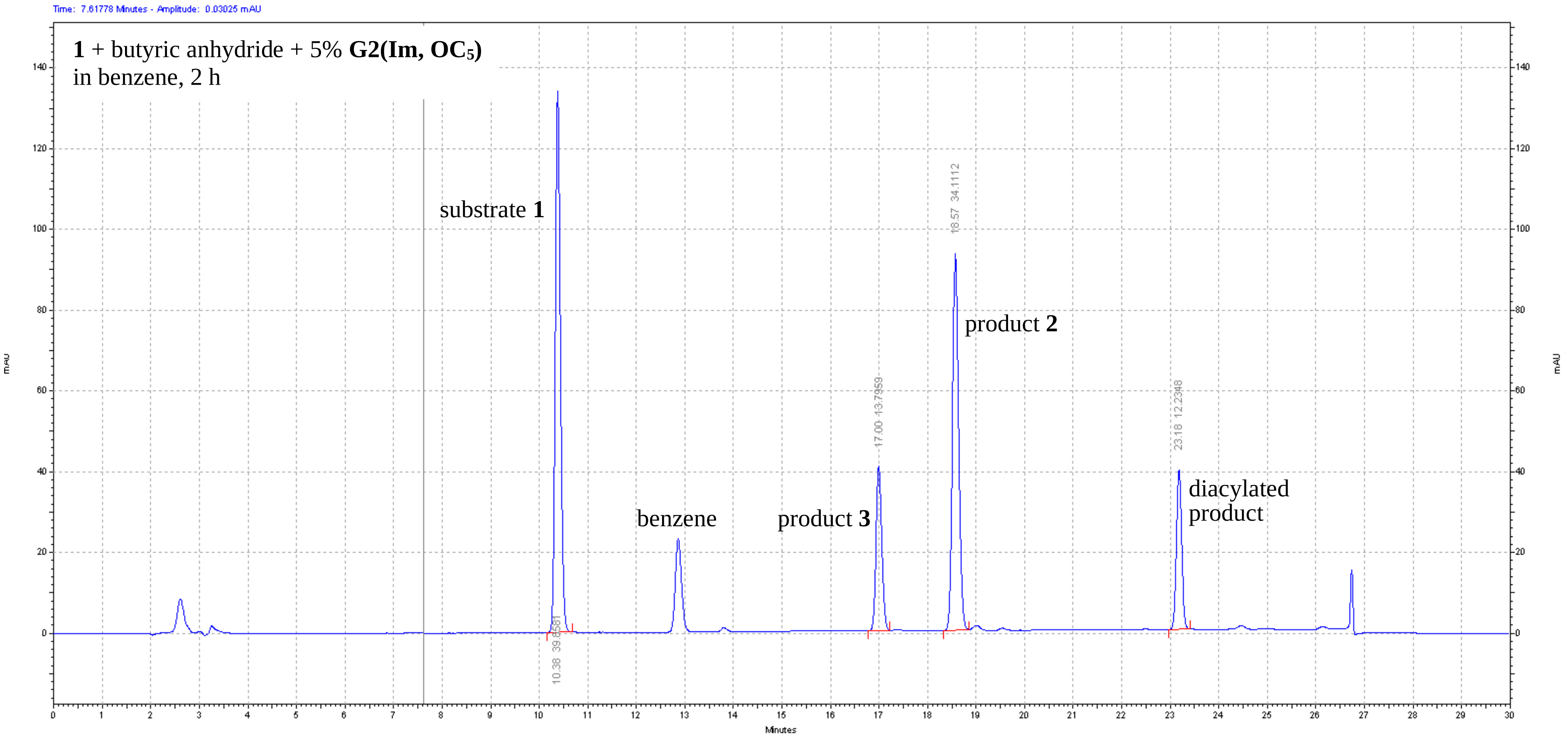
Time: 7.61778 Minutes - Amplitude: 0.03025 mAU
1 + butyric anhydride + 5% G2(Im, OC5)
in benzene, 2 h
substrate 1
benzene
product 3
product 2
diacylated
product
10.38 39.8581
17.00 13.7959
18.57 34.1112
23.18 12.2348
mAU
Minutes

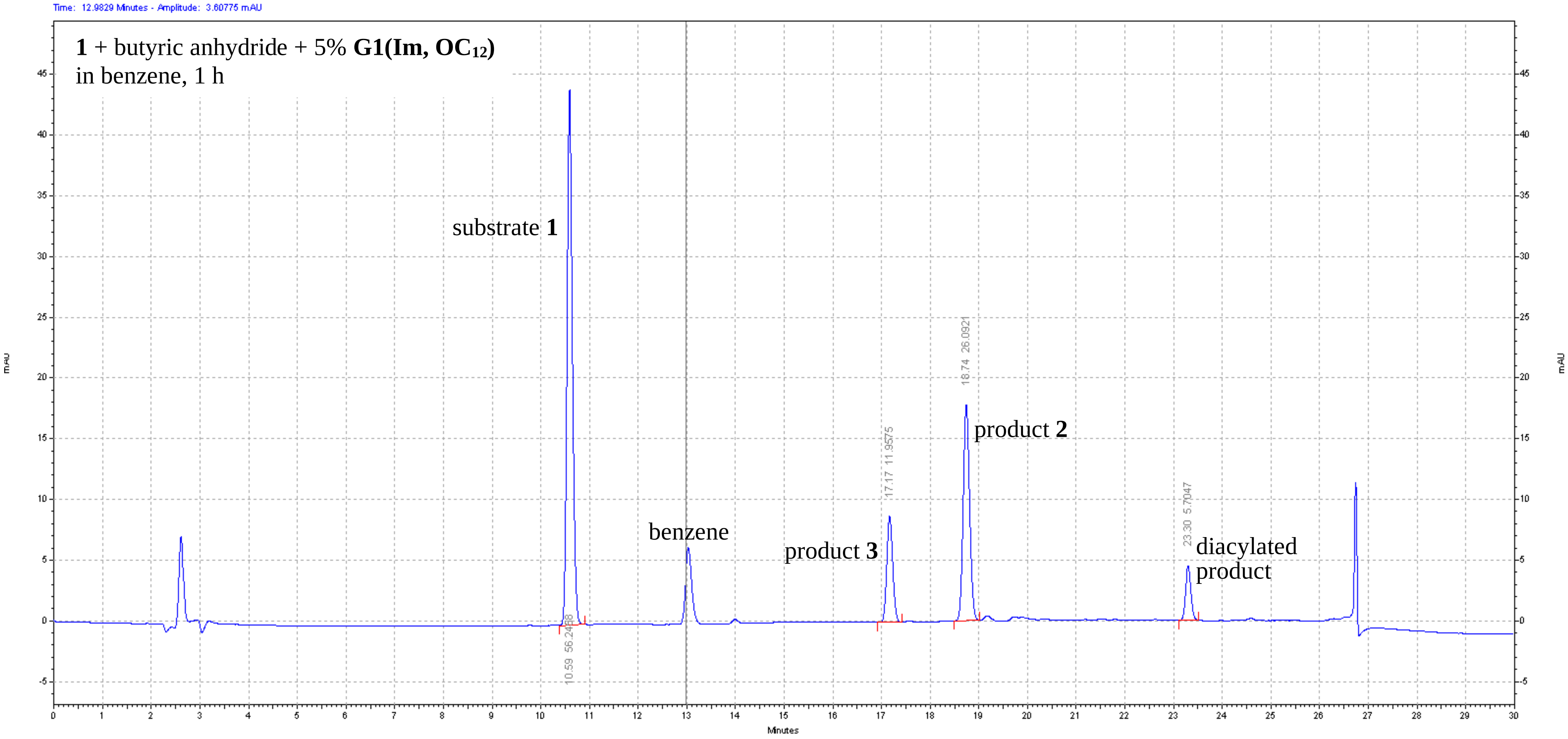
Time: 12.9829 Minutes - Amplitude: 3.60775 mAU
1 + butyric anhydride + 5% G1(Im, OC12)
in benzene, 1 h
substrate 1
benzene
product 3
product 2
diacylated
product
10.59 56.2488
17.17 11.9575
18.74 26.0921
23.30 5.7047
mAU
Minutes

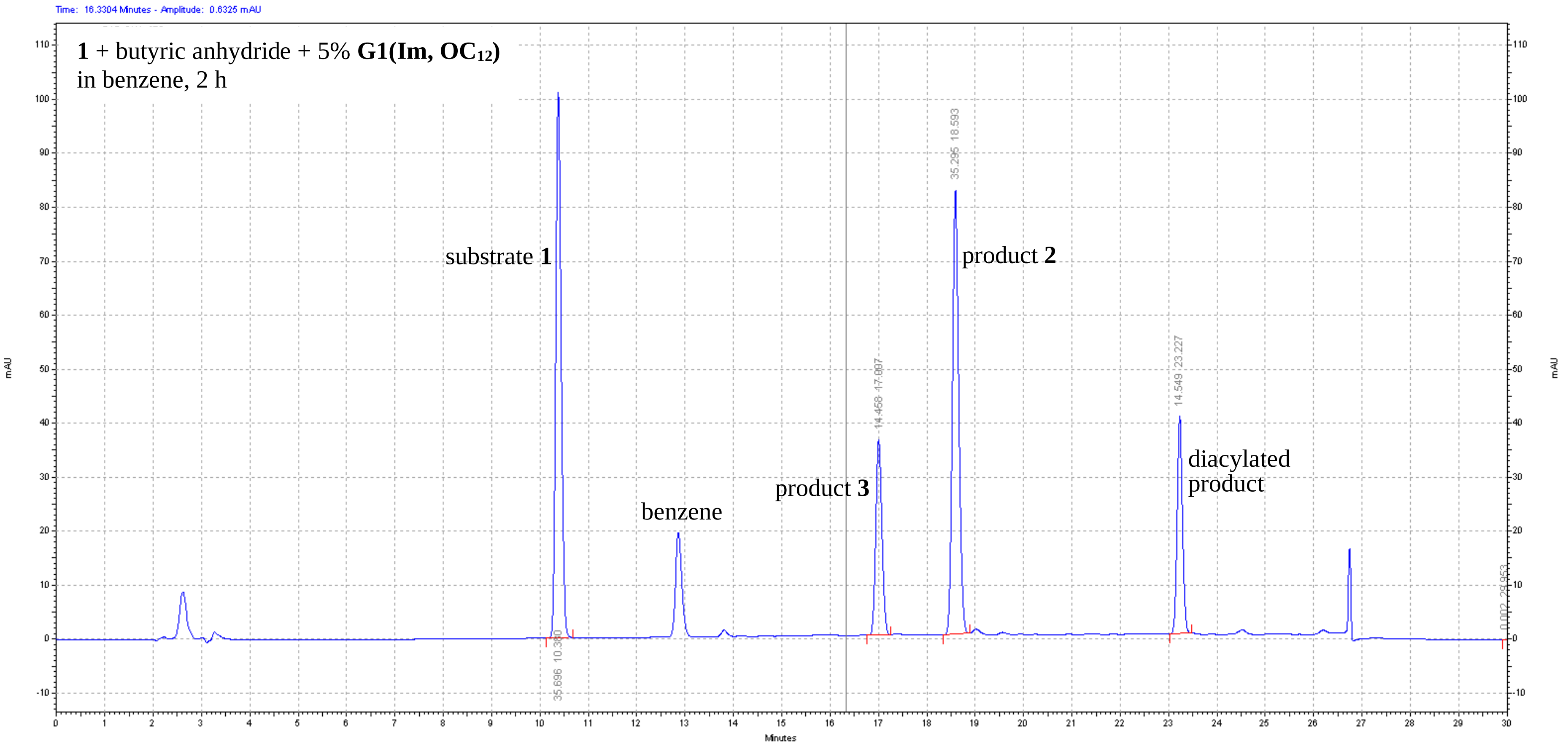

Time: 16.3304 Minutes - Amplitude: 0.6325 mAU
1 + butyric anhydride + 5% G1(Im, OC12)
in benzene, 2 h
substrate 1
benzene
product 3
product 2
diacylated
product
mAU
Minutes

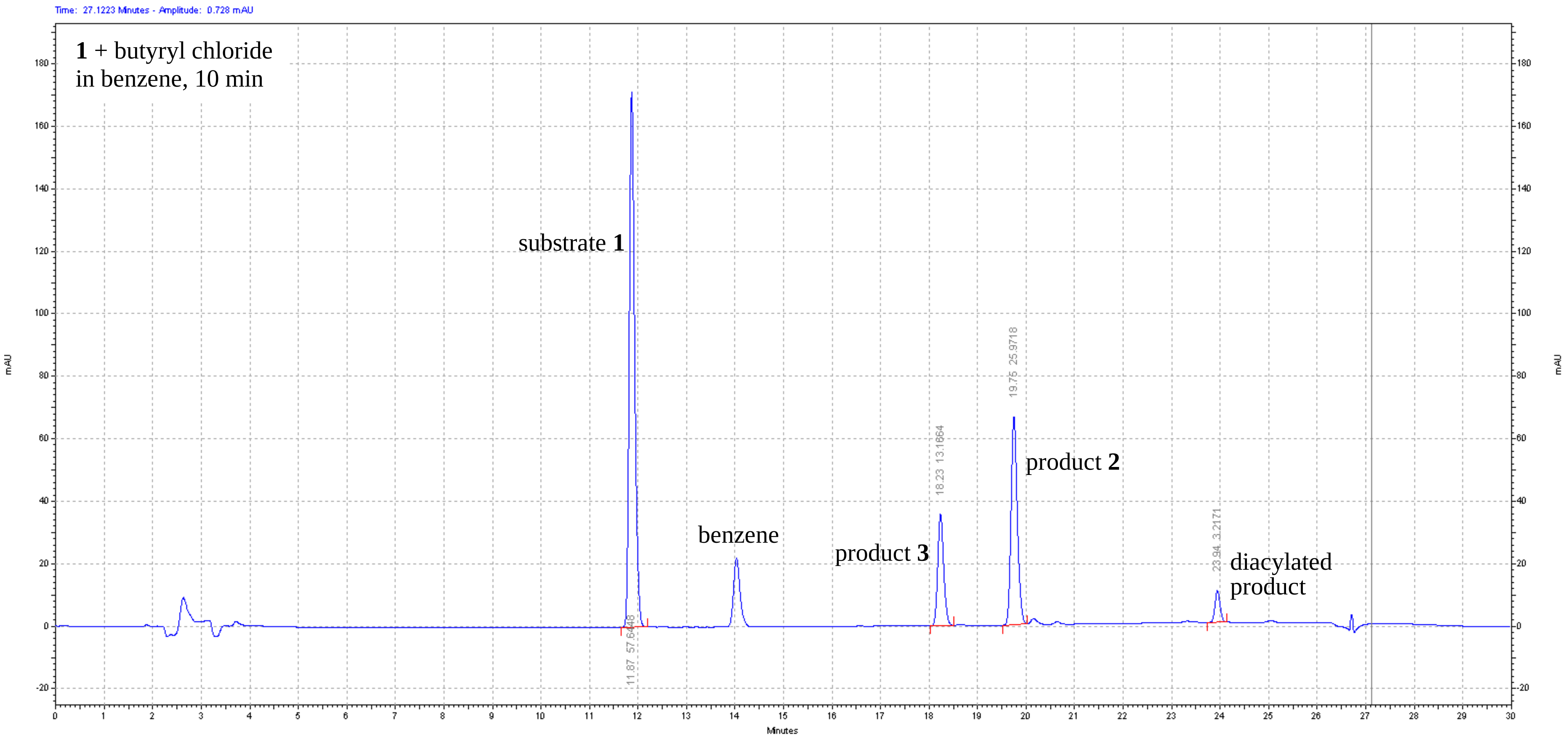
Time: 27.1223 Minutes - Amplitude: 0.728 mAU
1 + butyryl chloride
in benzene, 10 min
substrate 1
benzene
product 3
product 2
diacylated
product
11.87 57.6448
18.23 13.1664
19.75 25.9718
23.94 3.2171
mAU
Minutes

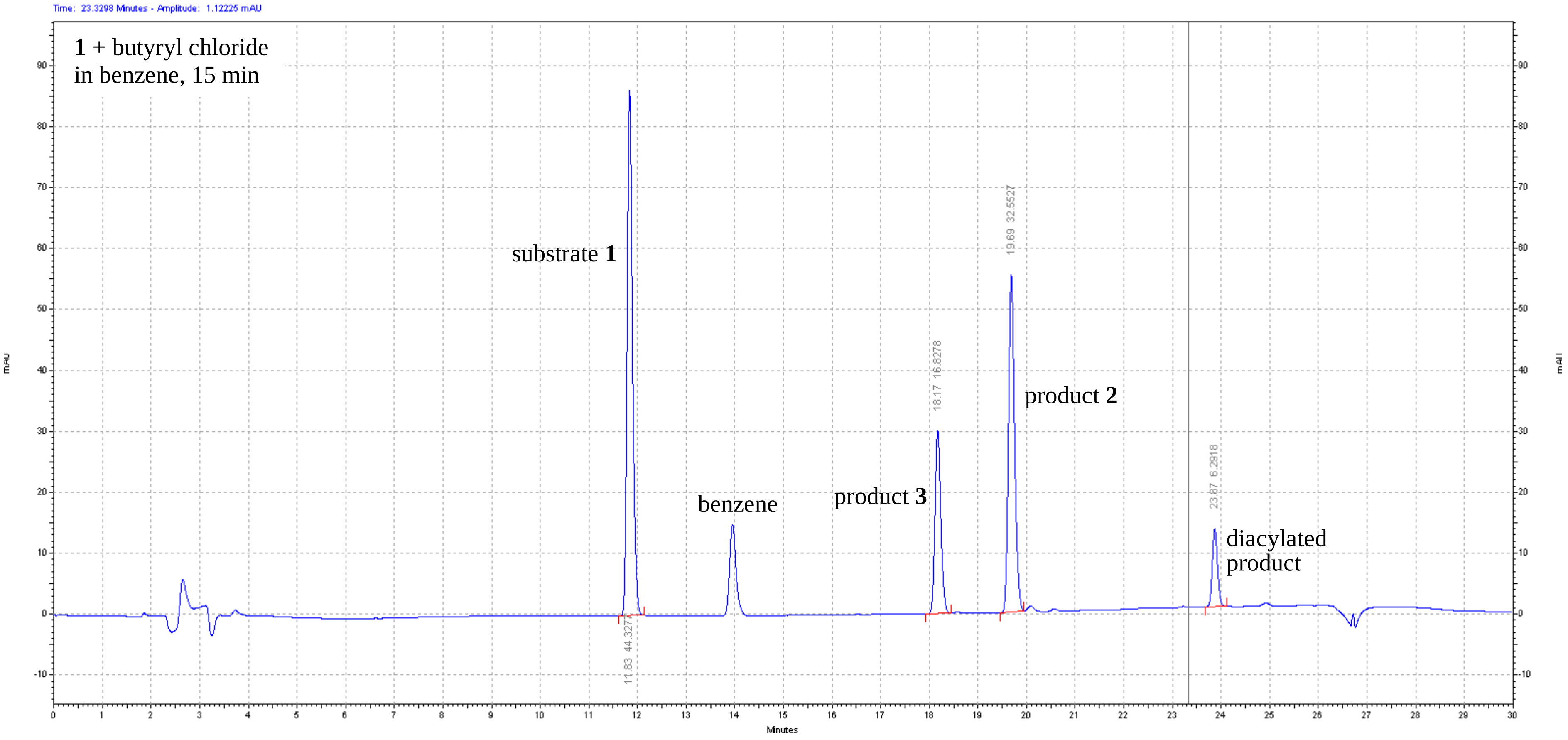
Time: 23.3298 Minutes - Amplitude: 1.12225 mAU
1 + butyryl chloride
in benzene, 15 min
substrate 1
benzene
product 3
product 2
diacylated
product
11.83 44.3277
18.17 16.8278
19.69 32.5527
23.87 6.2918
mAU
Minutes

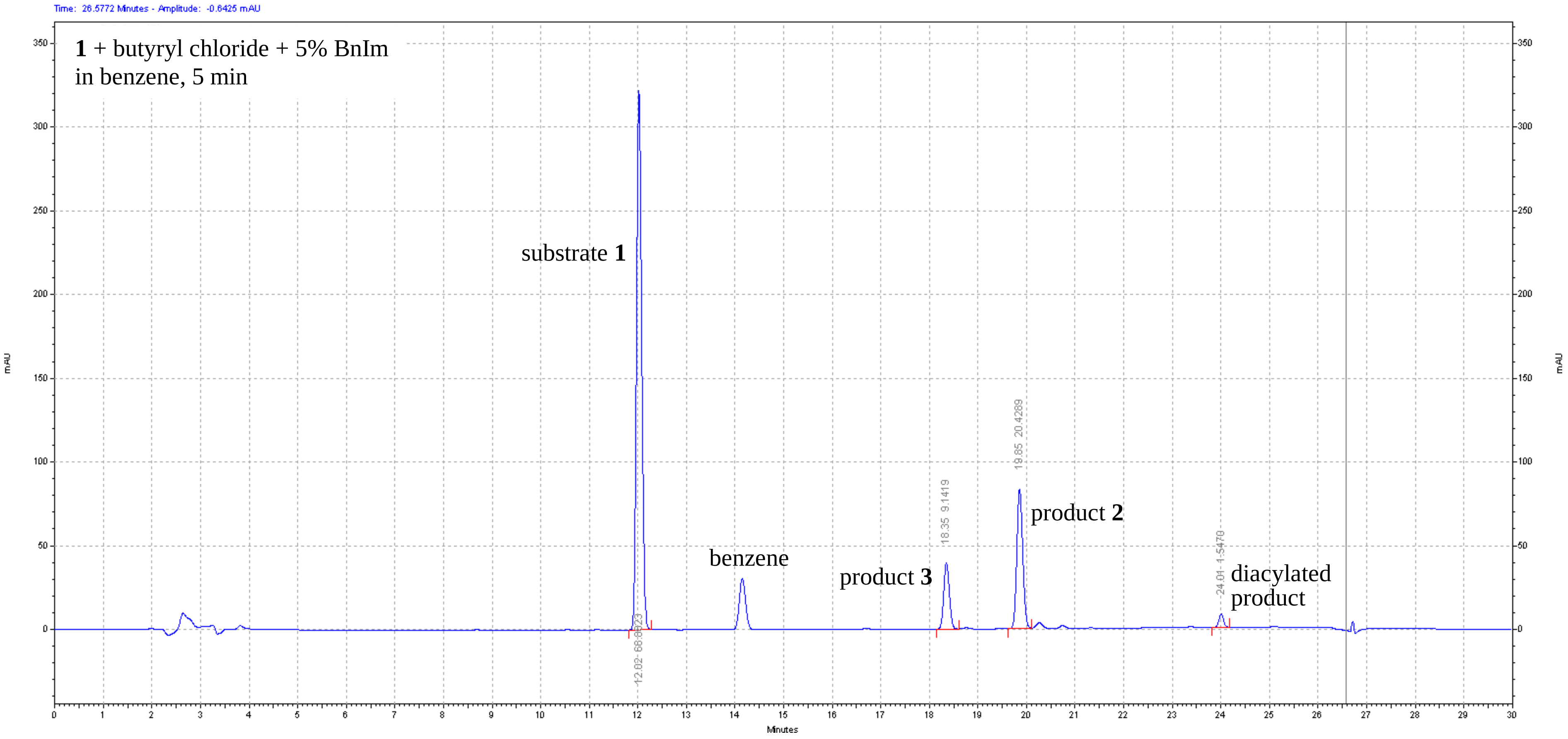
Time: 26.5772 Minutes - Amplitude: -0.6425 mAU
1 + butyryl chloride + 5% BnIm
in benzene, 5 min
substrate 1
benzene
product 3
product 2
diacylated
product
12.02 68.8823
18.35 9.1419
19.85 20.4289
24.01 1.5470
mAU
Minutes

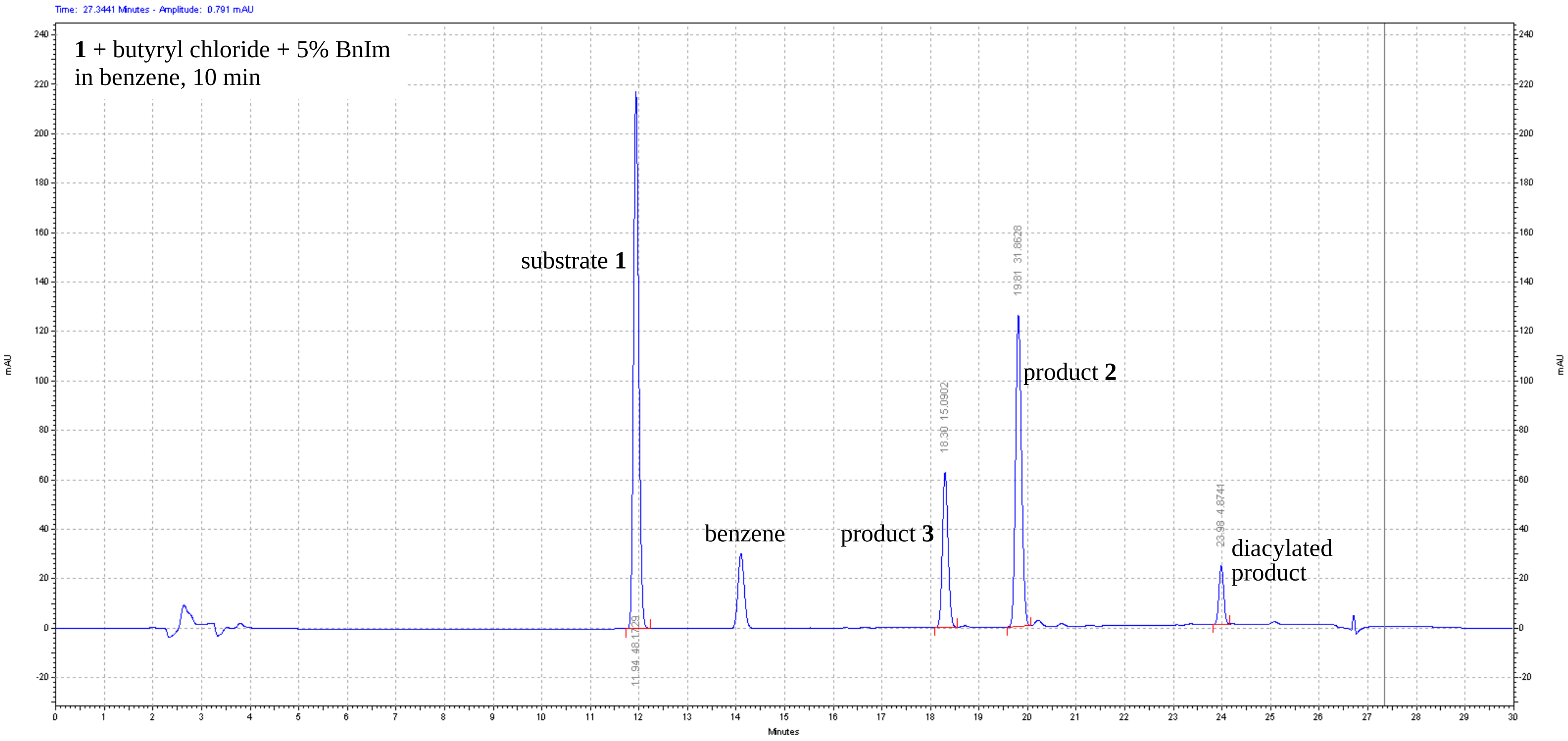
Time: 27.3441 Minutes - Amplitude: 0.791 mAU
1 + butyryl chloride + 5% BnIm
in benzene, 10 min
substrate **1**
benzene
product **3**
product **2**
diacylated
product
11.94 48.1729
18.30 15.0902
19.81 31.8628
23.98 4.8741
mAU
Minutes

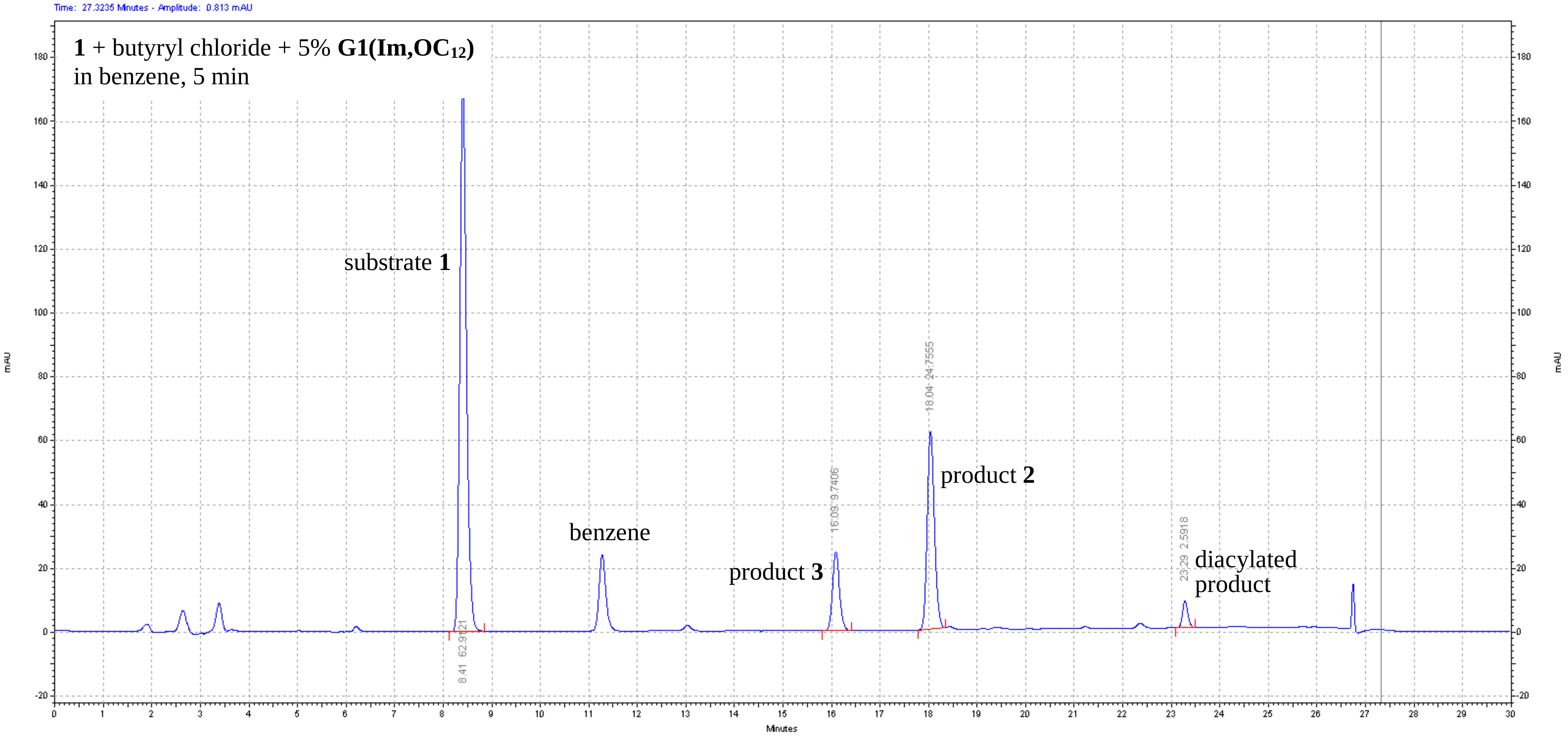

Time: 27.3235 Minutes - Amplitude: 0.813 mAU
1 + butyryl chloride + 5% G1(Im,OC12)
in benzene, 5 min
substrate 1
benzene
product 3
product 2
diacylated
product
8.41 62.9121
16.09 9.7406
18.04 24.7555
23.29 2.5918
mAU
Minutes

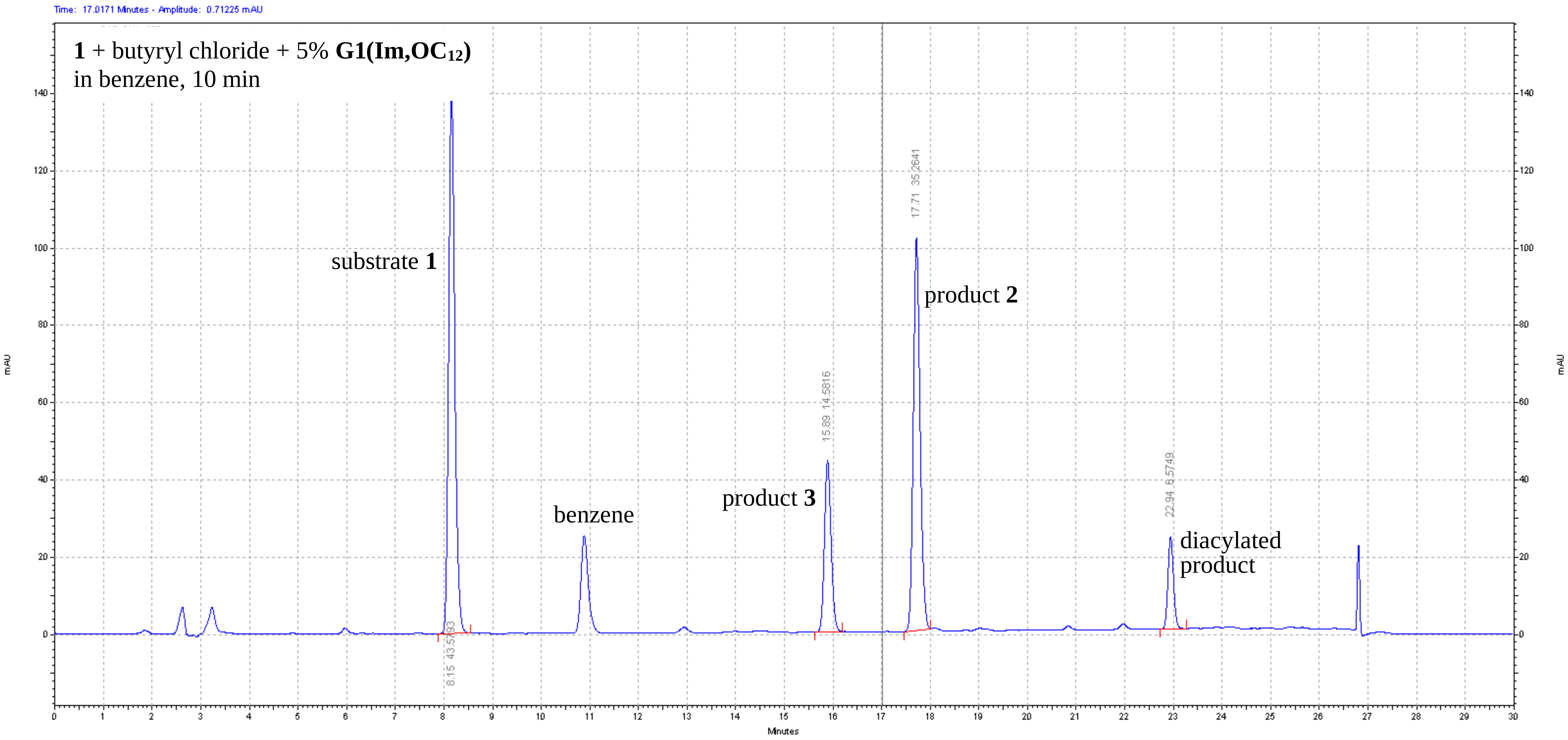
Time: 17.0171 Minutes - Amplitude: 0.71225 mAU
1 + butyryl chloride + 5% G1(Im,OC12)
in benzene, 10 min
substrate 1
benzene
product 3
product 2
diacylated
product
8.15 43.5793
15.89 14.5816
17.71 35.2641
22.94 6.5749
mAU
Minutes

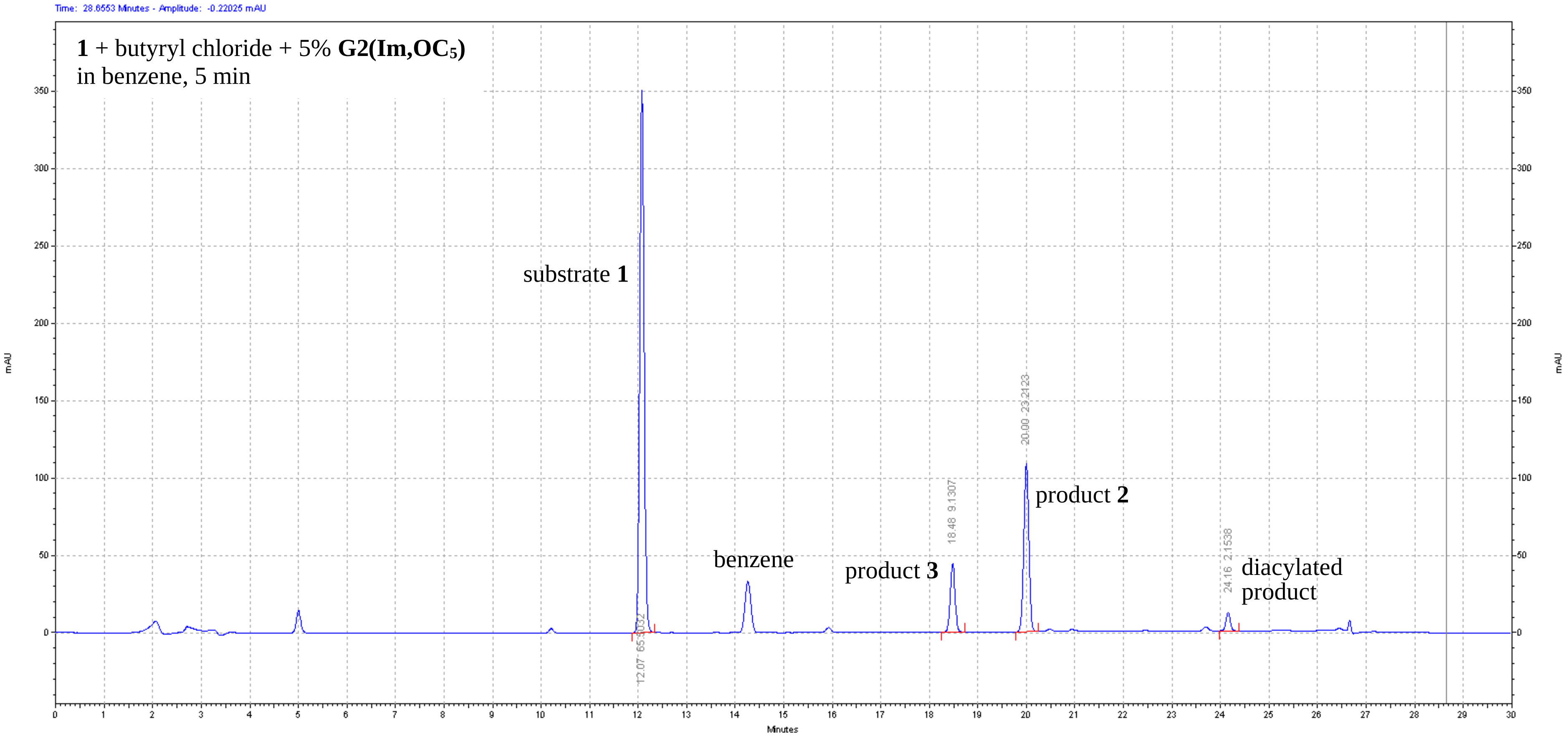
Time: 28.6553 Minutes - Amplitude: -0.22025 mAU
1 + butyryl chloride + 5% G2(Im,OC5)
in benzene, 5 min
substrate 1
benzene
product 3
product 2
diacylated
product
12.07 65.3032
18.48 9.1307
20.00 23.2123
24.16 2.1538
mAU
Minutes

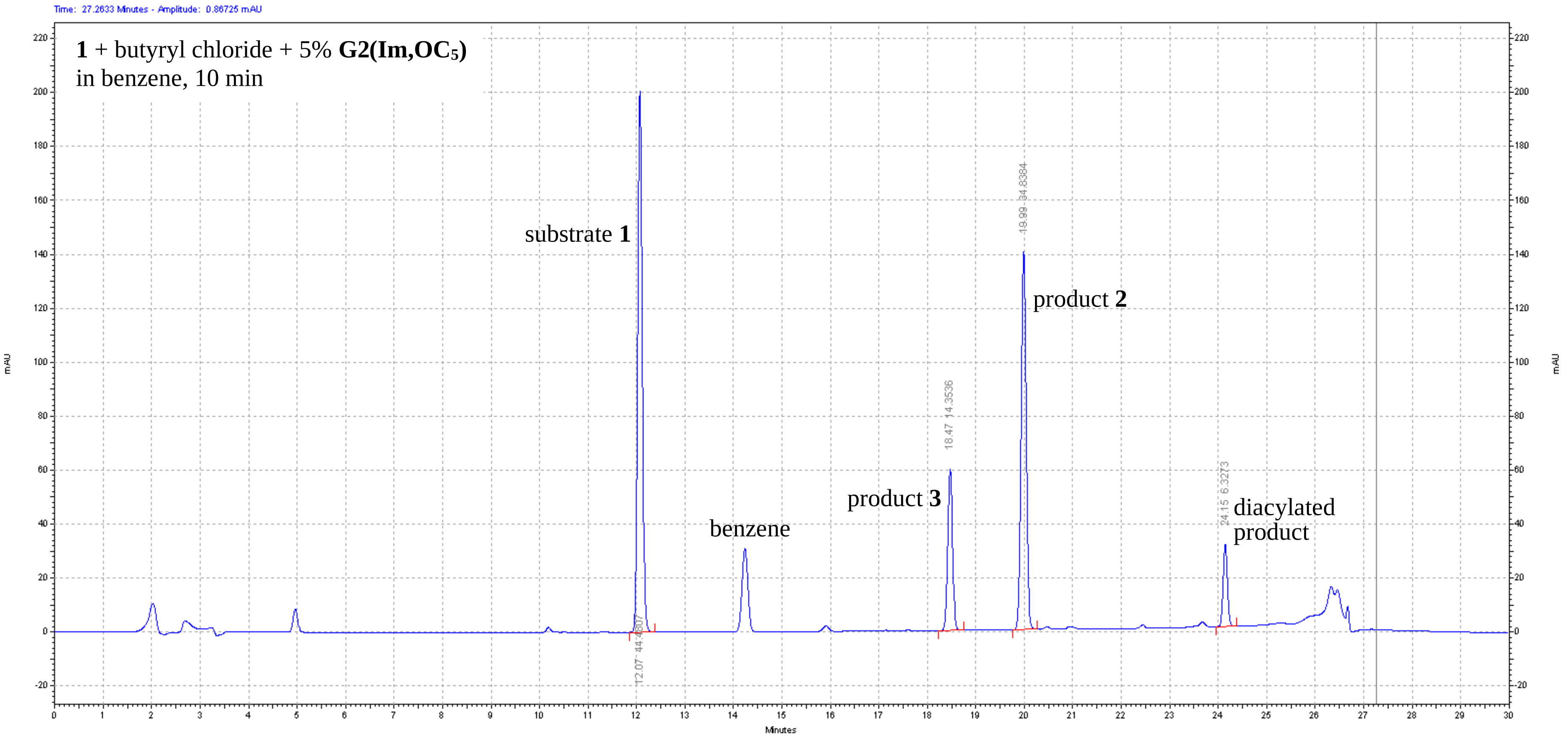
Time: 27.2633 Minutes - Amplitude: 0.86725 mAU
1 + butyryl chloride + 5% G2(Im,OC5)
in benzene, 10 min
substrate 1
benzene
product 3
product 2
diacylated
product
12.07 44.4607
18.47 14.3536
19.99 34.8384
24.15 6.3273
mAU
Minutes

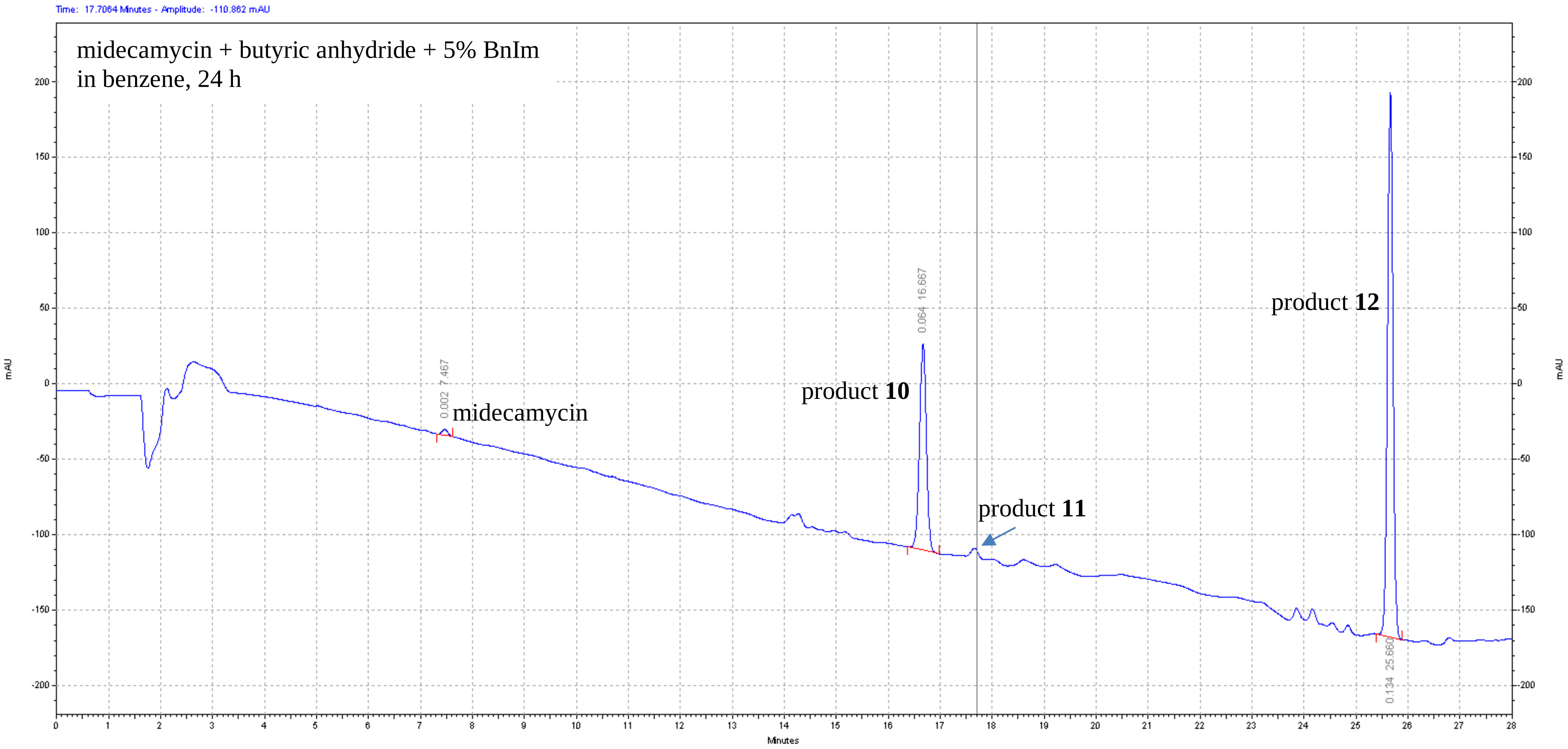

* the integrations were extracted manually due to faulty printout

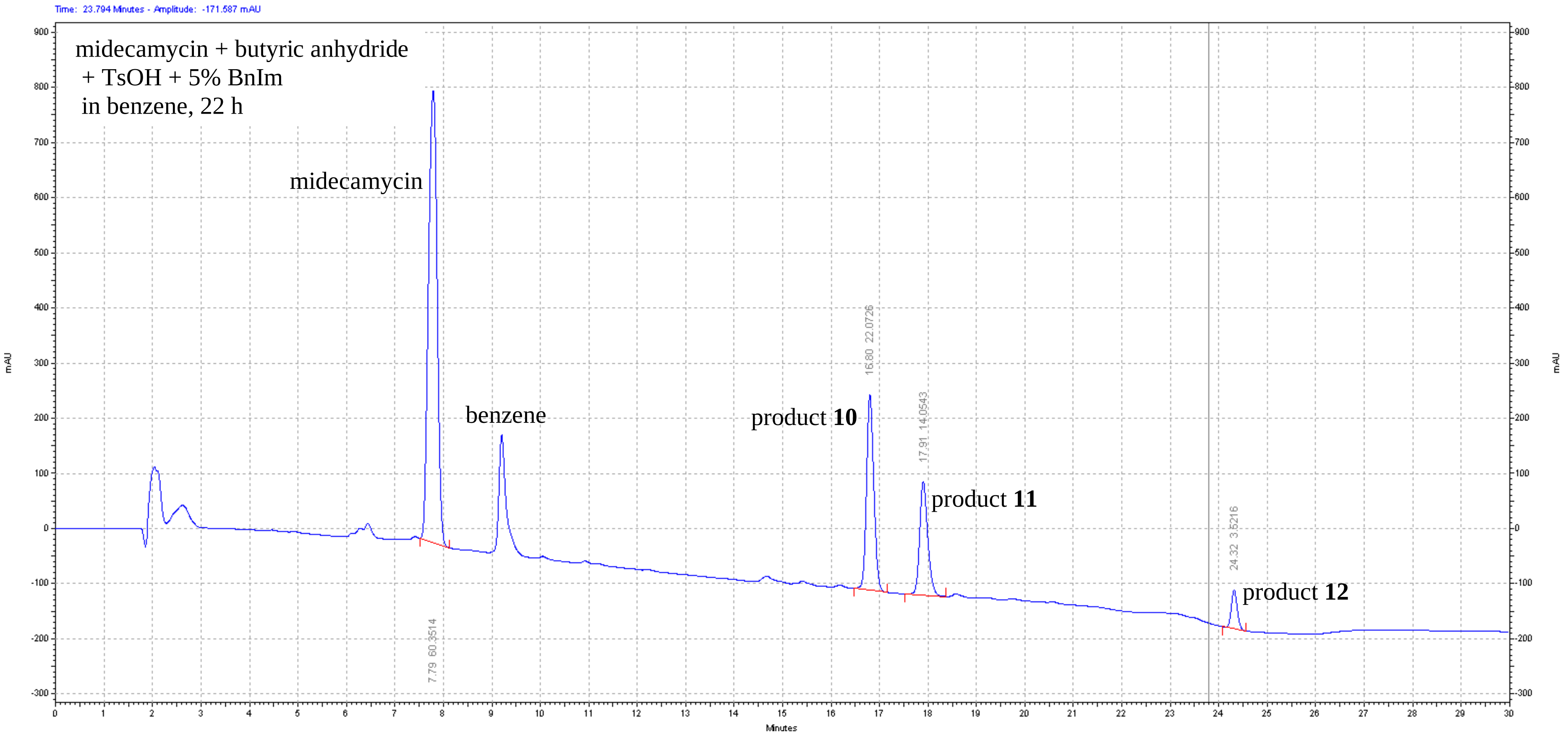
Time: 23.794 Minutes - Amplitude: -171.587 mAU
midecamycin + butyric anhydride
+ TsOH + 5% BnIm
in benzene, 22 h
midecamycin
benzene
product 10
product 11
product 12
16.80 22.0726
17.91 14.0543
24.32 3.5216
7.79 60.3514
mAU
Minutes

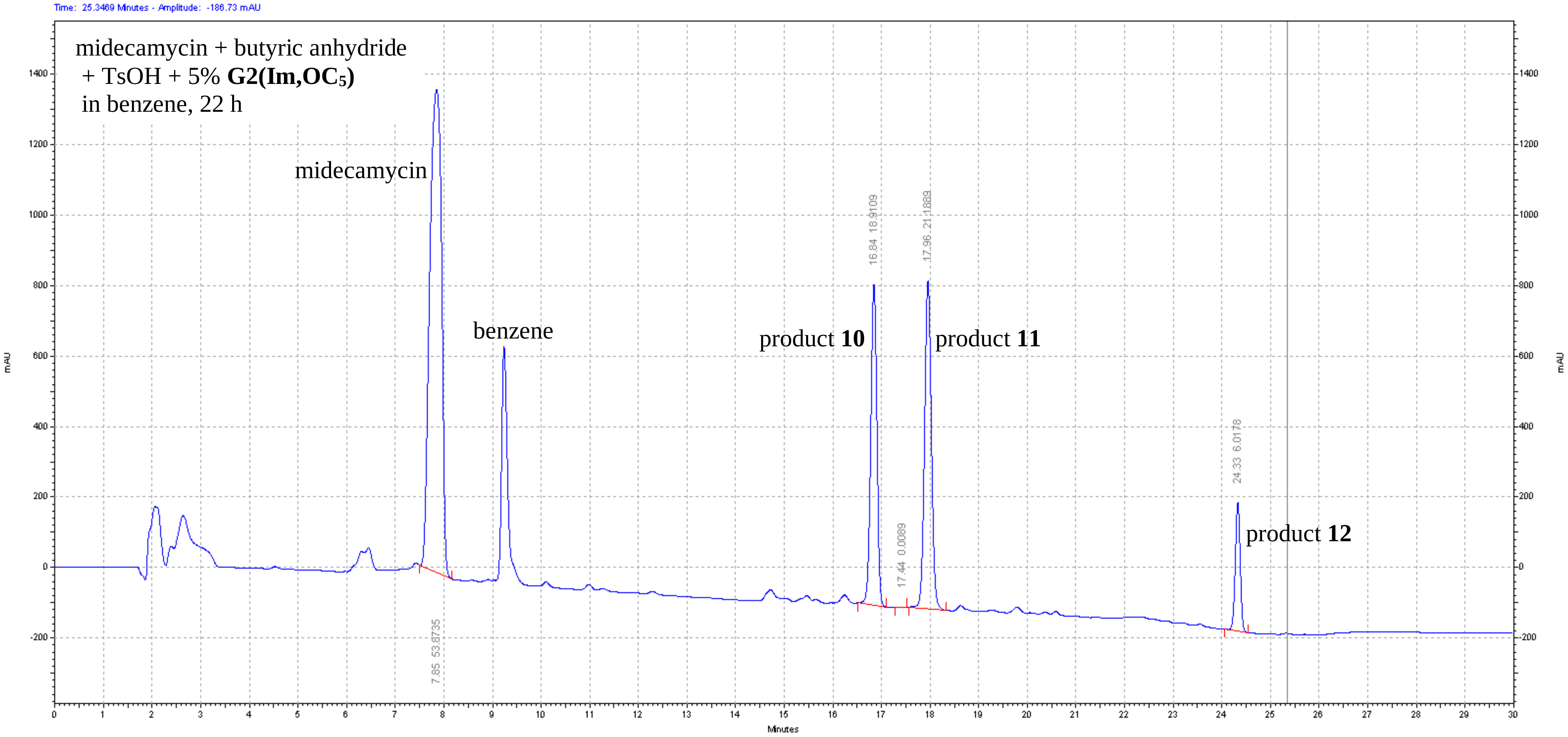

Time: 25.3469 Minutes - Amplitude: -186.73 mAU
midecamycin + butyric anhydride
+ TsOH + 5% G2(Im,OC5)
in benzene, 22 h
midecamycin
benzene
product 10
product 11
product 12
16.84 18.9109
17.96 21.1889
17.44 0.0089
24.33 6.0178
7.85 53.8735
mAU
Minutes

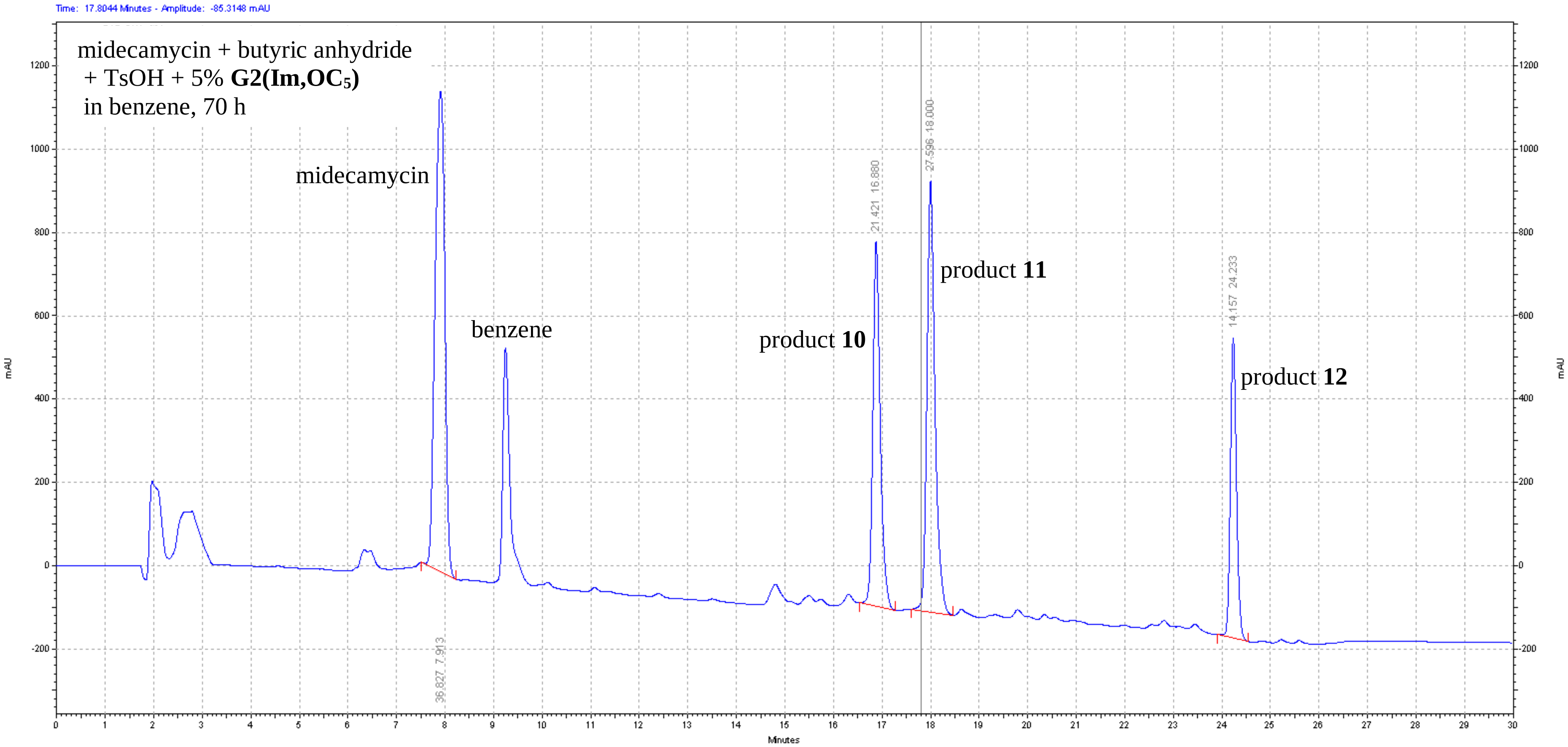
Time: 17.8044 Minutes - Amplitude: -85.3148 mAU
midecamycin + butyric anhydride
+ TsOH + 5% G2(Im,OC5)
in benzene, 70 h
midecamycin
benzene
product 10
product 11
product 12
mAU
Minutes

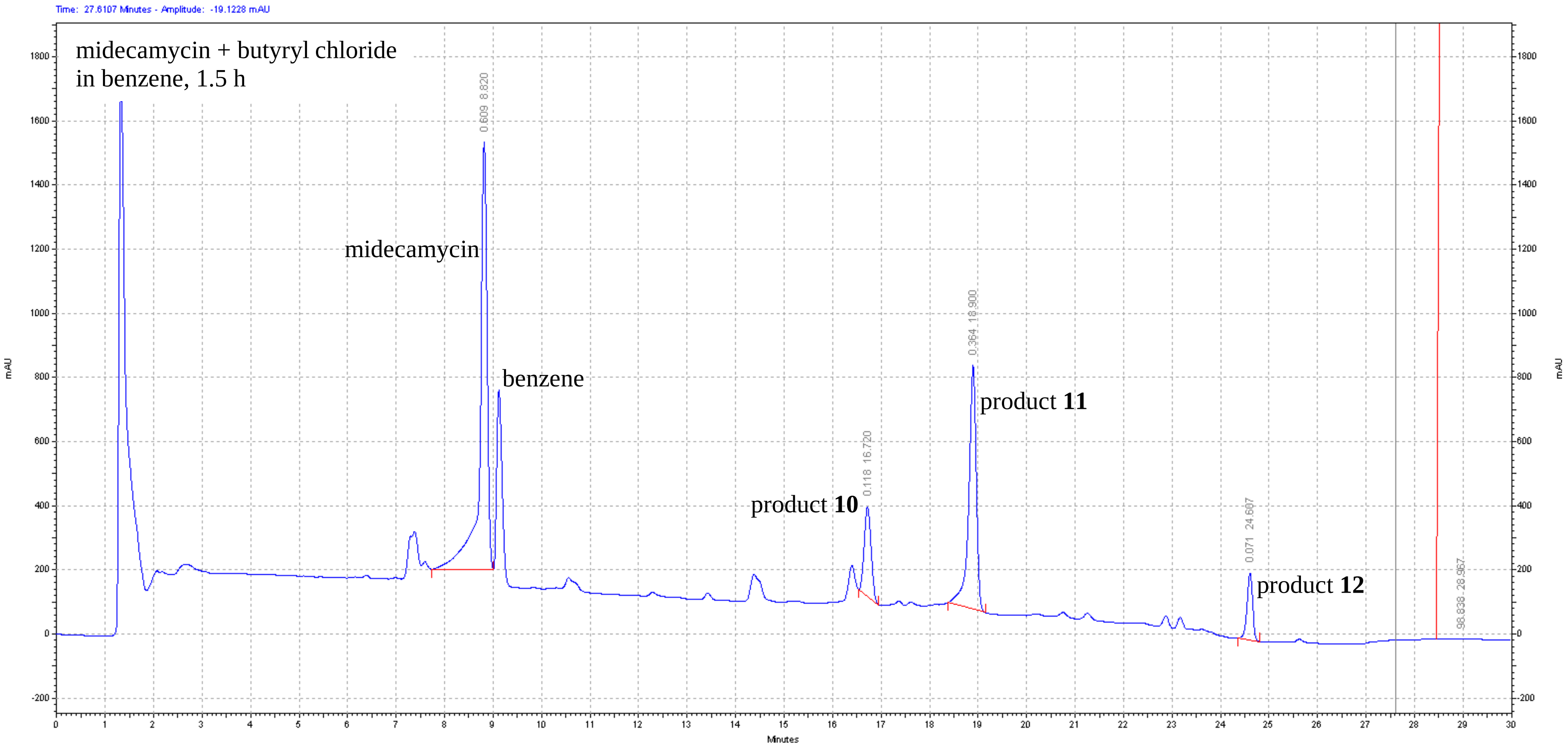

* the integrations were extracted manually due to faulty printout

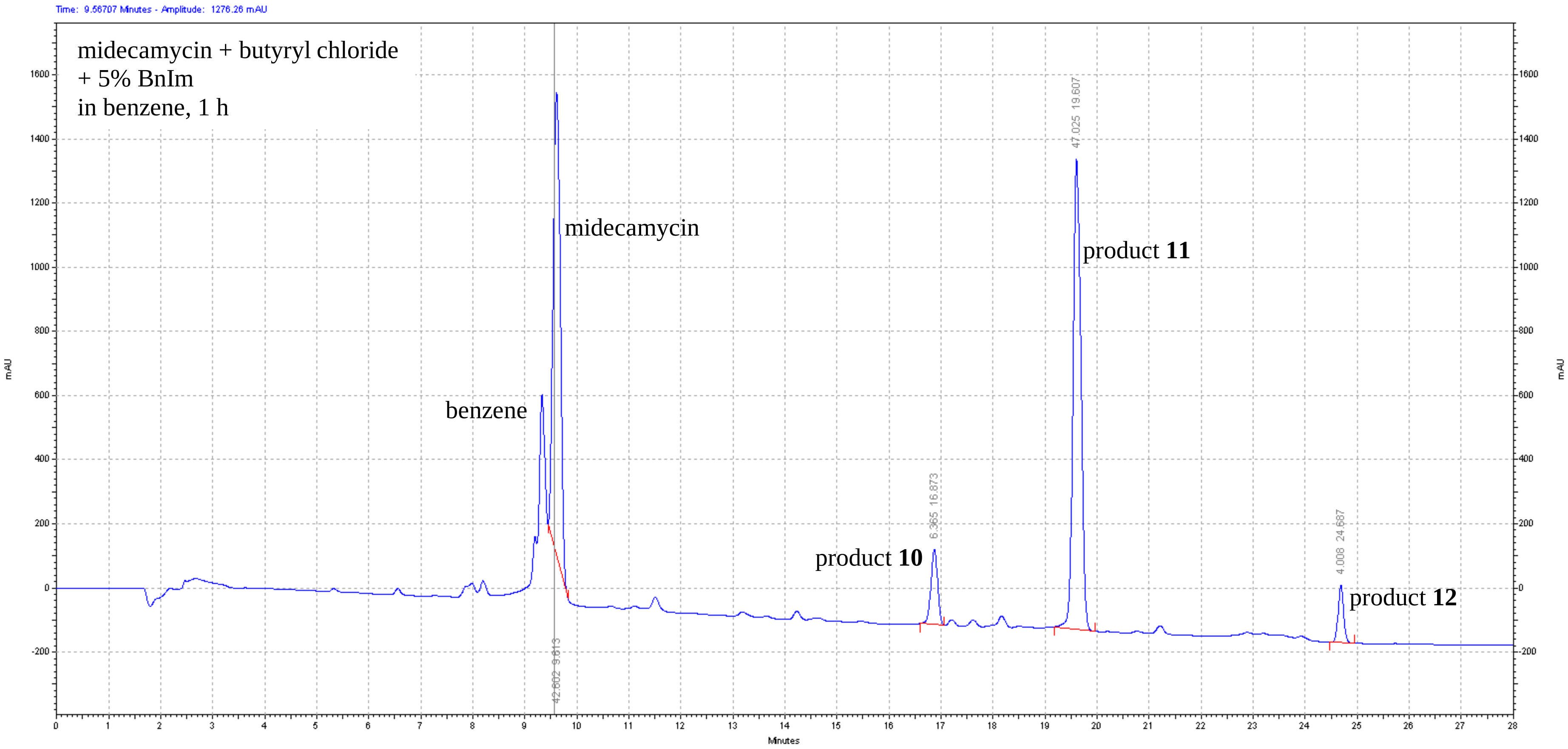

Time: 9.56707 Minutes - Amplitude: 1276.26 mAU
midecamycin + butyryl chloride
+ 5% BnIm
in benzene, 1 h
midecamycin
benzene
product 10
product 11
product 12
42.602 9.613
6.365 16.873
47.025 19.607
4.008 24.687
mAU
Minutes

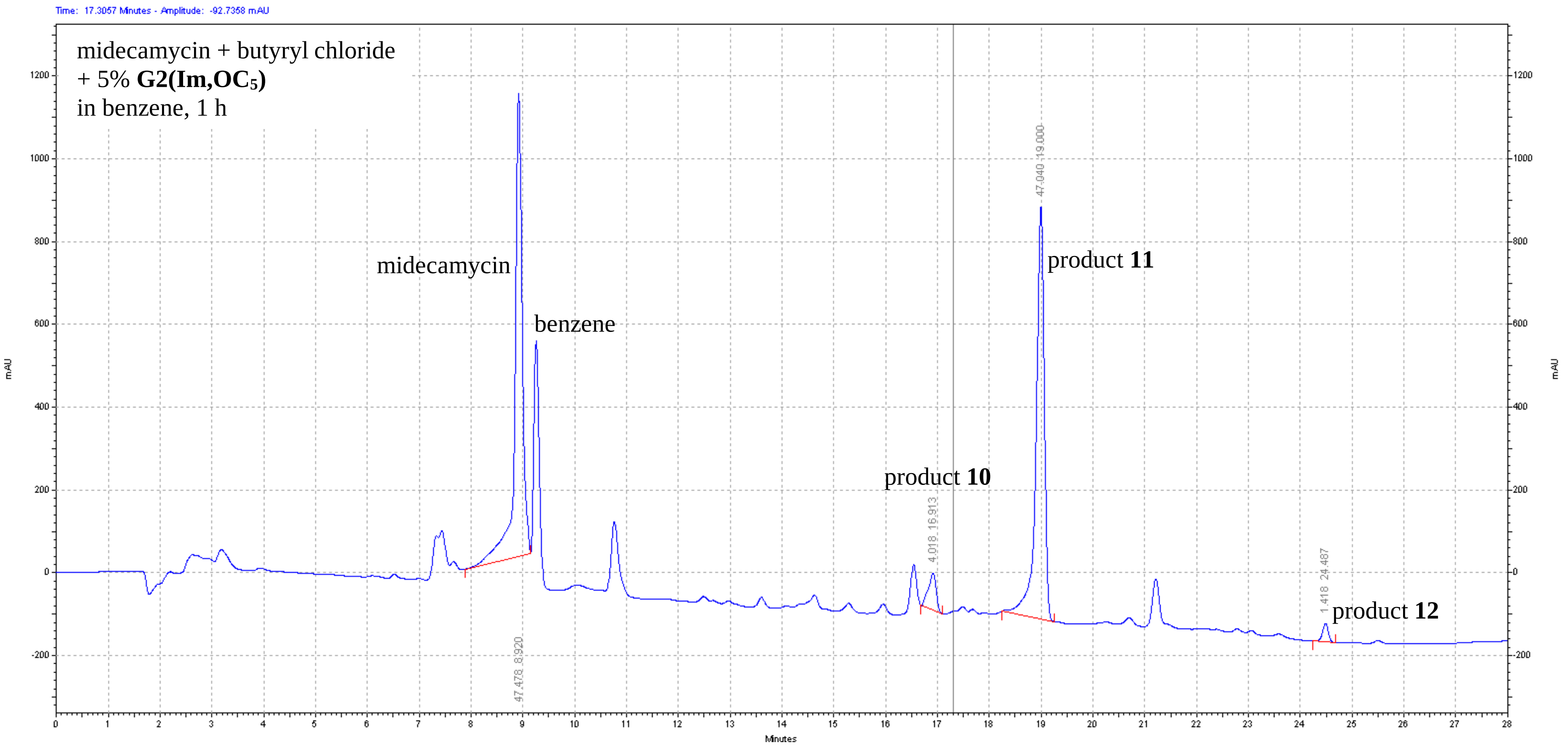
Time: 17.3057 Minutes - Amplitude: -92.7358 mAU
midecamycin + butyryl chloride
+ 5% G2(Im,OC5)
in benzene, 1 h
midecamycin
benzene
product 10
product 11
product 12
47.478 8.920
4.018 16.913
47.040 19.000
1.418 24.487
mAU
Minutes
1200
1000
800
600
400
200
0
-200
0
1
2
3
4
5
6
7
8
9
10
11
12
13
14
15
16
17
18
19
20
21
22
23
24
25
26
27
28
